# MicroRNA‐Induced Gene Silencing (MIGS): A Tool for Multi‐Gene Silencing and Targeting Viruses in Plants

**DOI:** 10.1111/pbi.70401

**Published:** 2025-10-06

**Authors:** Marie‐Emilie A. Gauthier, Kylie Shand, Satomi Hayashi, Peter M. Waterhouse, Roberto A. Barrero, Felipe F. de Felippes

**Affiliations:** ^1^ eResearch, Research Infrastructure, Academic Division QUT Brisbane Queensland Australia; ^2^ Centre for Agriculture and the Bioeconomy, Institute for Future Environments Queensland University of Technology (QUT) Brisbane Queensland Australia; ^3^ ARC Centre of Excellence for Plant Success in Nature & Agriculture, QUT Brisbane Queensland Australia

**Keywords:** gene silencing, MIGS, RNAi, small RNA, virus protection

## Abstract

Since its discovery, RNA interference (RNAi, also known as gene silencing) has been a key tool to downregulate gene expression in plants for a range of applications, including protection against viruses. Many of these applications require the silencing of multiple genes concomitantly. Multi‐gene silencing, however, can be difficult to achieve owing to challenges in generating single RNAi constructs targeting unrelated genes or due to molecular instability linked to those constructs. Here, we show that microRNA‐induced gene silencing (MIGS) can overcome many of these limitations and can be an important tool for multi‐gene silencing in plants. We demonstrate how MIGS targeting several genes enhances the RNAi‐based inhibition of one or more viruses. We also define several key features for optimising the use of MIGS in plants, including modular design, effective targeting length and phased first‐base composition.

## 1 Introduction

Gene silencing or RNA interference (RNAi) is an important mechanism of downregulation of gene expression. It is mediated by 20–24 nt small RNAs (sRNA) produced by DICER‐LIKE (DCL) enzymes from double‐stranded RNA (dsRNA) molecules. These sRNAs are loaded into ARGONAUTE (AGO) proteins to form RNA‐induced silencing complexes (RISCs), which mostly cleave RNA transcripts (post‐transcriptional gene silencing; PTGS) or guide the epigenetic repression of DNA (transcriptional gene silencing; TGS) with sequences showing complementarity to the sRNAs (Bologna and Voinnet 2014; Eamens et al. 2008).

The discovery of RNAi has revolutionised our ability to downregulate gene expression and has become an essential part of modern biology. Most silencing constructs contain one targeting sequence and aim to downregulate a single gene or a gene family possessing high sequence similarity. Simultaneous silencing of unrelated genes (with no or limited sequence similarity) using molecular constructs harbouring several distinct sequences has been less explored. Nonetheless, there are several applications for which the concomitant silencing of multiple genes is advantageous or necessary, and a good example is the protection of plants against viruses. sRNA‐producing constructs have been used to confer viral protection for over two decades (Wang et al. 2000). However, targeting a single region of a virus genome does not always give full immunity (Cillo and Palukaitis 2014; Zhang et al. 2015). Targeting several viral genes simultaneously can give more robust protection. This was well illustrated by an examination of citrus tristeza virus resistance in transgenic citrus plants (Batuman et al. 2006; López et al. 2010; Muniz et al. 2012). Plants containing hairpin RNAi (hpRNAi) constructs targeting a single region of the viral p23 or the p25 gene failed to show immunity. In contrast, plants transformed with an hpRNAi construct containing a fusion of p23, p25, p20 sequences displayed complete resistance to the virus (Soler et al. 2012). Multi‐gene silencing can also improve the longevity of the RNAi protection by hindering the appearance of strains that mutate to escape sRNA targeting (Carbonell et al. 2019) and provides an attractive approach to defend plants against different viruses at the same time (Arif et al. 2012; Bucher et al. 2006; Chung and Palukaitis 2011; Namgial et al. 2019; Niu et al. 2006).

Silencing multiple unrelated genes with a single construct can be challenging, especially when using popular methods such as hpRNAi, virus‐induced gene silencing (VIGS) and artificial microRNA (amiRNA). Instead of generating a population of sRNAs (like in VIGS and hpRNAi), amiRNA precursors are processed to produce a single 21‐nt sRNA duplex (Schwab et al. 2006). This characteristic makes the design of a single 21‐nt molecule targeting several unrelated genes very difficult and, in some cases, impossible. To overcome this, complex polycistronic precursors coding for multiple 21‐nt amiRNAs have been used, particularly to confer resistance to viruses in plants (Fahim et al. 2012; Kis et al. 2016; Kung et al. 2012; Miao et al. 2021; Park et al. 2009). Multi‐gene silencing can be achieved with VIGS and hpRNAi by fusing different sequences of interest in a single chimeric sequence (CS). However, the use of longer inserts for VIGS is problematic, either due to space constraints of the viral capsid or due to the instability of large sequences (Dommes et al. 2019; Zulfiqar et al. 2023), limiting the number of transcripts that can be introduced in the viral genome. The size of the CS can also be an issue for hpRNAi constructs. The requirement for CSs to be cloned in both orientations can easily result in large molecules, a feature linked to plasmid instability and low efficiency in plant transformation. In addition to size, inverted repeats are also linked to plasmid instability due to the higher chances of molecule recombination (Cillo and Palukaitis 2014; Oliveira et al. 2009; Park et al. 2000).

**FIGURE 1:**
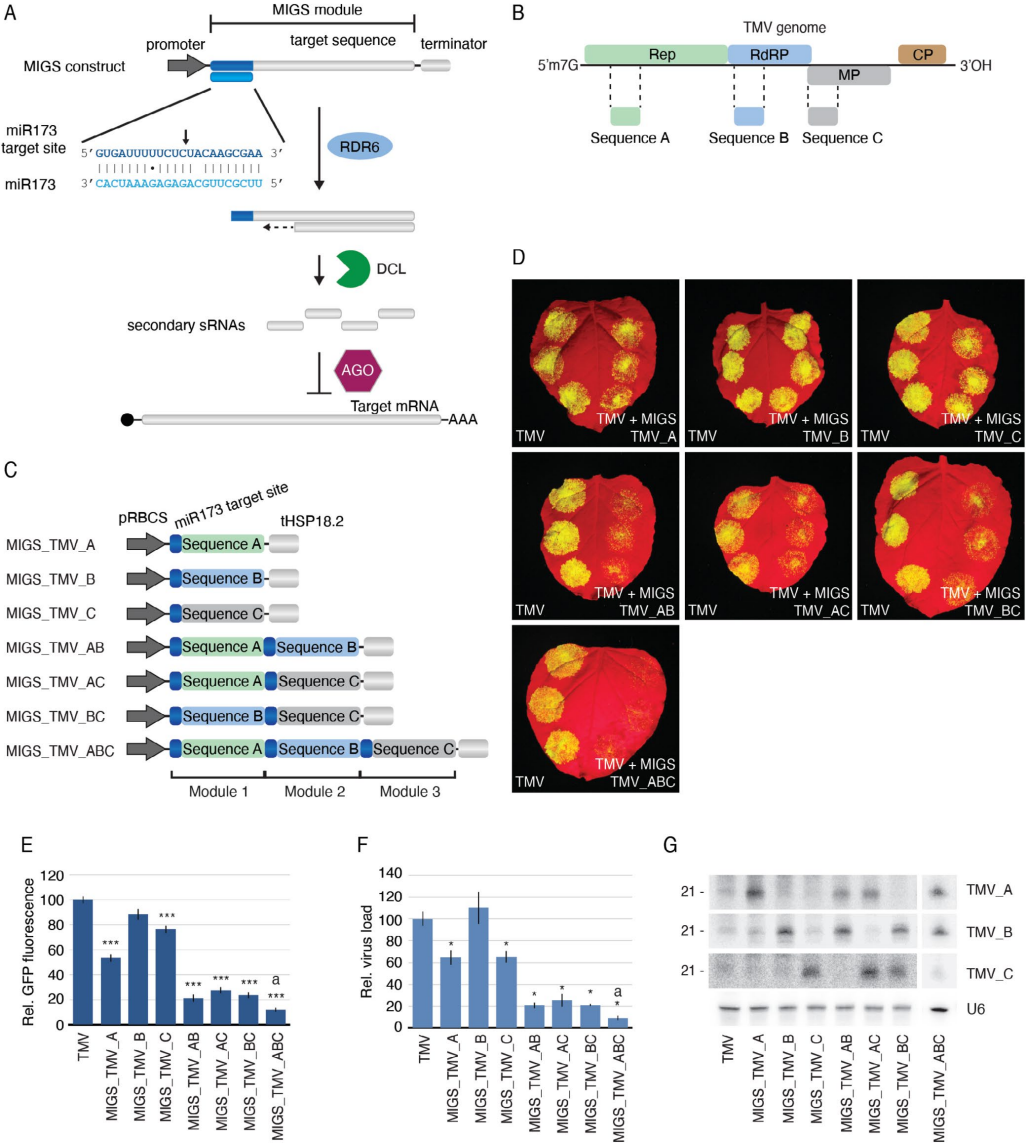
Silencing multiple genes with MIGS. (A) Diagram of the MIGS concept. A MIGS construct consists of the miR173 target site followed by a sequence derived from the target gene (referred to here as the MIGS module). The interaction of miR173 with the MIGS transcript results in the production of secondary sRNAs, which will promote silencing of complementary sequences. The arrow indicates the miRNA cleavage site. (B) Scheme of the TMV genome showing four ORFs coding for the replicase (Rep), RNA‐dependent RNA polymerase (RdRP), movement (MP), and capsid proteins (CP). Sequences used to generate the MIGS constructs are indicated (sequences A–C). (C) MIGS constructs carrying one, two, three modules, each one targeting different regions of the TMV genome. Each MIGS module consists of the miR173 binding site and the appropriate target sequence. The promoter (pRBCS) and terminator (tHSP18.2) driving expression of the MIGS cassette are shown. (D) *N. benthamiana* leaves agroinfiltrated with the TMV‐GFP infectious clone alone (left) or combined with one of the MIGS constructs and the miR173 (right). (E) Relative GFP fluorescence of 12 infiltration spots. (F) Relative virus load measured by RT‐qPCR. Standard error bars are shown. Statistically significant differences between TMV‐infiltrated and MIGS‐treated samples are indicated by “*” (*p* ≤ 0.05) and “***” (*p* ≤ 0.001); while the difference between MIGS_TMV_ABC and the other MIGS constructs is shown by “a” (*p* ≤ 0.001, Mann–Whitney *U* test). (G) Northern blot to detect migsiRNAs originating from different modules. U6 was used as load control.

MicroRNA‐induced gene silencing (MIGS) is an alternative approach for silencing genes based on the ability of some microRNAs (miRNAs), a class of sRNAs originating from endogenous transcripts with foldback structures, to induce the production of a new wave of sRNAs (secondary sRNAs). One of such miRNAs is miR173, which targets non‐coding transcripts known as *TAS1* and *TAS2*. Different from most miRNAs, miR173‐mediated cleavage does not result in direct degradation of its target. Instead, the interaction of miR173 with *TAS1* and *TAS2* mRNAs leads to the recruitment of RNA‐DEPENDENT RNA POLYMERASE 6 (RDR6), which uses the target transcript as a template for the synthesis of a dsRNA, which is then processed by DCLs into largely phased 21 nt sRNAs with a periodicity determined by the miRNA cleavage (de Felippes 2019; de Felippes and Waterhouse 2020). MIGS exploits this mechanism using a simple design (Figure 1A) that consists of a sequence derived from the target gene fused in its 5′ end to the sequence recognised by miR173 (referred to here as the MIGS initiator) (de Felippes 2012; de Felippes et al. 2012). Once expressed, MIGS transcripts targeted by miR173 will give rise to secondary sRNAs, which will silence transcripts with complementary sequences. Two unrelated genes can be simultaneously silenced with a single MIGS construct when different sequences, each flanked by the miR173 target site, are organised in tandem (de Felippes et al. 2012). This modular design and the shorter sequence requirement than in hpRNA overcome some of the limitations associated with classic RNAi approaches, suggesting that MIGS could be the method of choice for multi‐gene silencing. Here, we demonstrate the ability of a single MIGS construct to drive the downregulation of several genes simultaneously and investigate the spectrum breadth and potency of this approach against endogenes and viruses.

## 2 Results

### 2.1 Silencing of Multiple Genes With MIGS Severely Impacts Virus Accumulation in Plants

A distinct characteristic of MIGS is the simplicity of its design. The basic MIGS construct consists of the miR173 target site followed by a sequence derived from the target gene. In this work, we refer to this arrangement as the MIGS module (Figure 1A). It was previously shown that two modules, each carrying distinct target sequences, can be arranged in tandem to give rise to a single MIGS molecule to be used to silence two unrelated genes. This modular design was more efficient than having a single miR173 target site followed by concatenated target sequences (de Felippes et al. 2012). Thus, to explore the potential of MIGS as a tool for silencing multiple genes in plants, we generated a series of single‐, double‐ and triple‐module MIGS constructs targeting ~300 nt regions of one, two or three viral genes (Figure 1B,C). These constructs (pMIGS_TMV_A‐C, pMIGS_TMV_AB, pMIGS_TMV_AC, pMIGS_TMV_BC and pMIGS_TMV_ABC) target the replicase (Rep), and/or RNA‐dependent RNA polymerase (RdRP) and/or movement protein (MP) genes of the tobacco mosaic virus (TMV) and were used to assess MIGS' impact on virus accumulation (Figure 1B,C). Each MIGS construct was agroinfiltrated as spots into *Nicotiana benthamiana* leaves with a TMV infectious clone containing a GREEN FLUORESCENT PROTEIN (GFP) cassette (pTMV‐GFP) (Lindbo 2007) and pmiR173 (the MIGS initiator).

All the MIGS constructs reduced the GFP fluorescence in their agro‐infiltrated spots, indicating that they all inhibited TMV accumulation, albeit to varying degrees (Figure 1D–G). The triple‐, double‐, single‐module constructs were most, middle, least effective, respectively. The higher efficiency of the triple‐module MIGS construct targeting all three genes (pMIGS_TMV_ABC) compared to a construct carrying three repetitions of the same MIGS module targeting the replicase (pMIGS_TMV_AAA) suggests that besides the increased total levels of migsiRNAs (sRNAs originating from a MIGS molecule), the broader spectrum of migsiRNAs produced from the double‐ and triple‐module constructs also contributes to their effectiveness (Figure S1). Interestingly, the single‐module construct targeting the RdRP region (TMV_B) appeared to have little effect on virus accumulation when used alone but significantly enhanced viral inhibition when incorporated into a double‐ or triple‐module construct (Figure 1D–F).

### 2.2 A Single MIGS Construct Can Inhibit Different Viruses by Targeting Multiple Sequences Simultaneously

**FIGURE 2:**
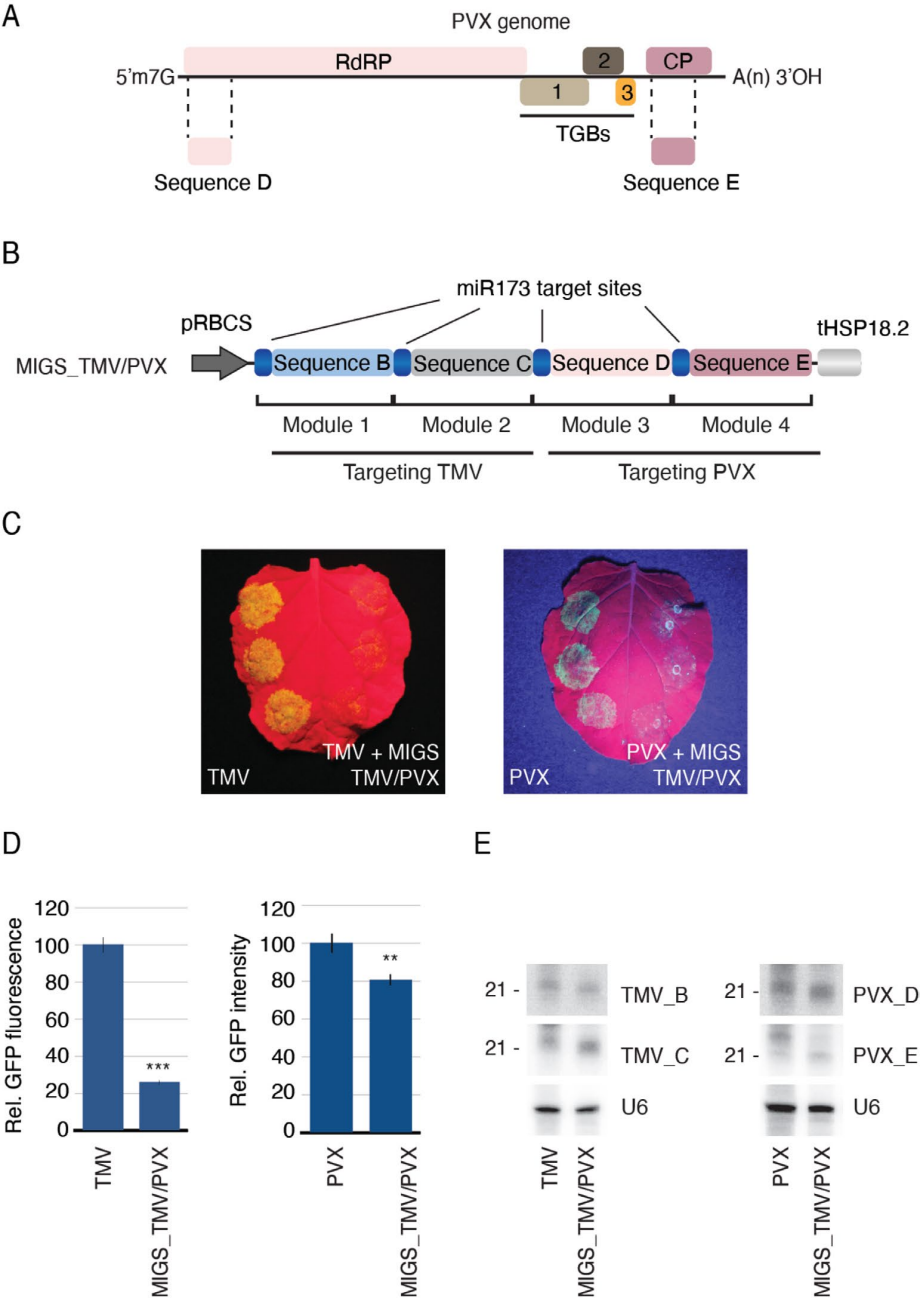
A single MIGS construct to target different viruses simultaneously. (A) Graphic representation of the PVX genome showing the RNA‐dependent RNA polymerase (RdRP), triple gene blocks (TGB 1–3) and the capsid proteins (CP) genes. Sequences used for the MIGS construct are indicated (sequences D, E). (B) Overview of the MIGS_TMV/PVX construct targeting TMV and PVX viruses. (C) Agroinfiltrated *N. benthamiana* leaves showing the effect of the MIGS treatment (right side of the leaves) on TMV or PVX expression. (D) Relative GFP fluorescence (for TMV, 12 infiltration spots) and pixel intensity of GFP signal (for PVX, 16 infiltration spots) are shown. Statistically significant changes in GFP expression are indicated by “**” and “***” (*p* ≤ 0.01 and 0.001, respectively, Mann–Whitney *U* test). (E) migsiRNAs originating from the different modules as detected by northern blot assays. U6 was used as a loading control.

In the field, plants are exposed to a variety of viruses and their variants, which often occur as complexes. Therefore, a treatment capable of simultaneously protecting against multiple viruses or variants represents a significant advancement in crop protection. To test the potential of a single MIGS construct to confer such broad‐spectrum resistance, we generated pMIGS_TMV/PVX, a construct comprising the modules targeting the RdRP and MP genes from TMV, along with two additional modules, one targeting the RdRP and coat protein (CP) of potato virus X (PVX, Figure 2A,B). Two sets of plants were agroinfiltrated with pMIGS_TMV/PVX and pmiR173. One set was co‐infiltrated with pTMV‐GFP, and the other with our PVX‐GFP infectious clone (pPVX‐GFP) (Barton et al. 2017). In both cases, there was a visible reduction in GFP fluorescence (Figure 2C–E), indicating that this four‐module construct effectively inhibited both viruses. These results suggest that MIGS may offer a generally applicable strategy for multi‐virus protection in plants.

### 2.3 migsiRNAs Are Generated From All 15 Modules of a Single MIGS Construct, but Efficiency Varies Depending on the Module Location

**FIGURE 3:**
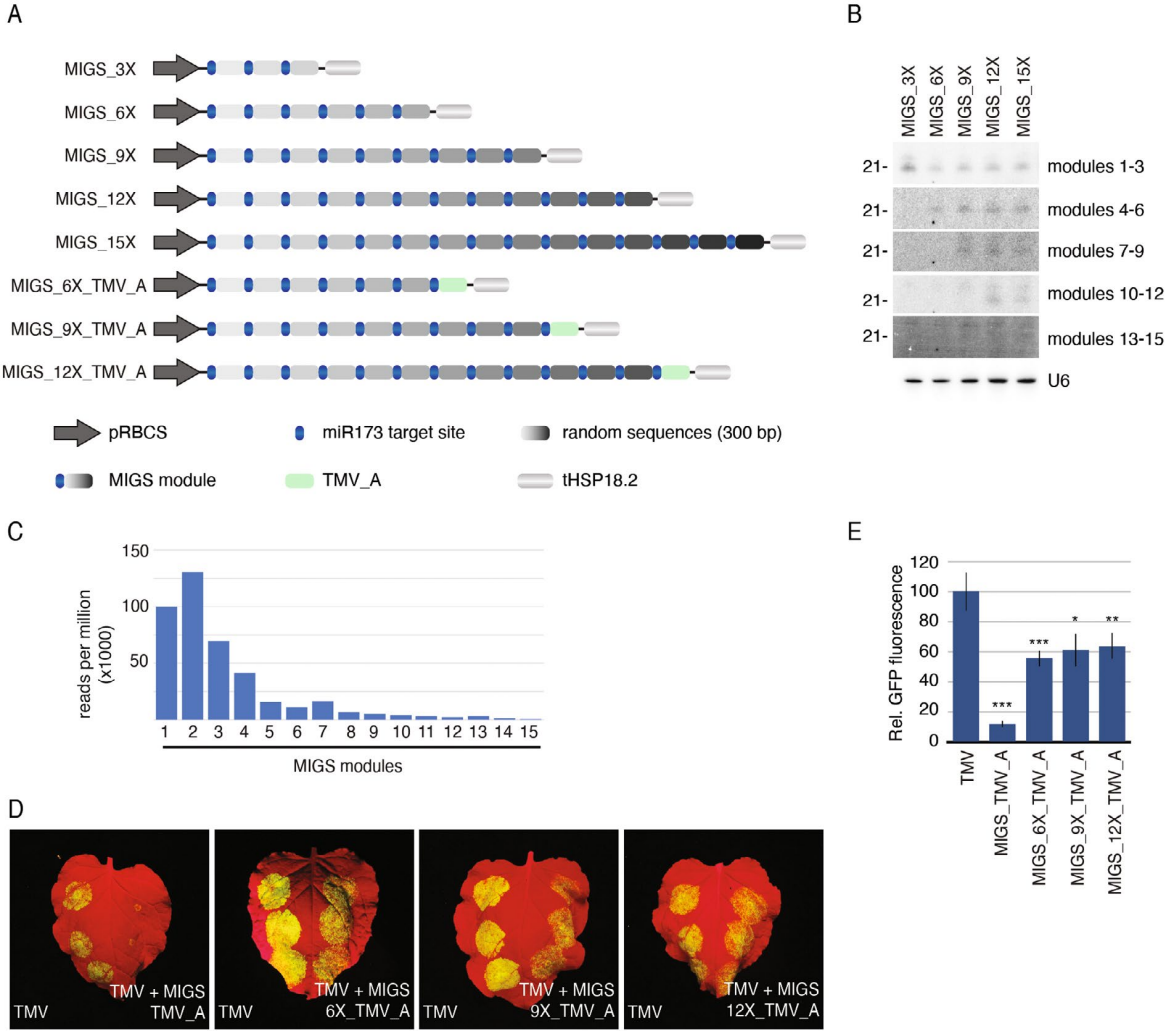
Testing the multi‐gene silencing range of MIGS. (A) Illustration showing the collection of constructs generated to test the maximum range of a single MIGS molecule. (B) Northern blots to detect migsiRNAs originating from different locations in a MIGS construct. Probes were designed to detect migsiRNAs coming from groups of three modules. (C) migsiRNA deep‐sequencing showing the production of migsiRNAs along the MIGS_15X molecule. (D) Images showing the silencing efficiency of MIGS against TMV when migsiRNAs originate from downstream modules and (E) their relative GFP fluorescence levels (10 infiltration spots). To improve silencing efficiency, a single plasmid carrying the MIGS and the miR173 expression cassettes was used instead of co‐infiltration with different *Agrobacteria*. “*”, “**”, “***” indicate statistically significant differences with *p* ≤ 0.05, 0.01, 0.001, respectively (Mann–Whitney *U* test). The standard error bars are given.

To determine the maximum number of modules a single MIGS construct can carry while still producing detectable levels of migsiRNAs, we generated constructs containing three, six, nine, twelve and fifteen modules (pMIGS_3X, pMIGS_6X, pMIGS_9X, pMIGS_12X, pMIGS_15X, respectively; Figure 3A). Each module consists of a unique 300 bp sequence (randomly generated) flanked in its 5′ end by the miRNA173 target site (Figure 3A), enabling precise mapping of the resulting migsiRNAs to their module of origin. The constructs were agroinfiltrated in *N. benthamiana* leaves with or without pmiR173, and the migsiRNA populations were assessed by northern blots and sRNA sequencing. The blots readily detected migsiRNAs originating from the first 12 modules; however, those from modules 13 to 15 were near the detection limit (Figure 3B). sRNA sequencing of pMIGS_15X‐infiltrated patches corroborated the blot results (Figure 3C and Table S1). Notably, most migsiRNAs mapped to the first four modules, with a sharp drop in their abundance from module five onwards, declining steadily to near background levels by module 15.

Phasing is one of the most notable features for miR173‐dependent secondary sRNAs generation (Allen et al. 2005; Montgomery, Yoo, et al. 2008). Although not a requirement for MIGS function, migsiRNAs are expected to be generated in phase with the miR173 cleavage site. Except for module 4, migsiRNA production showed a strong phased pattern (Figure S2). In seven of the 15 modules, migsiRNAs were perfectly phased with their respective miR173 cleavage site. For the remaining modules, the main phasing register was offset by one nucleotide, which is commonly observed in sRNA originating from *TAS* transcripts (Allen et al. 2005; Carbonell et al. 2014; Montgomery, Yoo, et al. 2008). We also checked the size distribution of migsiRNAs originating from pMIGS_15X. As expected from the predominance of DCL4 in processing dsRNA synthesised by RDR6, migsiRNAs mapping to each of the 15 modules were mainly 21 nt in size (Figure S3). The same pattern for phasing and size distribution of migsiRNAs can be seen for pMIGS_6X and pMIGS_9X (Figures S4 and S5), confirming that multi‐modular MIGS constructs give origin to *bona fide* migsiRNAs.

To investigate whether the decrease in sRNA production observed along multi‐modular constructs was specific to MIGS, we created hpRNAi versions of the same constructs (hpRNAi_3X, hpRNAi_6X, hpRNAi_9X, hpRNAi_12X, hpRNAi_15X; Figure S6A). These were generated by fusing the same random sequences used in the MIGS constructs into single chimeric sequences (CSs) and cloning them appropriately into hpRNAi vectors. The sRNA levels produced by the hpRNAi constructs were comparable to those from the MIGS constructs and showed a similar gradient pattern, where sRNAs originating from regions located closer to the ‘loop end’ (near the PDK intron) were more abundant than those from the other end of the dsRNA (Figure S6B).

The observed gradient of sRNA levels produced by both MIGS and hpRNAi constructs prompted us to investigate the minimum level of migsiRNA required to downregulate gene expression. To address this, we appended the TMV_A module to the 3′ end of the MIGS_6X, MIGS_9X, MIGS_12X cassettes and measured their effects on TMV accumulation (Figure 3A). As expected, these constructs were less effective than pMIGS_TMV_A alone. However, even when positioned as the 13th module, the TMV_A‐derived migsiRNAs still reduced viral accumulation by 40%–50% (Figure 3D,E).

### 2.4 Sequences as Small as 158 bp Can Direct Strong Gene Silencing With MIGS

**FIGURE 4:**
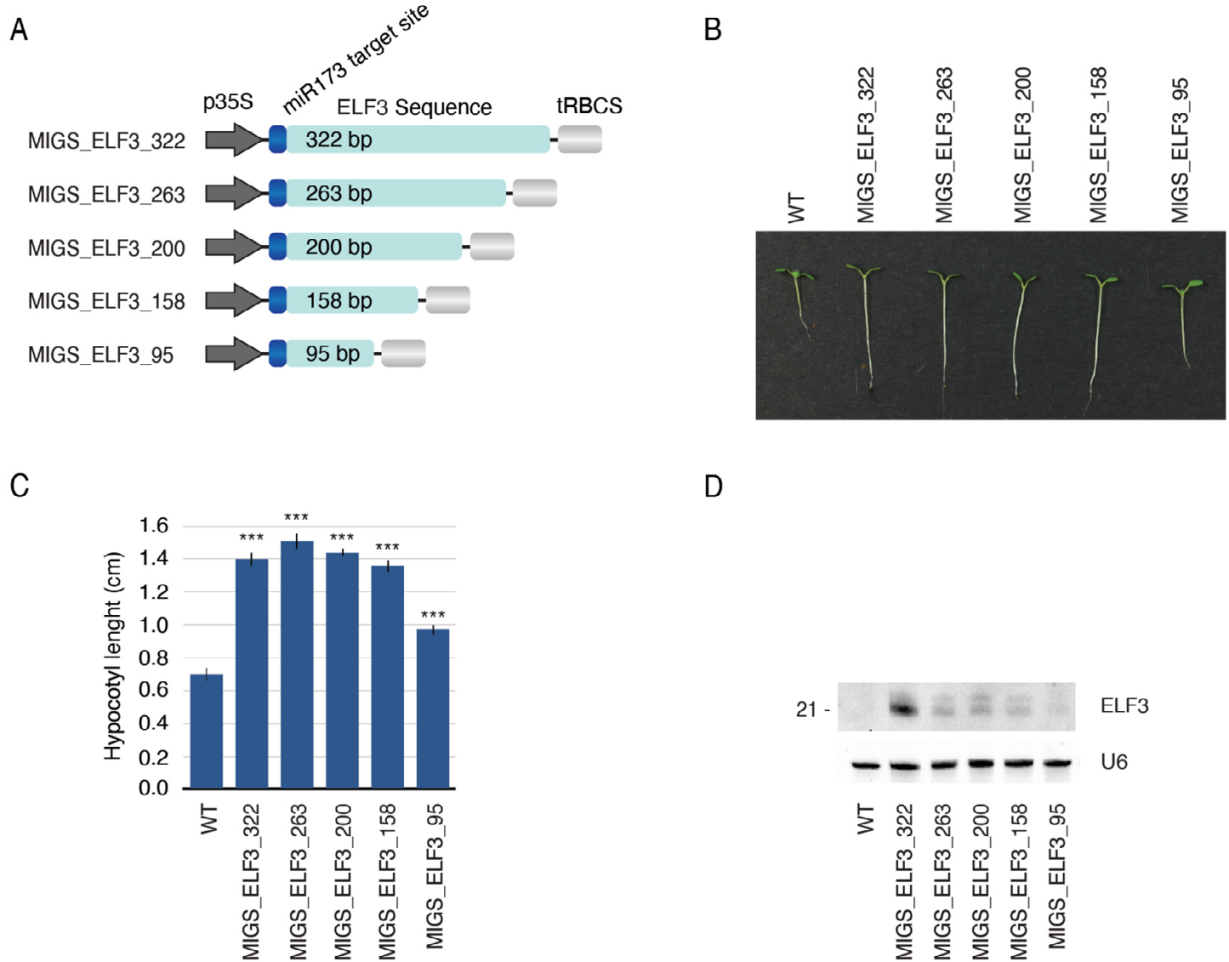
Effect of target sequence size on MIGS efficiency. (A) MIGS constructs carrying different sizes of the *ELF3* gene. (B) Arabidopsis plants showing the elongated hypocotyl phenotype associated with the silencing of *ELF3*. (C) The average hypocotyl length of 15 independent T1 plants is shown. Statistical differences were calculated using the Mann–Whitney *U* test (“***” indicates a *p* ≤ 0.001). (D) Northern blot showing the accumulation of migsiRNAs against *ELF3*. U6 was used as a loading control.

The multi‐gene silencing capacity of MIGS could be further expanded by using MIGS modules that carry CSs. For instance, two ~200 bp sequences originating from distinct genes could be fused into a single CS flanked by the MIGS initiator binding site. In this way, a three‐module MIGS construct could potentially silence six unrelated genes instead of just three. However, longer CSs may compromise silencing efficiency for genes whose target sequences are located further from the miRNA target site (de Felippes et al. 2012), most likely due to a reduction in RDR6 processivity over extended distances (Bleys et al. 2006; de Felippes and Waterhouse 2020; Moissiard et al. 2007; Petersen and Albrechtsen 2005; Vermeersch et al. 2010). Thus, for the above strategy to work, it is important to restrict the CS to the smallest size possible. Therefore, we estimate the shortest effective sequence to be used in a MIGS construct. We transformed *Arabidopsis thaliana* plants with MIGS constructs containing sequences ranging from 322 bp to 95 bp that target *EARLY FLOWERING 3* (*ELF3*) (Figure 4A). Silencing efficiency can be easily visualised since loss of *ELF3* gives longer hypocotyls under short‐day conditions (Hicks et al. 2001). Previously, a MIGS construct carrying a 322 bp *ELF3* template was demonstrated to silence this gene to a level similar to that of a null mutant (de Felippes et al. 2012). Plants transformed with MIGS constructs carrying a sequence of 322, 263, 200, 158 bp all displayed similar degrees of hypocotyl elongation, which was indicative of strong and efficient gene silencing (Figure 4B–D); a 95 bp sequence gave a weaker response. Similar results were obtained when we analysed plants expressing MIGS targeting *AGAMOUS* (*AG*, MIGS_AG). *AG* is another example of an endogenous gene that can be used as a silencing reporter. Downregulation of *AG* activity results in a flower‐within‐flower phenotype caused by the conversion of stamens and carpels into sepals and petals (Bowman et al. 1989). Phenotypes resembling *AG* knockout mutants were observed using a 158 nt sequence (MIGS_AG_158), although these were less frequent than when using MIGS constructs encoding longer sequences (Figure S7). Together, these results suggest that sequences in the range of 160–200 bp represent an ideal size to generate CS for MIGS constructs with enhanced multi‐targeting capability without losing silencing efficiency.

### 2.5 MIGS Efficiency Depends on the Identity of the First Nucleotide of the migsiRNA

**FIGURE 5:**
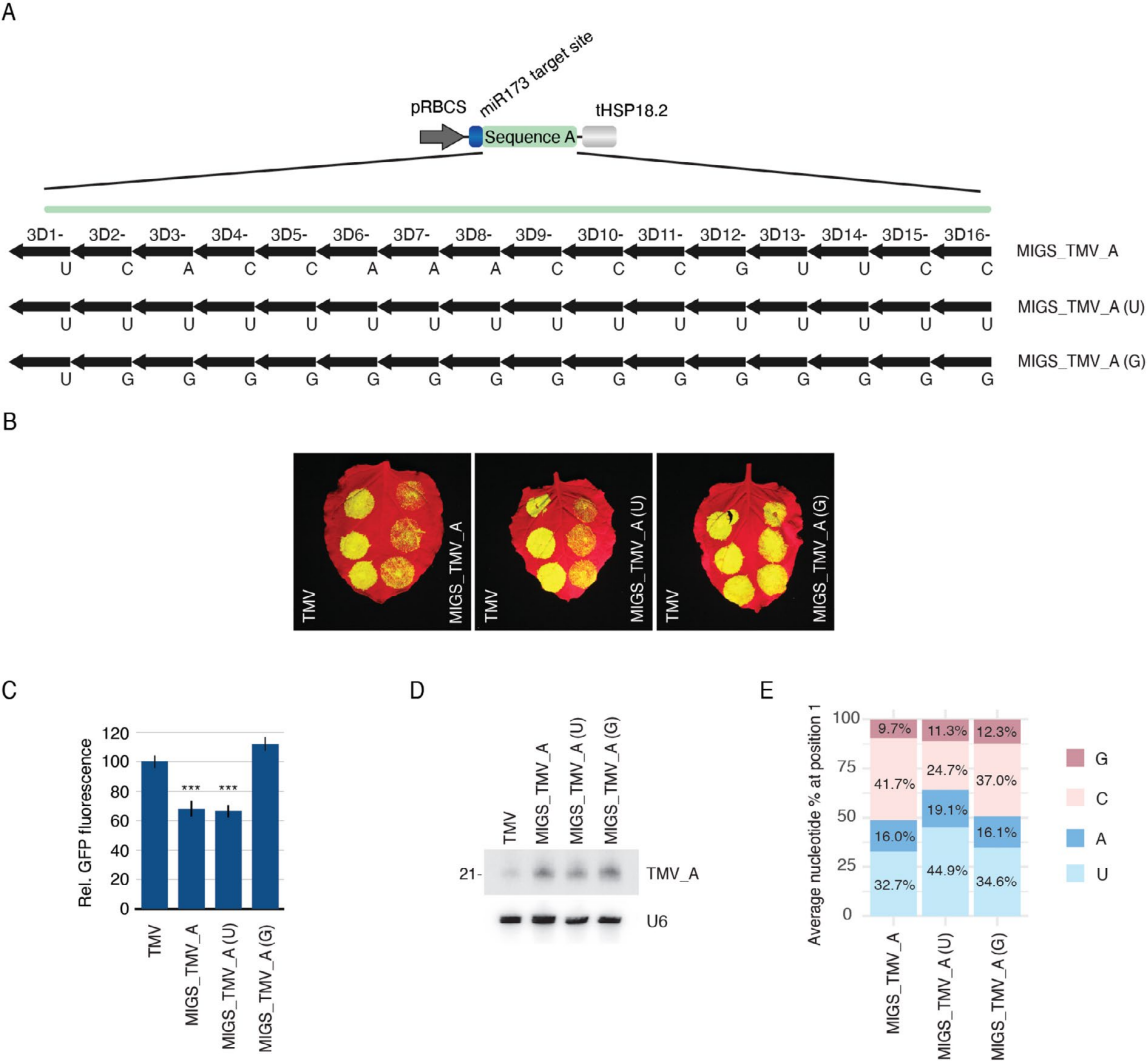
Effect of the migsiRNA 5′ end first nucleotide on MIGS efficiency. (A) MIGS constructs generated with the wild‐type target sequence (MIGS_TMV_A) or with a modified sequence where the first nucleotide at the 5′ end of all predicted phased migsiRNAs originating from the minus strand (indicated in the figure) was replaced with a U or G (MIGS_TMV_A (U) and MIGS_TMV_A (G), respectively). migsiRNA labelling (3D1‐ to 3D16‐) follows the convention for tasiRNAs nomenclature, where ‘3’ refers to migsiRNAs originating downstream of the miRNA target site, followed by its position (1–16) and the strand they come from (−). (B) Agroinfiltrated *N. benthamiana* leaves illustrating the effect of sequence mutations on the silencing efficiency of MIGS (left: TMV alone; right: TMV + MIGS). (C) Relative GFP fluorescence of at least 12 infiltration spots. “***” indicates a statistically significant difference from the control with a *p* ≤ 0.001. (D) migsiRNA accumulation detected by the northern blot of the infiltrated leaves. (E) 5′‐end nucleotide composition of sRNAs mapping to the different MIGS constructs.

To be functional, migsiRNAs need to be loaded into ARGONAUTE (AGO) proteins, the main effectors of silencing pathways. In plants, PTGS relies mostly on AGO1 and AGO2 (Bologna and Voinnet 2014). AGO5 is another protein of critical importance to PTGS. AGO5 expression is induced by several viruses, including TMV, and plays an important role in the response to viral infection (Brosseau and Moffett 2015; Tu et al. 2023). Thus, in our TMV‐based reporter system, MIGS efficiency should be dependent on migsiRNAs being associated with AGO1, AGO2, AGO5. The loading specificity of those AGOs is primarily determined by the 5′ terminal nucleotide: AGO1, AGO2, AGO5 preferentially load siRNAs with a 5′ uracil (U), adenosine (A), cytosine (C), respectively (Mi et al. 2008; Montgomery, Howell, et al. 2008; Takeda et al. 2008). This suggests that MIGS constructs producing migsiRNA populations enriched in 5′ guanosines (G) may be less effective than those producing 5′ A‐, C‐, or U‐containing migsiRNAs. To test this, we made pMIGS_TMV_A_(U) and pMIGS_TMV_A_(G) (Figure 5A). In these constructs, the first nucleotide of the predicted migsiRNAs derived from the minus strand (which will be complementary to most of the targets) was replaced by a U or G, respectively. The original MIGS_TMV_A construct is predicted to produce 16 different virus‐targeting migsiRNAs from the minus strand (Figure 5A, 3D1‐ to 3D16‐); among these, only one (3D12‐) naturally begins with a G at the 5′ end. Thus, it is expected that most migsiRNAs originating from pMIGS_TMV_A and pMIGS_TMV_A_(U) will be functional due to their interaction with AGO1, AGO2, AGO5, while migsiRNAs from pMIGS_TMV_A_(G) would be poorly loaded into those AGOs. Corroborating this view, the silencing efficiency of pMIGS_TMV_A_(U) did not differ from pMIGS_TMV_A (Figure 5B–D). In contrast, pMIGS_TMV_A_(G) lost its ability to silence TMV, despite producing similar levels of migsiRNAs. Sequence composition analysis confirms that migsiRNAs starting with a U at the 5′ end were more abundant in tissues expressing pMIGS_TMV_A_(U) compared to the pMIGS_TMV_A (44.9% versus 32.7%, respectively) (Figure 5E). This increase was to the detriment of migsiRNAs beginning with C, which account roughly for half of the predicted migsiRNAs in pMIGS_TMV_A. Nonetheless, the amount of migsiRNAs starting with a C, A, U was nearly the same among both constructs, making approximately 90% of the sRNAs mapped to the MIGS constructs. Similarly, there is an increase in migsiRNAs starting with a G in samples expressing pMIGS_TMV_A_(G) (12.3% vs. 9.7% in pMIGS_TMV_A) (Figure 5E). It is important to note that the data also include a background of virus‐derived sRNAs, for which the sequence cannot be differentiated from migsiRNAs. Given the poor efficiency of pMIGS_TMV_A_(G) in inhibiting TMV accumulation, tissues expressing this construct have a higher proportion of virus‐derived sRNAs, which complicate the precise quantification of G‐starting migsiRNAs in this sample. In addition, AGO loading and targeting interaction are known factors affecting sRNA turnover (Ji and Chen 2012). Thus, it is likely that many sRNAs with a G in the 5′‐end are degraded and not represented in sRNA libraries. Indeed, the under‐representation of migsiRNAs starting with G in pMIGS_TMV_A_(G) is illustrated by a clear shift in phasing observed in this sample, with phasing offset by 1 nt relative to the miR173 cleavage site (Figure S8). These results confirm that MIGS efficiency can be affected by the identity of the migsiRNA first nucleotide and that sequence optimisation may be necessary to enhance the effectiveness of this technology, particularly when targeting G/C‐rich genes.

### 2.6 Identification and Validation of *N. benthamiana* miRNAs to be Used as MIGS Initiators

**FIGURE 6:**
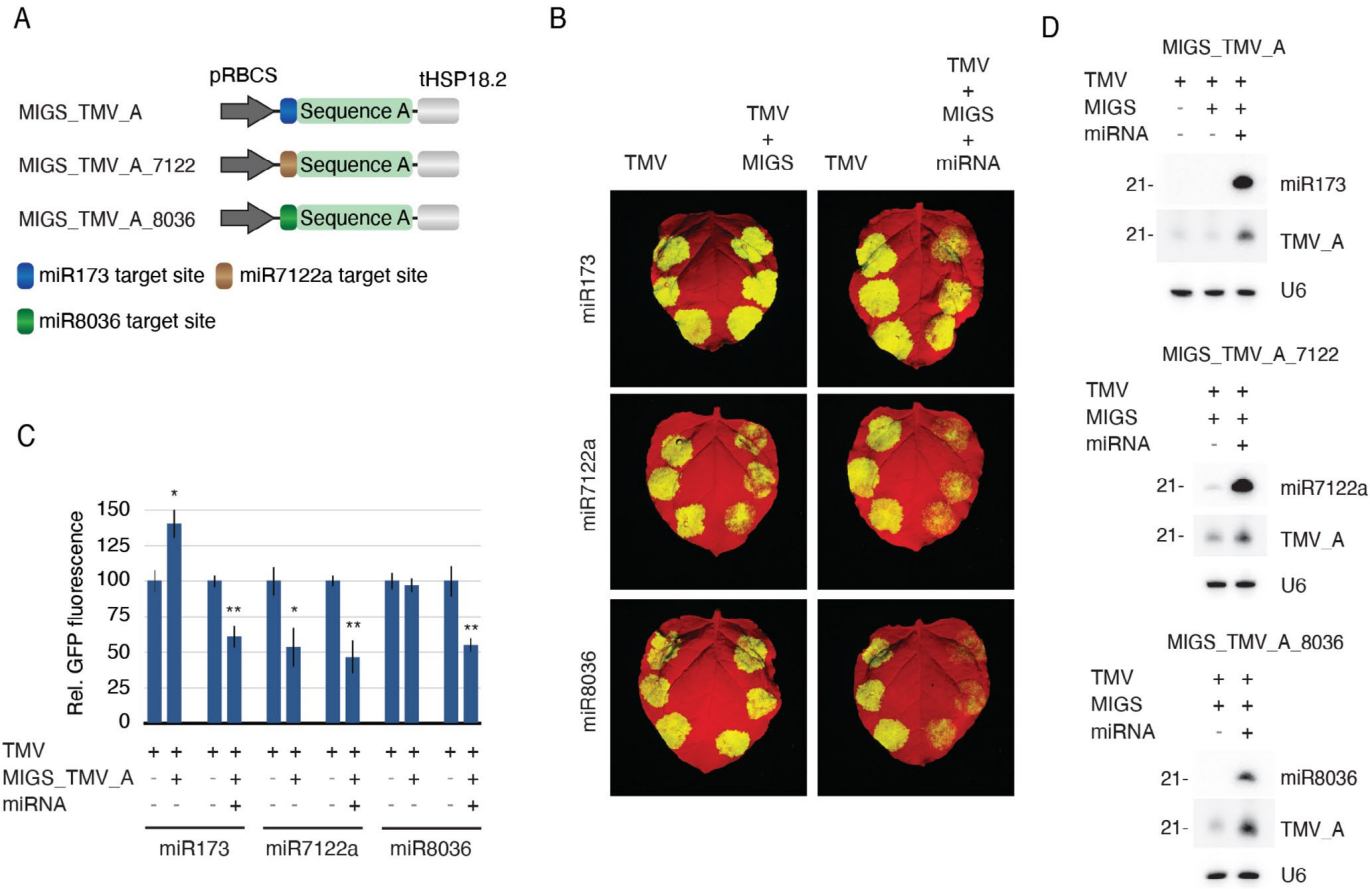
Validation of *N. benthamiana* miRNAs as MIGS initiators. (A) The MIGS_TMV_A construct was modified by replacing the miR173 binding sequence with the target site of either the miR7122a or miR8036 from *N. benthamiana*. (B) *N. benthamiana* leaves infiltrated with the virus alone (left side), in combination with the MIGS construct (right side, left panels) or co‐infiltrated with the MIGS and the miRNA (right side, right panels). (C) Average relative GFP fluorescence measured from six infiltration spots. Standard error bars and significant statistical differences are given (“*” and “**” refer to *p* ≤ 0.05 and 0.01, respectively). (D) migsiRNA northern blots showing the abundance of the respective miRNAs in the infiltrated leaves.

As a two‐component system, MIGS requires the concomitant expression of a miRNA capable of initiating secondary sRNA production (i.e., a MIGS initiator) and a transcript consisting of a sequence of the target gene plus the site recognised by this miRNA. Often, as in this work, the MIGS initiator used is the well‐characterised miR173 from *A. thaliana* . Nonetheless, miR173 is specific to Brassicaceae and needs to be co‐delivered with the MIGS construct when applied to plants from other families. For some applications (e.g., silencing via RNA spray), however, the co‐delivery of MIGS and its initiator can offer an extra level of difficulty, decreasing the appeal and even the efficiency of the technique. To overcome this limitation, MIGS can be adapted to rely on miRNAs present in the species of interest, provided they share miR173's ability to start secondary sRNA production. With this in mind, we screened for new MIGS initiators by finding 22 nt‐long miRNAs targeting genomic loci showing differential accumulation of RdR6‐derived sRNAs (Figure S9). Genomic loci of interest were assessed using PHASIS (Kakrana et al. 2017) to detect phasing patterns and to evaluate correlations between the miRNA cleavage site and phased sRNA generation. By applying this approach in *N. benthamiana*, we identified two miRNAs, miR7122a and miR8036, with the potential to trigger migsiRNA production. To validate these miRNAs as MIGS initiators, we generated pMIGS_TMV_A_7122 and pMIGS_TMV_A_8036, which are MIGS_TMV_A constructs where the miR173 binding site was replaced with the target sequence for either miR7122a or miR8036, respectively (Figure 6A). These constructs were co‐infiltrated with pTMV‐GFP into *N. benthamiana* in the presence or absence of the corresponding miRNA, and the effect on the virus expression was assessed. As expected, silencing of the virus with pMIGS_TMV_A only occurred when the miR173 was co‐expressed in the leaves (Figure 6B–D). In contrast, pMIGS_TMV_A_7122 did not require exogenous delivery of miR7122a for its activity, indicating that miR7122a is a *bona fide* MIGS initiator that is endogenously expressed at sufficient levels in the target tissue. Similarly, TMV silencing was achieved using pMIGS_TMV_A_8036, although in this case, endogenous levels of miR8036 were insufficient to trigger migsiRNA production. Consistently, miRNA8036 was only detected when provided as a transgene (Figure 6D). Together, these results confirm that both miR7122a and miR8036 can be used as MIGS initiators, with the former representing the most effective endogenous option for *N. bethamiana*.

## 3 Discussion

Simultaneous silencing of several genes is often difficult to achieve, yet it has broad potential applications in plant biology and biotechnology. Here, we demonstrate that many of the challenges of achieving multi‐gene silencing using approaches such as VIGS, hpRNAi, amiRNA can be overcome by using MIGS.

Multi‐gene silencing with a single amiRNA requires a conserved sequence among all target genes (Ossowski et al. 2008), which can make the downregulation of several targets a complicated task. As an alternative, polycistronic precursors can be used for the release of several 21 nt amiRNAs, each silencing one target or a group of transcripts sharing highly conserved sequences (Ai et al. 2011; Fahim et al. 2012; Kis et al. 2016; Kung et al. 2012; Miao et al. 2021; Park et al. 2009). This approach, however, involves the generation of complex molecules for which processing precision could vary intramolecularly and between different species (Carbonell et al. 2015; Lunardon et al. 2021). Moreover, amiRNA efficiency is highly susceptible to target accessibility, usually requiring testing of more than one molecule (Ossowski et al. 2008). In contrast, MIGS allows silencing of several unrelated genes by concatenating the appropriate MIGS modules into a single construct (de Felippes 2012; de Felippes et al. 2012). Similarly, both VIGS and hpRNAi can be adapted for multi‐gene silencing by fusing different gene sequences into a single CS. However, the size of the CS can be a limiting factor for both techniques. In the case of VIGS, silencing relies on the use of viral vectors, which often have limited cargo capacity, restricting the number of different genes that can be targeted simultaneously. The dependency on viral vectors also limits the range of plant hosts for which VIGS can be used and often results in symptoms of viral infection (Dommes et al. 2019; Watson et al. 2005; Zulfiqar et al. 2023). For hpRNAi, the need to create inverted repeats of the target sequence results in constructs that are twice as long as the MIGS modules. Such long hpRNAi constructs are prone to plasmid instability and have been associated with low plant transformation efficiency (Chung and Palukaitis 2011; Oliveira et al. 2009; Song et al. 2013). In addition, T‐DNA insertions containing hpRNAi constructs are susceptible to deletions affecting the inverted repeat region (Sunitha et al. 2012). In this study, we also observed uneven sRNA production from long hpRNAi molecules, with sRNA levels falling below detection in certain regions (Figure S6).

Multi‐gene silencing can also be achieved using alternative methods, such as synthetic trans‐acting small interfering RNAs (syn‐tasiRNAs, also known as artificial tasiRNA—atasiRNA) (Carbonell et al. 2014; Gutiérrez‐Nava et al. 2008). Like MIGS, this approach leverages the miRNA‐triggered biogenesis of secondary sRNAs. However, in the syn‐tasiRNA technology, a 21 nt synthetic sRNA designed to target the gene of interest replaces one of the predicted sRNAs (called tasiRNA) originating from the miRNA‐targeted transcript (known as *TAS*) (de Felippes 2019). Efficient silencing of four unrelated genes was achieved by using a single construct carrying four independent syn‐tasiRNAs (Carbonell et al. 2019). The number of targets could be potentially expanded by replacing additional tasiRNAs from the *TAS* precursor. It is not clear, however, if all the syn‐tasiRNAs would have the same silencing efficiency. According to deep‐sequencing data, tasiRNA production from the *TAS* precursor is not homogeneous, with some positions yielding abundant tasiRNAs, while in others, tasiRNAs are hardly detected (Allen et al. 2005; Axtell et al. 2006; de Felippes et al. 2017). As for amiRNAs, syn‐tasiRNA relies on a single designed 21 nt sRNA for gene silencing. While this can be advantageous for conferring target specificity (lower risk of off‐targets), it also makes silencing by syn‐tasiRNA more susceptible to target accessibility issues, which could reduce silencing efficiency (Ossowski et al. 2008). With MIGS, on the other hand, a population of migsiRNAs is produced from each module, improving the chances that one of the migsiRNAs will have a meaningful interaction with its target.

There are a few aspects that need to be considered when using MIGS. Like VIGS and hpRNAi, MIGS design is based on the use of a sequence fragment of the target and results in the production of a variable set of sRNAs. This characteristic could increase the chances of off‐targets, especially if the sequence used has a high degree of similarity to sequences in other genes. To minimise off‐targeting, it is advisable to select sequences with a low degree of conservation across the genome. In addition, software to estimate the likelihood of off‐target effects exists and can be used to minimise silencing of unintended targets (Naito et al. 2005; Xu et al. 2006). Off‐targeting might be less of a concern for targets with exogenous origin, such as viruses, due to the lower chance of sequence similarities between the plant host and the target. A second factor for consideration is the two‐component nature of MIGS. Silencing with MIGS requires the concomitant expression of the MIGS construct and the MIGS initiator. miR173 has been the MIGS initiator of choice for most MIGS applications; however, it is restricted to Brassicaceae. Therefore, the use of MIGS in species where miR173 is not present requires the miRNA to be co‐delivered with the MIGS construct or the identification of an alternative MIGS initiator that is properly expressed in the tissue where silencing is intended. Here, we presented a pipeline that can be adapted to identify new MIGS initiators in other species of interest (Figure S9). As shown in this work, the nucleotide composition of the target sequence can affect MIGS silencing efficiency and thus needs to be factored in when designing MIGS constructs. G/C‐rich sequences might increase the levels of migsiRNAs starting with a G in their 5′ end and making them less likely to interact with one of the AGOs involved in PTGS (Bologna and Voinnet 2014; Mi et al. 2008; Montgomery, Howell, et al. 2008; Takeda et al. 2008). Further discussion on the advantages and possible limitations when using different RNAi methods can be found elsewhere (de Felippes 2019; Pandey et al. 2015; Tiwari et al. 2014; Zulfiqar et al. 2023).

To date, MIGS has been used as a tool for gene silencing in several species, including important crop plants, such as tomato, rice, soybean (Jacobs et al. 2015; Singh et al. 2015; Zheng et al. 2018). In all these cases, MIGS applications relied on the use of transgenes, which have low acceptance in several countries, imposing limitations on the use of this technology for agronomical purposes. Non‐transgenic approaches for RNAi have emerged as an alternative to the challenges associated with genetically modified plants. Foliar spraying, infiltration, injection, spreading, mechanical inoculation, root/seed soaking have been widely used to deliver dsRNAs, siRNAs, hpRNAs, not only as biopesticides but also to regulate gene expression in plants (Das and Sherif 2020; Rank and Koch 2021; Septiani et al. 2025). Testing the feasibility of using such methods with MIGS is a necessary next step to unleash the full potential of MIGS as a biotechnological tool.

Unexpectedly, we observed a decline in migsiRNAs being produced from multi‐modular MIGS constructs, especially from module 5 onwards. This could be the result of the repetitive nature of miR173 target sites in constructs containing several modules. Direct repeats are a known source of plasmid instability (Oliveira et al. 2009), and having the same sequence repeated several times in the same molecule could cause plasmid rearrangements that affect the production of migsiRNAs from long MIGS constructs. We, however, did not observe any rearrangements while generating the MIGS constructs. In addition, the pattern in migsiRNA accumulation, with migsiRNA levels declining as we move towards the 3′ end of the molecule, suggests another possibility. Hidden transcription stop sites could be present in the MIGS construct, leading to early transcription termination and a fraction of the transcripts being truncated. In this scenario, a significant proportion of the MIGS transcripts would not carry the sequence of downstream modules, affecting the levels of migsiRNA originating from those regions. Although we did not test for the presence of truncated transcripts or other signs of early termination, it is possible to find in the sequence of MIGS_15X (Data S1) several short sequences identical to the most common polyadenylation signals (PAS) in *A. thaliana* (Loke et al. 2005). A functional PAS is one of the factors that can induce transcription termination (de Felippes and Waterhouse 2022; Porrua et al. 2016; Proudfoot 2016), and the presence of such sites in the middle of the MIGS construct could be causing early transcription termination. If this is the case, using sequences from the gene coding region (instead of random sequences) to generate MIGS modules might decrease the chances that a functional PAS is present. Nevertheless, despite reduced levels, migsiRNAs originating from downstream modules were abundant and induced significant reductions in gene expression levels (Figure 3D,E). Full silencing would still be expected for targets with low expression, and, in some cases, a milder silencing could even be advantageous, especially for situations where gene knockout has deleterious effects on the plant.

In this work, the usefulness of multi‐gene silencing for plant protection was illustrated by the stronger efficiency of a single MIGS construct targeting three different TMV genomic regions for silencing. According to our data (Figures 1 and S1), the better efficiency of MIGS_TMV_ABC over the other single‐ and double‐module constructs was mostly due to an increase in the total amount of migsiRNAs targeting the virus. However, the fact that MIGS_TMV_ABC had a greater impact on the virus expression than MIGS_TMV_AAA suggests that some of the increased efficiency of the former construct resulted from targeting multiple viral regions for silencing. Indeed, targeting distinct regions within the virus genome can have diverse effects on virus suppression. Notably, sRNAs directed to viral suppressors of RNA silencing were shown to be more effective than those targeting capsid proteins and other viral genes (Vu et al. 2013). Even targeting different regions within the same gene can result in variations in silencing levels. For instance, hairpins generated using the first 200 bp of the potato virus Y (PVY) coat protein (CP) had poor performance compared to similar molecules carrying sequences originating from the 3′ end of the same gene (Jiang et al. 2011). Multi‐gene silencing is also a valuable tool for extending the protection provided by sRNAs. Silencing relies on the complementarity of the sRNA with its target. Viruses can eventually evade this protection by mutating their genome at the sRNA target site, preventing recognition and/or cleavage by RISC (Lafforgue et al. 2011). Targeting the virus in more than one location can prevent such mutants from emerging, since genomic changes would have to happen in several regions simultaneously for the virus to escape silencing. Thus, multi‐gene silencing can make resistance‐breaking events less likely and extend the lifespan of sRNA‐mediated protection (Carbonell et al. 2019; Lafforgue et al. 2013).

In summary, our results support MIGS as a versatile and efficient tool for multi‐gene silencing, offering an efficient and straightforward option for downregulating the expression of multiple genes. Several targets can be silenced by simply arranging the respective MIGS modules in one expression cassette in tandem. The number of targets could be easily duplicated by generating modules carrying CS made with the sequences of different targets instead of just one.

## 4 Methods

### 4.1 Plant Material and Transformation

*A. thaliana* (Col‐0) and *N. benthamiana* plants were grown under a 16:8 h light/dark photocycle at 21°C–23°C and 25°C, respectively. The only exception was for the experiments investigating the silencing of *ELF3*, where *A. thaliana* plants were grown under short‐day conditions (8:16 h light/dark).

Transgenic *A. thaliana* plants were generated by *Agrobacterium tumefaciens* ‐mediated floral dip (Clough and Bent 1998). Transformed plants were selected by germinating seeds on soil soaked with BASTA (Bayer, Leverkusen, Germany). Agroinfiltration of *N. benthamiana* leaves was done on 4‐week‐old plants as previously described (de Felippes and Weigel 2010) and analysed 3 days post‐infiltration. For each construct, three to five plants were used, with infiltrations done in two leaves per plant, three spots per leaf.

### 4.2 Molecular Constructs

All MIGS sequences were cloned into the binary plasmids pRBCS::GiFiP::tHSP or p35S::GiFiP::tHSP (de Felippes et al. 2020) by replacing the *GiFiP* in these vectors using XmaI and NotI sites. For single‐module MIGS constructs, the sequence of interest was PCR amplified using specific primers containing the recognition site of XmaI (forward primer) or NotI (reverse primer). In addition, the forward primer also contained the binding site recognised by miR173 or one of the *N. benthamiana* miRNAs for incorporation of the miRNA target site upstream of the sequence of interest. MIGS constructs carrying two, three, four modules targeting TMV and/or PVX were created by fusing the respective single modules into a CS using overlapping PCR. For constructs testing MIGS range (pMIGS_3X to pMIGS_15X), CSs containing three modules were synthesised as gBlocks (Integrate DNA Technologies) and sequentially cloned using restriction enzymes. To add the TMV_A module in those constructs, the MIGS sequence was PCR amplified using specific primers containing sites of restriction enzymes of choice and cloned into the respective plasmids. pMIGS_TMV_A_(U) and pMIGS_TMV_A_(G) were created using gBlocks carrying the modified MIGS_TMV_A sequence flanked by the XmaI and NotI sites. The precursor molecules of miR173, miR7122, miR8036 were PCR amplified from genomic DNA using primers containing XmaI and NotI sites (forward and reverse, respectively) and inserted into p35S::GiFiP::tRBCS, replacing the *GiFiP* gene. To generate the binary vector containing the MIGS_TMV_A and the miR173 precursor, the miR173 expression cassette was PCR amplified and inserted into pMIGS_TMV_A cut with XhoI, using NEBuilder HiFi DNA Assembly (New England Biolabs). The sequences of all MIGS modules and primers used for the constructions are given in Data S1 and Table S2.

### 4.3 GFP Analysis

GFP fluorescence was visualised using a combination of blue light (Dark Reader Hand Lamp HL32T, Clare Chemical Research, Dolores, USA) and an orange filter for the experiments involving TMV‐GFP and UV light for PVX‐GFP. Pictures were taken with a Canon EOS550D camera with settings adjusted to each experiment as required. For TMV‐GFP images, the camera was fitted with an orange G filter (Orange G HMC filter; HOYA filters, Tokyo, Japan). For the quantification of GFP fluorescence induced by blue light, tissue (6–12 infiltration spots) was collected using a cork borer (8 mm diameter) for input normalisation, frozen in liquid nitrogen, and ground using a TissueLyser II (Qiagen, Hilden, Germany). The tissue was resuspended in 150 μL of lysis buffer (50 mM Tris–HCl, pH 7.5, 150 mM NaCl, 1 mM EDTA and 1% Nonidet P‐40) and 10 μL of the sample was further diluted in 90 μL of lysis buffer. GFP fluorescence measurements were done with the Glomax R Discover (Promega, Madison, USA) using the GFP quantification protocol (excitation: 475 nm, emission: 500–550 nm). For UV‐induced GFP, fluorescence levels were quantified by measuring the grey value (i.e., the intensity of a pixel in a grayscale image) of individual infiltration spots using FIJI (http://fiji.sc) (Schindelin et al. 2012). After conversion of images to 16‐bit, a circular region of interest (ROI) was created (width = 36, height = 36, area = 1020) and used to obtain the mean grey value employing the measure tool. For each agroinfiltrated spot, the grey value was the average of three independent measurements. To compensate for variations existing in the different images (due to differing infiltration efficiency, shapes of the leaves, etc.), the grey value of each inoculation spot was normalised to the reference construct (pPVX‐GFP) immediately next to it.

### 4.4 RNA Extraction and sRNA Northern Blot

Total RNA was extracted using TRIzol reagent (Thermo Fisher Scientific, Waltham, USA) according to the manufacturer's instructions. sRNAs Northern blots were performed using one to five micrograms of total RNA separated by electrophoresis in a 17% polyacrylamide gel, containing 7 M urea. After transfer to a positively charged membrane, sRNAs were fixed by UV light and hybridised with specific probes (Table S2). For radioactive detection, probes were labelled with α‐^32^P‐dCTP using the terminal deoxyribonucleotidyl transferase (Fermentas, Thermo Fisher Scientific, Waltham, USA) for oligonucleotide DNA probes and the Prime‐a‐Gene Labelling System (Promega, Madison, USA) for random‐labelled probes. Non‐radioactive detection was done with specific probes generated by PCR using the DIG DNA Labelling Mix (Roche, Sigma‐Aldrich, St Louis, USA). After hybridisation, membranes were incubated with the Anti‐Digoxigenin‐AP (Roche, Sigma‐Aldrich, St Louis, USA) and the signal was detected with the CDP‐Star substrate (Roche, Sigma‐Aldrich, St Louis, USA). sRNA blots were performed at least twice using different biological replicates.

### 4.5 Virus Load Quantification

cDNA used to detect virus load in infected samples was prepared using one microgram of total RNA and the RevertAid RT Reverse Transcription kit (Thermo Fisher Scientific, Waltham, USA) with Random Hexamer Primers. The RT‐qPCR reaction was prepared using GoTaq R qPCR Master Mix (Promega, Madison, USA), 10 ng of cDNA and 10 pmol of primers (final volume of 10 μL). PCR was performed on an Mx3000P machine (Stratagene, San Diego, USA); PCR conditions were 95°C for 10 min, followed by 40 cycles of 95°C for 30 s, 60°C for 1 min, 72°C for 1 min. Melting curve analysis was done by heating samples from 65°C to 95°C with increments of 0.5°C for 5 s. Three technical replicates per sample and three biological replicates were used.

### 4.6 sRNA Sequencing and Bioinformatic Analysis

Library preparation and sRNA‐Seq data were generated at the Ramaciotti Centre for Genomics (UNSW Sydney, Australia). Libraries were prepared using the QIASeq miRNA library kit (Qiagen, Hilden, Germany) and sequenced on a NovaSeq 6000 (Illumina, San Diego, USA) using an SP flow cell (100 cycles) for the pMIGS_15X (1 replicate) and on a NovaSeq X Plus using a 10B flow cell for the pMIGS_TMV_A, pMIGS_TMV_A_(U), pMIGS_TMV_A_(G) (3 replicates each). Initial quality checks were done with VirReport v3 (Gauthier et al. 2022) as follows: (i) reads were filtered for any residual sequencing adapters using Cutadapt 3.5 (Martin 2011), and UMIs were then extracted from the reads using umi‐tools 1.1.2 (Smith et al. 2017); (ii) reads were then subjected to quality filtering with a Q score threshold of 30 and an unknown base limit of zero using Cutadapt 3.5.3; (iii) the quality of reads was evaluated based on the total number of reads left over after quality filtering, the sample read length distribution profile, FastQC output, the quantity of non‐informative content (e.g., ribosomal RNA) recovered. Quality‐filtered reads that were 21 and 22 nt in length were mapped to the nucleotide sequences of the MIGS constructs using Bowtie 1.3.0 (Langmead et al. 2009), and BAM files were derived using Samtools 1.12 (Li et al. 2009). Reads were deduplicated using umi_tools 1.1.2, and coverage statistics were derived using GATK 4.5.0 (McKenna et al. 2010). Total read counts were normalised to reads per million mapped reads (i.e., RPM). The data presented in this study are openly available in the short read archive (SRA) database under the BioProject PRJNA1250781.

### 4.7 Identification of MIGS Endogenous Initiators

We leveraged small RNA‐seq data from wild‐type and *rdr6* mutant *N. benthamiana* plants collected to identify genomic loci affected by the loss of RDR6 function (SRA database available under the BioProject PRJNA1268705). Briefly, raw FASTQ files were quality‐trimmed using TrimGalore (Krueger et al. 2023). High‐quality reads were then mapped onto 500 bp‐long sliding genomic windows derived from the *N. benthamiana* genome (Ranawaka et al. 2023) using bowtie, allowing no mismatches and up to 10 mapping locations (Langmead et al. 2009). Feature counts for each window were estimated using samtools‐idxstats (Li et al. 2009). Differential expression analysis was performed using DESeq2 with an FDR threshold of < 0.05 (Love et al. 2014). The presence of phased siRNA clusters within significantly differentially expressed windows, and candidate miRNA initiators, was assessed using PHASIS (Kakrana et al. 2017). psRNATarget (Dai et al. 2018) was then used to predict downstream targets for 22 nt‐long miRNAs. Identified targets were selected and sRNA libraries mapped onto gene sequences to inspect the phasing of the miRNA‐induced cleavage.

## Conflicts of Interest

The authors declare no conflicts of interest.

## Supporting information


**Figure S1:** Comparison between 3‐module MIGS constructs targeting three distinct genes versus three times the same gene.
**Figure S2:** Phasing analysis of migsiRNAs produced from pMIGS_15X.
**Figure S3:** Size distribution of migsiRNAs mapping to the different modules of pMIGS_15X.
**Figure S4:** Analysis of phasing in migsiRNAs originating from pMIGS_6X and pMIGS_9X.
**Figure S5:** MigsiRNA size distribution in the different modules of pMIGS_6X and pMIGS_9X.
**Figure S6:** Testing the effect of the inverted repeat size in the sRNA production from hpRNAi constructs.
**Figure S7:** Effect of target sequence size on MIGS efficiency using *AG* as a reporter gene.
**Figure S8:** Size distribution (A) and phasing analyses (B) of sRNAs mapping to pMIGS_TMV_A, pMIGS_TMV_A_(U) and pMIGS_TMV_A_(G).
**Figure S9:** Identification of endogenous *N. benthamiana* MIGS initiators.

**Data S1:** pbi70401‐sup‐0002‐Supinfo.docx.

**Table S1:** Mean read count of 21 and 22 nt sRNA reads against the pMIGS_15X construct. Mean read count for interval regions of interest, calculated using GATK.

**Table S2:** List of primers and probes used in this work. Restriction enzyme sites are shown underlined and the target sequence for the MIGS initiator is in bold (miR173 unless otherwise indicated).

## Data Availability

The data that support the findings of this study are openly available in Biosample at https://www.ncbi.nlm.nih.gov/biosample, reference number SUB15573089, which will be made available upon publication.
