# Supplementary material for: MicroRNA‐Induced Gene Silencing (MIGS): A Tool for Multi‐Gene Silencing and Targeting Viruses in Plants
Source: Plant Biotechnol J. 2025 Oct 6;24(3):973–87. doi: 10.1111/pbi.70401 (PMC12946496; doi:10.1111/pbi.70401)
Supplement: Supplementary file 1 — Figure S1: Comparison between 3‐module MIGS constructs targeting three distinct genes versus three times the same gene. Figure S2: Phasing analysis of migsiRNAs produced from pMIGS_15X. Figure S3: Size distribution of migsiRNAs mapping to the different modules of pMIGS_15X. Figure S4: Analysis of phasing in migsiRNAs originating from pMIGS_6X and pMIGS_9X. Figure S5: MigsiRNA size distribution in the different modules of pMIGS_6X and pMIGS_9X. Figure S6: Testing the effect of the inverted repeat size in the sRNA production from hpRNAi constructs. Figure S7: Effect of target sequence size on MIGS efficiency using AG as a reporter gene. Figure S8: Size distribution (A) and phasing analyses (B) of sRNAs mapping to pMIGS_TMV_A, pMIGS_TMV_A_(U) and pMIGS_TMV_A_(G). Figure S9: Identification of endogenous N. benthamiana MIGS initiators. [file PBI-24-973-s003.docx]

Supplementary figures for

**MicroRNA-induced gene silencing (MIGS): a tool for multi-gene silencing and defending plants against viruses**

Marie-Emilie A. Gauthier*, Kylie Shand*, Satomi Hayashi, Peter M. Waterhouse, Roberto A. Barrero and Felipe F. de Felippes


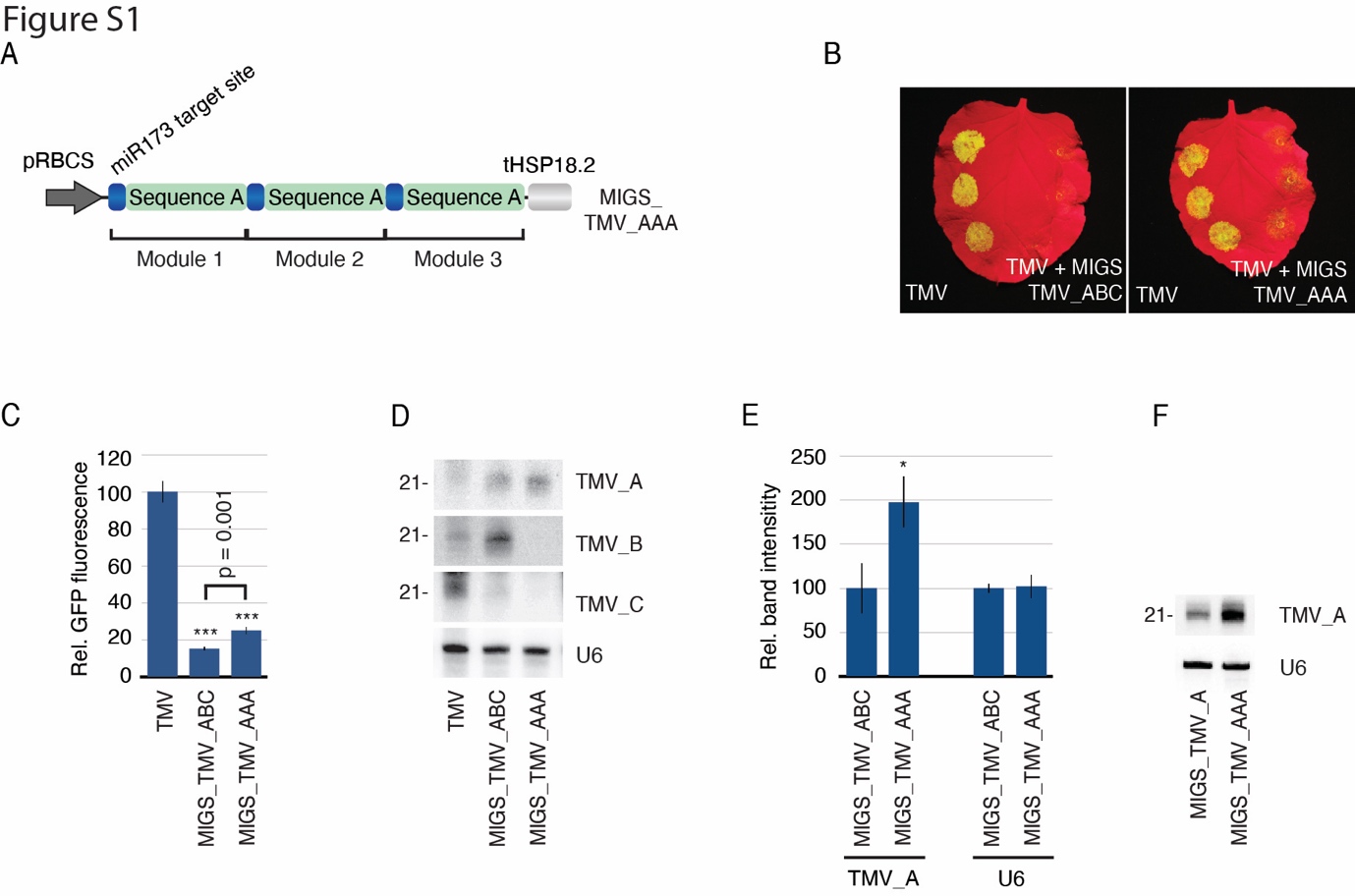


**Figure S1.** Comparison between 3-module MIGS constructs targeting three distinct genes versus three times the same gene. **(A)** Representation of the MIGS construct carrying three modules, all targeting the TMV Rep gene. **(B)** *N. benthamiana* leaves agroinfiltrated with the TMV virus alone (left) and the virus plus one of the MIGS construct and miR173 (right). **(C)** Relative GFP fluorescence for 12 infiltration spots is shown. **(D)** migsiRNA accumulation was detected using a northern blot, with U6 as a loading reference. **(E)** Relative intensity of bands detected in three independent northern blots probed with the TMV_A probe. The same analysis for bands detected when using the U6 probe is shown for comparison. **(F)** Northern blot showing the sRNA accumulation detected in *N. benthamiana* leaves agroinfiltrated with either the MIGS_TMV_A or MIGS_TMV_AAA construct. Statistical significances for the difference seen between constructs was calculated using the Mann-Whitney U test.

**Figure S2.** Phasing analysis of migsiRNAs produced from pMIGS_15X. Radar plots show percentages of reads corresponding to each of the 21 registers. Position 1 corresponds to the register set by the miR173 cleavage.

**Figure S3.** Size distribution of migsiRNAs mapping to the different modules of pMIGS_15X.

**Figure S4.** Analysis of phasing in migsiRNAs originating from pMIGS_6X and pMIGS_9X. The percentage of migsiRNA produced in each of the different registers is shown. Position 1 corresponds to the register set by the miR173 cleavage.

**Figure S5.** MigsiRNA size distribution in the different modules of pMIGS_6X and pMIGS_9X.


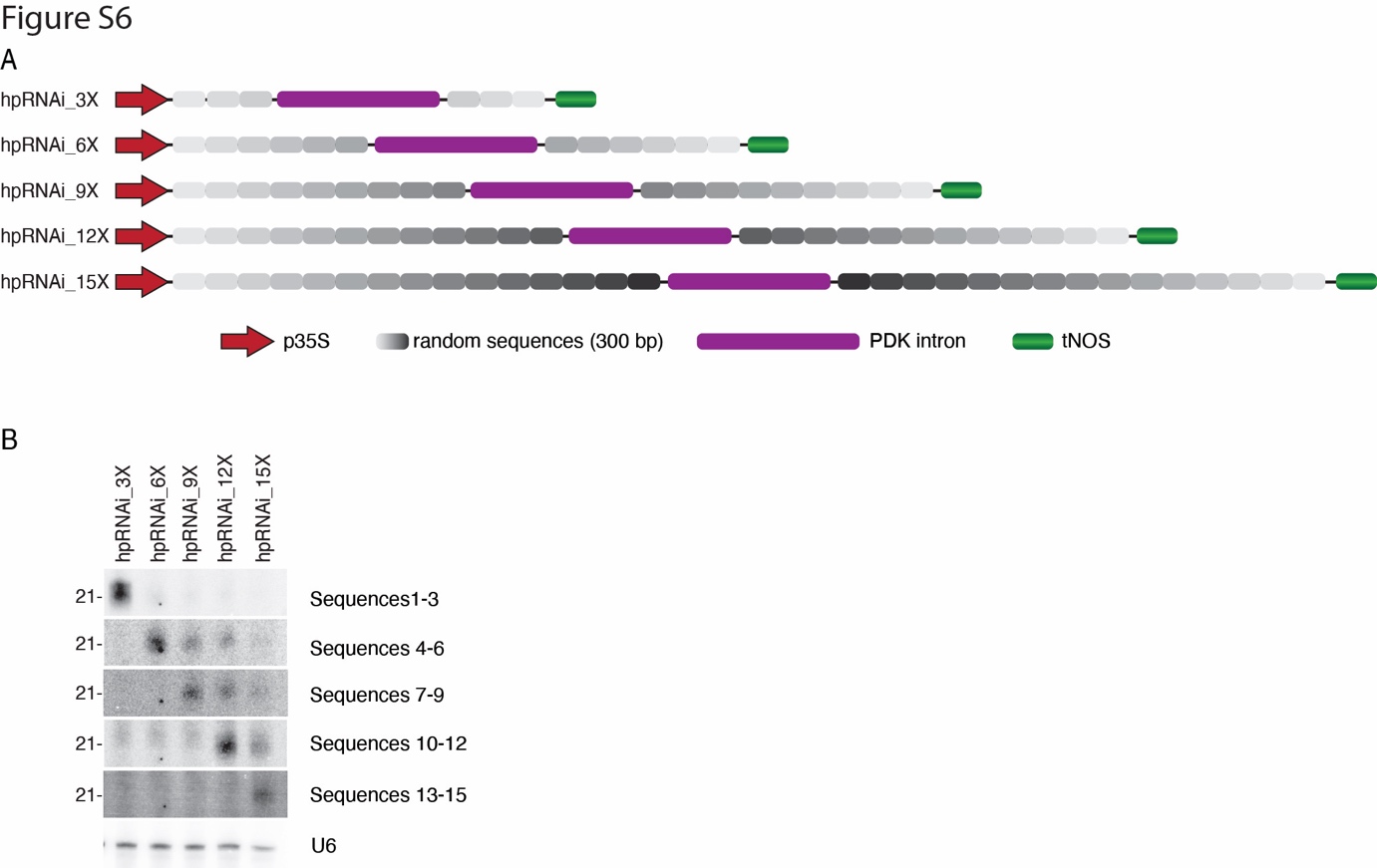


**Figure S6.** Testing the effect of the inverted repeat size in the sRNA production from hpRNAi constructs. **(A)** hpRNAi constructs driven by the CaMV 35S promoter (p35S) and the NOS terminator (tNOS). The fragment used to generate the inverted repeat was created by the fusion of different numbers of random sequences. **(B)** Northern blots showing the accumulation of sRNAs from different regions of the hpRNAi construct. Probes were designed to detect sRNAs originating from a group of three fragments. U6 is used as a loading control.


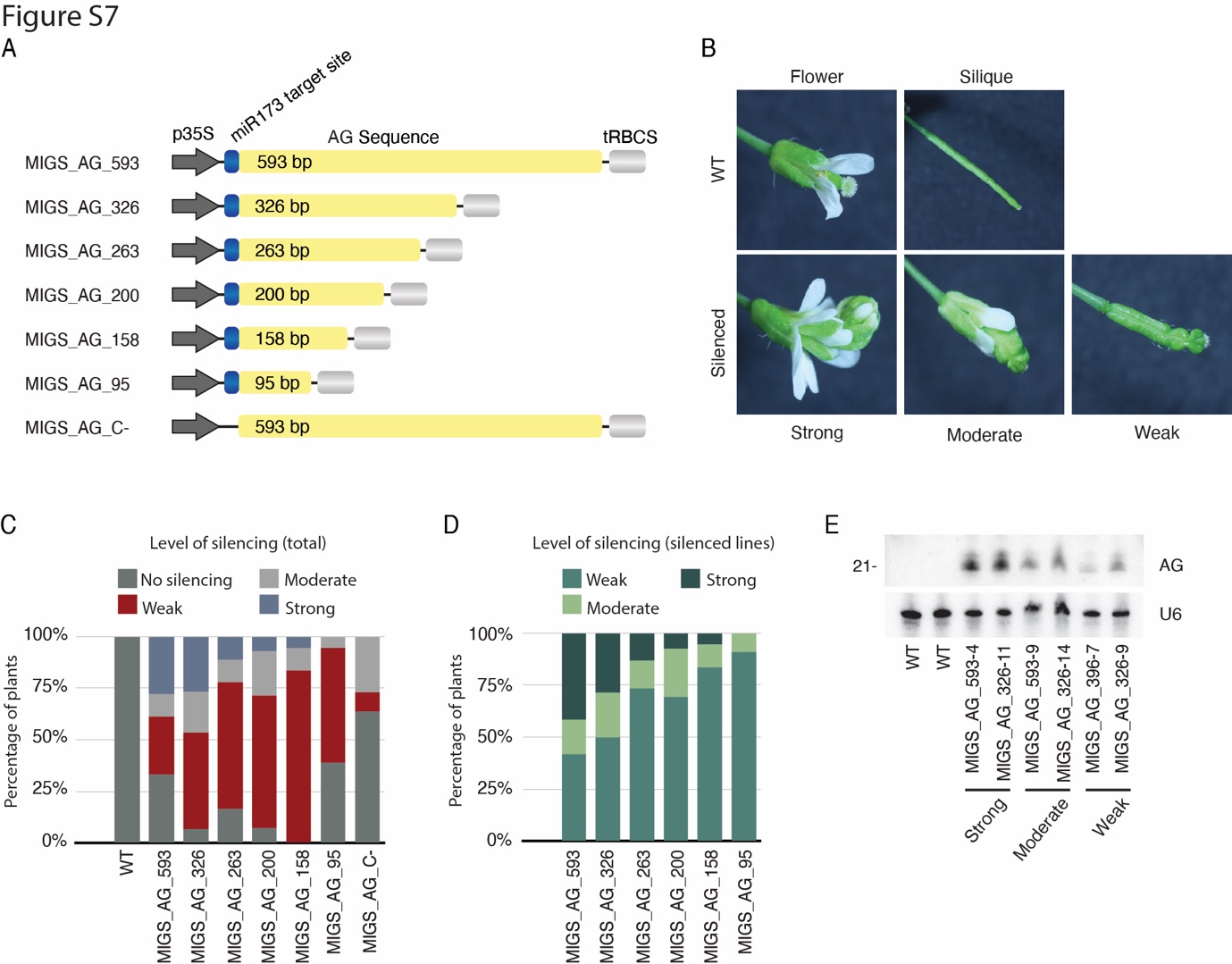


**Figure S7.** Effect of target sequence size on MIGS efficiency using *AG* as a reporter gene. **(A)** MIGS constructs to silence *AG* containing different sequence sizes of the target. **(B)** Representative phenotype of wild-type (WT) flowers and silique, as well as the phenotype for the degrees of silencing observed. **(C)** The percentage of plants showing flower defects categorised by the silencing intensity (at least 17 independent plants). **(D)** Proportion of each degree of silencing among plants showing flower developmental defects. **(E)** Northern blot analysis of migsiRNAs originating from the MIGS constructs. U6 is used as a loading control.

**Figure S8.** Size distribution **(A)** and phasing analyses **(B)** of sRNAs mapping to pMIGS_TMV_A, pMIGS_TMV_A_(U) and pMIGS_TMV_A_(G). The register set by the miR173 cleavage is represented by position 1.


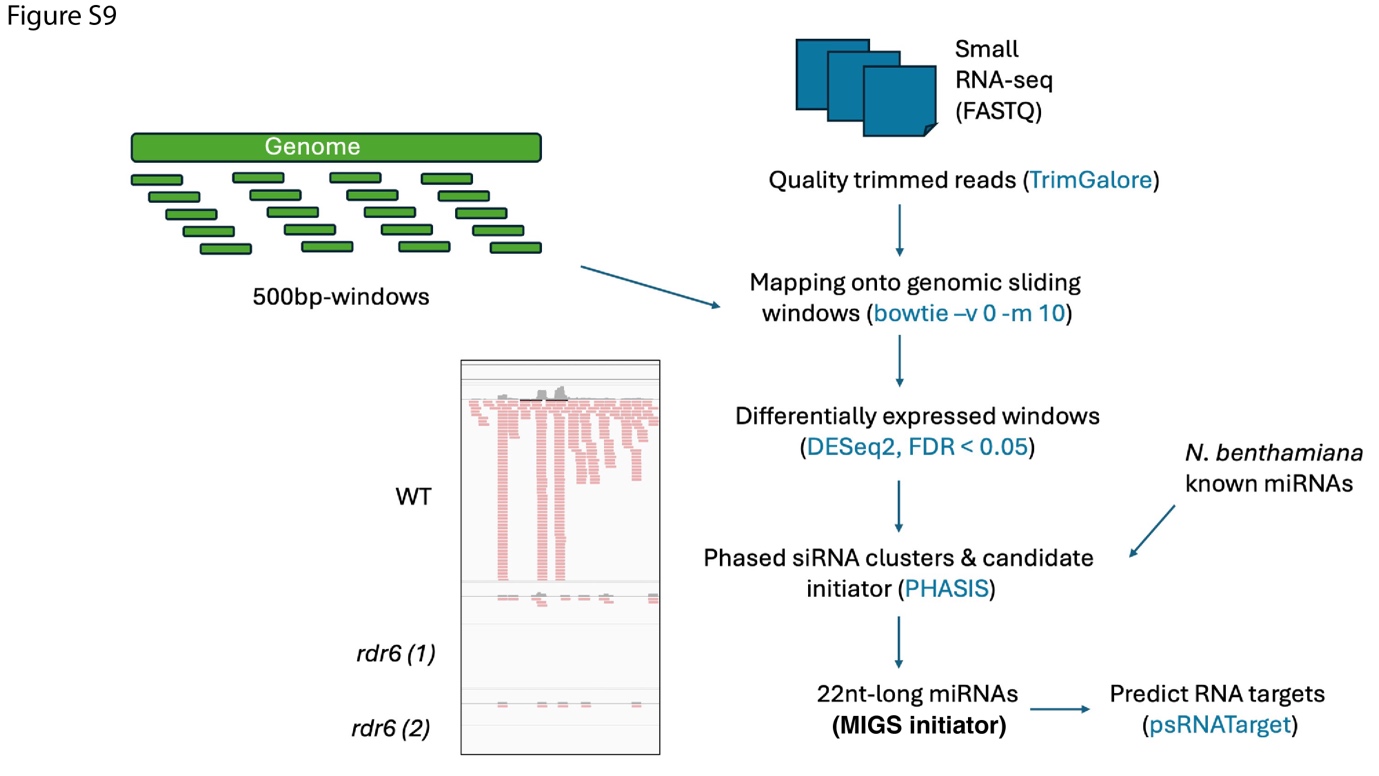


**Figure S9.** Identification of endogenous *N. benthamiana* MIGS initiators. Quality trimmed small RNAs derived were mapped onto overlapping sliding genomic windows (window size = 500nt; step = 100 nt). Genomics windows with differential mapping of small RNAs were identified using DESeq2 (FDR < 0.05). Phased siRNA clusters and predicted candidate MIG initiators identified using PHASIS. Targets for 22 nt-long miRNAs were predicted using psRNATarget against annotated *N. benthamiana* transcriptome.
