# Supplementary material for: MicroRNA‐Induced Gene Silencing (MIGS): A Tool for Multi‐Gene Silencing and Targeting Viruses in Plants
Source: Plant Biotechnol J. 2025 Oct 6;24(3):973–87. doi: 10.1111/pbi.70401 (PMC12946496; doi:10.1111/pbi.70401)
Supplement: Supplementary file 2 — Data S1: pbi70401‐sup‐0002‐Supinfo.docx. [file PBI-24-973-s004.docx]

Supporting Information for

**MicroRNA-induced gene silencing (MIGS): a tool for multi-gene silencing and defending plants against viruses**

Marie-Emilie A. Gauthier*, Kylie Shand*, Satomi Hayashi, Peter M. Waterhouse, Roberto A. Barrero and Felipe F. de Felippes

1) The sequence of the different MIGS constructs used in the present work is given bellow. The target site for the MIGS initiator (miR173, miR7122 and miR8036) is shown in **bold**.

**MIGS_TMV_A**

**GTGATTTTTCTCTACAAGCGAA**TGCCGTGCATTCGCTTGCAGGTGGATTGCGATCTTTAGAACTGGAATATCTGATGATGCAAATTCCCTACGGATCATTGACTTATGACATAGGCGGGAATTTTGCATCGCATCTGTTCAAGGGACGAGCATATGTACACTGCTGCATGCCCAACCTGGACGTTCGAGACATCATGCGGCACGAAGGCCAGAAAGACAGTATTGAACTATACCTTTCTAGGCTAGAGAGAGGGGGGAAAACAGTCCCCAACTTCCAAAAGGAAGCATTTGACAGATACGCAGAAATTCCTGAAGACGCTGTCTGTCACAATACTTTCCAGACATGCG

**MIGS_TMV_B**

**GTGATTTTTCTCTACAAGCGAA**CAGTTTTACTATGATAAGTGTCTCCCAGGCAACAGCACCATGATGAATAATTTTGATGCTGTTACCATGAGGTTGACTGACATTTCATTGAATGTCAAAGATTGCATATTGGATATGTCTAAGTCTGTTGCTGCGCCTAAGGATCAAATCAAACCACTAATACCTATGGTACGAACGGCGGCAGAAATGCCACGCCAGACTGGACTATTGGAAAATTTAGTGGCGATGATTAAAAGAAACTTTAACGCACCCGAGTTGTCTGGCATCATTGATATTGAAAATACTGCATCTTTGGTTGTAGATAAGTTTTTTGATAGTTATTTGCT

**MIGS_TMV_C**

**GTGATTTTTCTCTACAAGCGAA**GCTCTAGTTGTTAAAGGAAAAGTGAATATCAATGAGTTTATCGACCTGACAAAAATGGAGAAGATCTTACCGTCGATGTTTACCCCTGTAAAGAGTGTTATGTGTTCCAAAGTTGATAAAATAATGGTTCATGAGAATGAGTCATTGTCAGGGGTGAACCTTCTTAAAGGAGTTAAGCTTATTGATAGTGGATACGTCTGTTTAGCCGGTTTGGTCGTCACGGGCGAGTGGAACTTGCCTGACAATTGCAGAGGAGGTGTGAGCGTGTGTCTGGTGGACAAAAGGATGGAAAGAGCCGACGAGGCCACTCTCGGATCTTACTACAC

**MIGS_TMV_AB**

**GTGATTTTTCTCTACAAGCGAA**TGCCGTGCATTCGCTTGCAGGTGGATTGCGATCTTTAGAACTGGAATATCTGATGATGCAAATTCCCTACGGATCATTGACTTATGACATAGGCGGGAATTTTGCATCGCATCTGTTCAAGGGACGAGCATATGTACACTGCTGCATGCCCAACCTGGACGTTCGAGACATCATGCGGCACGAAGGCCAGAAAGACAGTATTGAACTATACCTTTCTAGGCTAGAGAGAGGGGGGAAAACAGTCCCCAACTTCCAAAAGGAAGCATTTGACAGATACGCAGAAATTCCTGAAGACGCTGTCTGTCACAATACTTTCCAGACATGCG**GTGATTTTTCTCTACAAGCGAA**CAGTTTTACTATGATAAGTGTCTCCCAGGCAACAGCACCATGATGAATAATTTTGATGCTGTTACCATGAGGTTGACTGACATTTCATTGAATGTCAAAGATTGCATATTGGATATGTCTAAGTCTGTTGCTGCGCCTAAGGATCAAATCAAACCACTAATACCTATGGTACGAACGGCGGCAGAAATGCCACGCCAGACTGGACTATTGGAAAATTTAGTGGCGATGATTAAAAGAAACTTTAACGCACCCGAGTTGTCTGGCATCATTGATATTGAAAATACTGCATCTTTGGTTGTAGATAAGTTTTTTGATAGTTATTTGCT

**MIGS_TMV_AC**

**GTGATTTTTCTCTACAAGCGAA**TGCCGTGCATTCGCTTGCAGGTGGATTGCGATCTTTAGAACTGGAATATCTGATGATGCAAATTCCCTACGGATCATTGACTTATGACATAGGCGGGAATTTTGCATCGCATCTGTTCAAGGGACGAGCATATGTACACTGCTGCATGCCCAACCTGGACGTTCGAGACATCATGCGGCACGAAGGCCAGAAAGACAGTATTGAACTATACCTTTCTAGGCTAGAGAGAGGGGGGAAAACAGTCCCCAACTTCCAAAAGGAAGCATTTGACAGATACGCAGAAATTCCTGAAGACGCTGTCTGTCACAATACTTTCCAGACATGCG**GTGATTTTTCTCTACAAGCGAA**GCTCTAGTTGTTAAAGGAAAAGTGAATATCAATGAGTTTATCGACCTGACAAAAATGGAGAAGATCTTACCGTCGATGTTTACCCCTGTAAAGAGTGTTATGTGTTCCAAAGTTGATAAAATAATGGTTCATGAGAATGAGTCATTGTCAGGGGTGAACCTTCTTAAAGGAGTTAAGCTTATTGATAGTGGATACGTCTGTTTAGCCGGTTTGGTCGTCACGGGCGAGTGGAACTTGCCTGACAATTGCAGAGGAGGTGTGAGCGTGTGTCTGGTGGACAAAAGGATGGAAAGAGCCGACGAGGCCACTCTCGGATCTTACTACAC

**MIGS_TMV_BC**

**GTGATTTTTCTCTACAAGCGAA**CAGTTTTACTATGATAAGTGTCTCCCAGGCAACAGCACCATGATGAATAATTTTGATGCTGTTACCATGAGGTTGACTGACATTTCATTGAATGTCAAAGATTGCATATTGGATATGTCTAAGTCTGTTGCTGCGCCTAAGGATCAAATCAAACCACTAATACCTATGGTACGAACGGCGGCAGAAATGCCACGCCAGACTGGACTATTGGAAAATTTAGTGGCGATGATTAAAAGAAACTTTAACGCACCCGAGTTGTCTGGCATCATTGATATTGAAAATACTGCATCTTTGGTTGTAGATAAGTTTTTTGATAGTTATTTGCT**GTGATTTTTCTCTACAAGCGAA**GCTCTAGTTGTTAAAGGAAAAGTGAATATCAATGAGTTTATCGACCTGACAAAAATGGAGAAGATCTTACCGTCGATGTTTACCCCTGTAAAGAGTGTTATGTGTTCCAAAGTTGATAAAATAATGGTTCATGAGAATGAGTCATTGTCAGGGGTGAACCTTCTTAAAGGAGTTAAGCTTATTGATAGTGGATACGTCTGTTTAGCCGGTTTGGTCGTCACGGGCGAGTGGAACTTGCCTGACAATTGCAGAGGAGGTGTGAGCGTGTGTCTGGTGGACAAAAGGATGGAAAGAGCCGACGAGGCCACTCTCGGATCTTACTACAC

**MIGS_TMV_ABC**

**GTGATTTTTCTCTACAAGCGAA**TGCCGTGCATTCGCTTGCAGGTGGATTGCGATCTTTAGAACTGGAATATCTGATGATGCAAATTCCCTACGGATCATTGACTTATGACATAGGCGGGAATTTTGCATCGCATCTGTTCAAGGGACGAGCATATGTACACTGCTGCATGCCCAACCTGGACGTTCGAGACATCATGCGGCACGAAGGCCAGAAAGACAGTATTGAACTATACCTTTCTAGGCTAGAGAGAGGGGGGAAAACAGTCCCCAACTTCCAAAAGGAAGCATTTGACAGATACGCAGAAATTCCTGAAGACGCTGTCTGTCACAATACTTTCCAGACATGCG**GTGATTTTTCTCTACAAGCGAA**CAGTTTTACTATGATAAGTGTCTCCCAGGCAACAGCACCATGATGAATAATTTTGATGCTGTTACCATGAGGTTGACTGACATTTCATTGAATGTCAAAGATTGCATATTGGATATGTCTAAGTCTGTTGCTGCGCCTAAGGATCAAATCAAACCACTAATACCTATGGTACGAACGGCGGCAGAAATGCCACGCCAGACTGGACTATTGGAAAATTTAGTGGCGATGATTAAAAGAAACTTTAACGCACCCGAGTTGTCTGGCATCATTGATATTGAAAATACTGCATCTTTGGTTGTAGATAAGTTTTTTGATAGTTATTTGCT**GTGATTTTTCTCTACAAGCGAA**GCTCTAGTTGTTAAAGGAAAAGTGAATATCAATGAGTTTATCGACCTGACAAAAATGGAGAAGATCTTACCGTCGATGTTTACCCCTGTAAAGAGTGTTATGTGTTCCAAAGTTGATAAAATAATGGTTCATGAGAATGAGTCATTGTCAGGGGTGAACCTTCTTAAAGGAGTTAAGCTTATTGATAGTGGATACGTCTGTTTAGCCGGTTTGGTCGTCACGGGCGAGTGGAACTTGCCTGACAATTGCAGAGGAGGTGTGAGCGTGTGTCTGGTGGACAAAAGGATGGAAAGAGCCGACGAGGCCACTCTCGGATCTTACTACAC

**MIGS_TMV_AAA**

**GTGATTTTTCTCTACAAGCGAA**TGCCGTGCATTCGCTTGCAGGTGGATTGCGATCTTTAGAACTGGAATATCTGATGATGCAAATTCCCTACGGATCATTGACTTATGACATAGGCGGGAATTTTGCATCGCATCTGTTCAAGGGACGAGCATATGTACACTGCTGCATGCCCAACCTGGACGTTCGAGACATCATGCGGCACGAAGGCCAGAAAGACAGTATTGAACTATACCTTTCTAGGCTAGAGAGAGGGGGGAAAACAGTCCCCAACTTCCAAAAGGAAGCATTTGACAGATACGCAGAAATTCCTGAAGACGCTGTCTGTCACAATACTTTCCAGACATGCG**GTGATTTTTCTCTACAAGCGAA**TGCCGTGCATTCGCTTGCAGGTGGATTGCGATCTTTAGAACTGGAATATCTGATGATGCAAATTCCCTACGGATCATTGACTTATGACATAGGCGGGAATTTTGCATCGCATCTGTTCAAGGGACGAGCATATGTACACTGCTGCATGCCCAACCTGGACGTTCGAGACATCATGCGGCACGAAGGCCAGAAAGACAGTATTGAACTATACCTTTCTAGGCTAGAGAGAGGGGGGAAAACAGTCCCCAACTTCCAAAAGGAAGCATTTGACAGATACGCAGAAATTCCTGAAGACGCTGTCTGTCACAATACTTTCCAGACATGCG**GTGATTTTTCTCTACAAGCGAA**TGCCGTGCATTCGCTTGCAGGTGGATTGCGATCTTTAGAACTGGAATATCTGATGATGCAAATTCCCTACGGATCATTGACTTATGACATAGGCGGGAATTTTGCATCGCATCTGTTCAAGGGACGAGCATATGTACACTGCTGCATGCCCAACCTGGACGTTCGAGACATCATGCGGCACGAAGGCCAGAAAGACAGTATTGAACTATACCTTTCTAGGCTAGAGAGAGGGGGGAAAACAGTCCCCAACTTCCAAAAGGAAGCATTTGACAGATACGCAGAAATTCCTGAAGACGCTGTCTGTCACAATACTTTCCAGACATGCG

**MIGS_TMV/PVX**

**GTGATTTTTCTCTACAAGCGAA**CAGTTTTACTATGATAAGTGTCTCCCAGGCAACAGCACCATGATGAATAATTTTGATGCTGTTACCATGAGGTTGACTGACATTTCATTGAATGTCAAAGATTGCATATTGGATATGTCTAAGTCTGTTGCTGCGCCTAAGGATCAAATCAAACCACTAATACCTATGGTACGAACGGCGGCAGAAATGCCACGCCAGACTGGACTATTGGAAAATTTAGTGGCGATGATTAAAAGAAACTTTAACGCACCCGAGTTGTCTGGCATCATTGATATTGAAAATACTGCATCTTTGGTTGTAGATAAGTTTTTTGATAGTTATTTGCT**GTGATTTTTCTCTACAAGCGAA**GCTCTAGTTGTTAAAGGAAAAGTGAATATCAATGAGTTTATCGACCTGACAAAAATGGAGAAGATCTTACCGTCGATGTTTACCCCTGTAAAGAGTGTTATGTGTTCCAAAGTTGATAAAATAATGGTTCATGAGAATGAGTCATTGTCAGGGGTGAACCTTCTTAAAGGAGTTAAGCTTATTGATAGTGGATACGTCTGTTTAGCCGGTTTGGTCGTCACGGGCGAGTGGAACTTGCCTGACAATTGCAGAGGAGGTGTGAGCGTGTGTCTGGTGGACAAAAGGATGGAAAGAGCCGACGAGGCCACTCTCGGATCTTACTACAC**GTGATTTTTCTCTACAAGCGAA**GCCAAGGTGCGCGAGGTTTACCAATCTTTTACAGACTCCACCACAAAAACTCTCATCCAAGATGAGGCTTATAGAAACATTCGCCCCATCATGGAAAAACACAAACTAGCTAACCCTTACGCTCAAACGGTTGAAGCGGCTAATGATCTAGAGGGGTTCGGCATAGCCACCAATCCCTATAGCATTGAATTGCATACACATGCAGCCGCTAAGACCATAGAGAATAAACTTCTAGAGGTGCTTGGTTCCATCCTACCACAAGAACCTGTTACATTTATGTTTCTTAAACCCAGAAAGCTAAACTACATGAGAAGAAACCCGCGGAT**GTGATTTTTCTCTACAAGCGAA**CCAGCTAGCACAACACAGCCCATAGGGTCAACTACCTCAACTACCACAAAAACTGCAGGCGCAACTCCTGCCACAGCTTCAGGCCTGTTCACTATCCCGGATGGGGATTTCTTTAGTACAGCCCGTGCCATAGTAGCCAGCAATGCTGTCGCAACAAATGAGGACCTCAGCAAGATTGAGGCTATTTGGAAGGACATGAAGGTGCCCACAGACACTATGGCACAGGCTGCTTGGGACTTAGTCAGACACTGTGCTGATGTAGGATCATCCGCTCAAACAGAAATGATAGATACAGGTCCCTATTCCAACGGCATCAGCAGAGCTAG

**MIGS_3X**

**GTGATTTTTCTCTACAAGCGAA**CGGCTGGGTCTGATTGTTACGAACTGATGCGCCGAGATGGAGTAGTCATCTTCTCTGATGTAGGGGTAGGTCGCCGGTTCTCGTGCTCCTCTACATGTCGACGATAGTGGTCGGAGTCAAGCATGAAGTCCCAGGGTGTAGCGCTGCCAAATCACCAATTCGGAAGCACACTATTCGCTTATTGGCGGGCCCCCTCATCCGAAGTTCCACTTCGTTAGTAGTAAATTACTCCGGGTCTACGCAGAAAGAGTCCACTGCCGGCACAAGCAAGAGCCAACGGTATACGTCAGTGATACTCGC**GTGATTTTTCTCTACAAGCGAA**ACTCACGATTATGACGAATTCCAGTTTGGTGTAATCACGGTGCAAACATAGAGAAACCTAACTCTCTTCCCCGGAAGGTGAGCGGCTAAGATGTCTCTAGAAAGTAACGGGAGTAGGCGAGTTATTCGAATTGTATCTAGTTTTCCACCGAGTGGCACGTTCGATACCTACTAACCTGTAGAATATTGCACCAACTGTATATACGGTTTGCAGTAGTGGATCTGTCCATTCTTCATGCCGTCGGTCCGTAGTTACGCTAAGCTAAAAATGGGCCGGGCTCATACACGCATGGAACAACTC**GTGATTTTTCTCTACAAGCGAA**TATGAGCTCCCGGGGTAACTCTGGTGCGTCGATGAATGACGGTCCTCTGGTAGAAGCGGGTGCTTCATGGGGTTCGTTTAAGGCTCCATGAATCTCTAAGTGGAGGGCTTGCCATGTGCTTCGTCGAGCTTAGGCTCTAATCGGAGTTGACAATCTCACCACCTAGGTCTACACTTTCTGTCGAAGATATTAGCGGAGTGGAAATGTCAGTATTCAGGCGACTGTCTAAGTGGCGCCGTTAACGGTTTTATAACGAGCAAATACGAGATCAACGAAAAGCCGTGCACCGGCCACGATAAT

**MIGS_6X**

**GTGATTTTTCTCTACAAGCGAA**CGGCTGGGTCTGATTGTTACGAACTGATGCGCCGAGATGGAGTAGTCATCTTCTCTGATGTAGGGGTAGGTCGCCGGTTCTCGTGCTCCTCTACATGTCGACGATAGTGGTCGGAGTCAAGCATGAAGTCCCAGGGTGTAGCGCTGCCAAATCACCAATTCGGAAGCACACTATTCGCTTATTGGCGGGCCCCCTCATCCGAAGTTCCACTTCGTTAGTAGTAAATTACTCCGGGTCTACGCAGAAAGAGTCCACTGCCGGCACAAGCAAGAGCCAACGGTATACGTCAGTGATACTCGC**GTGATTTTTCTCTACAAGCGAA**ACTCACGATTATGACGAATTCCAGTTTGGTGTAATCACGGTGCAAACATAGAGAAACCTAACTCTCTTCCCCGGAAGGTGAGCGGCTAAGATGTCTCTAGAAAGTAACGGGAGTAGGCGAGTTATTCGAATTGTATCTAGTTTTCCACCGAGTGGCACGTTCGATACCTACTAACCTGTAGAATATTGCACCAACTGTATATACGGTTTGCAGTAGTGGATCTGTCCATTCTTCATGCCGTCGGTCCGTAGTTACGCTAAGCTAAAAATGGGCCGGGCTCATACACGCATGGAACAACTC**GTGATTTTTCTCTACAAGCGAA**TATGAGCTCCCGGGGTAACTCTGGTGCGTCGATGAATGACGGTCCTCTGGTAGAAGCGGGTGCTTCATGGGGTTCGTTTAAGGCTCCATGAATCTCTAAGTGGAGGGCTTGCCATGTGCTTCGTCGAGCTTAGGCTCTAATCGGAGTTGACAATCTCACCACCTAGGTCTACACTTTCTGTCGAAGATATTAGCGGAGTGGAAATGTCAGTATTCAGGCGACTGTCTAAGTGGCGCCGTTAACGGTTTTATAACGAGCAAATACGAGATCAACGAAAAGCCGTGCACCGGCCACGATAATCCTGCAGG**GTGATTTTTCTCTACAAGCGAA**CTCGGCCACTCTGAGGTGATTTCCGAACGGTCGAGGTCCCTATTGGTCGACACTACACCAGTCCATCATTACCGCACCTTCGGCGGGCGACAACATGGTTAGTTTCGGAATGTATCATGATAACTCACTGGCAGATAAGGGAGATGCGGTTTTAGCACGTCGTACGGTTCATTGCGCTATAAGAAAATAATGCATCGGTGAGCTTTGTTCAACAATAGAACGTCTTACGTCCATCCACTTCTAGCTCCGTTTAGCTAACATTGAGTAGGTGGAAATGAACATATCGCCTTTTTAGGGGTA**GTGATTTTTCTCTACAAGCGAA**ATCCGAGTGTAACTTTCACTACGCAGTTCCTCCAAAGAACTTTGACTGATGCAGAAAGGCAATACACTCATCTCTCTCCACTTCTGTTTCGTATGTGTATGTTTTGCGTATGTCTCGAGGCGTAATATTCGACGCATAATGATGAGGTACGTCGTGATGAGCGCCCAGCAAAGAAGGGATCAGCCGGCCGGCGGGATCGGGTCGACAGCGGCTACCACCTCTATTCGTCCTTACTGCTCTGACATAGGTAAAACCTGCAAAGTCCTGTAAGGGGCCCGGCAGTGTTCGTCAACTTTCTCT**GTGATTTTTCTCTACAAGCGAA**AGTGTCGGAGAATTGCCAGCCGGGGGCTTAGCTGTCCGAATGTACGGATTTCCACGGGAATCTTAACTATCCCCTATGTAATACTAACAATCCGTGCCTTAAACCATGCGATGTACCGGACCGGTTGAAGGAACGTGCAGATCCTACAAAAAATAGACAATTGACCCGTGTTTGATACTTATGTCCGTACCTGCGCCCTGGTAATAAGACAAAATCCGTTAGGCCTCGAATCGAGGCGTCATGCCGAAACGAGGCTCCGGCCCACATAGTTCATAGGCACCATCTAACCGCCAACTATGT

**MIGS_9X**

**GTGATTTTTCTCTACAAGCGAA**CGGCTGGGTCTGATTGTTACGAACTGATGCGCCGAGATGGAGTAGTCATCTTCTCTGATGTAGGGGTAGGTCGCCGGTTCTCGTGCTCCTCTACATGTCGACGATAGTGGTCGGAGTCAAGCATGAAGTCCCAGGGTGTAGCGCTGCCAAATCACCAATTCGGAAGCACACTATTCGCTTATTGGCGGGCCCCCTCATCCGAAGTTCCACTTCGTTAGTAGTAAATTACTCCGGGTCTACGCAGAAAGAGTCCACTGCCGGCACAAGCAAGAGCCAACGGTATACGTCAGTGATACTCGC**GTGATTTTTCTCTACAAGCGAA**ACTCACGATTATGACGAATTCCAGTTTGGTGTAATCACGGTGCAAACATAGAGAAACCTAACTCTCTTCCCCGGAAGGTGAGCGGCTAAGATGTCTCTAGAAAGTAACGGGAGTAGGCGAGTTATTCGAATTGTATCTAGTTTTCCACCGAGTGGCACGTTCGATACCTACTAACCTGTAGAATATTGCACCAACTGTATATACGGTTTGCAGTAGTGGATCTGTCCATTCTTCATGCCGTCGGTCCGTAGTTACGCTAAGCTAAAAATGGGCCGGGCTCATACACGCATGGAACAACTC**GTGATTTTTCTCTACAAGCGAA**TATGAGCTCCCGGGGTAACTCTGGTGCGTCGATGAATGACGGTCCTCTGGTAGAAGCGGGTGCTTCATGGGGTTCGTTTAAGGCTCCATGAATCTCTAAGTGGAGGGCTTGCCATGTGCTTCGTCGAGCTTAGGCTCTAATCGGAGTTGACAATCTCACCACCTAGGTCTACACTTTCTGTCGAAGATATTAGCGGAGTGGAAATGTCAGTATTCAGGCGACTGTCTAAGTGGCGCCGTTAACGGTTTTATAACGAGCAAATACGAGATCAACGAAAAGCCGTGCACCGGCCACGATAATCCTGCAGG**GTGATTTTTCTCTACAAGCGAA**CTCGGCCACTCTGAGGTGATTTCCGAACGGTCGAGGTCCCTATTGGTCGACACTACACCAGTCCATCATTACCGCACCTTCGGCGGGCGACAACATGGTTAGTTTCGGAATGTATCATGATAACTCACTGGCAGATAAGGGAGATGCGGTTTTAGCACGTCGTACGGTTCATTGCGCTATAAGAAAATAATGCATCGGTGAGCTTTGTTCAACAATAGAACGTCTTACGTCCATCCACTTCTAGCTCCGTTTAGCTAACATTGAGTAGGTGGAAATGAACATATCGCCTTTTTAGGGGTA**GTGATTTTTCTCTACAAGCGAA**ATCCGAGTGTAACTTTCACTACGCAGTTCCTCCAAAGAACTTTGACTGATGCAGAAAGGCAATACACTCATCTCTCTCCACTTCTGTTTCGTATGTGTATGTTTTGCGTATGTCTCGAGGCGTAATATTCGACGCATAATGATGAGGTACGTCGTGATGAGCGCCCAGCAAAGAAGGGATCAGCCGGCCGGCGGGATCGGGTCGACAGCGGCTACCACCTCTATTCGTCCTTACTGCTCTGACATAGGTAAAACCTGCAAAGTCCTGTAAGGGGCCCGGCAGTGTTCGTCAACTTTCTCT**GTGATTTTTCTCTACAAGCGAA**AGTGTCGGAGAATTGCCAGCCGGGGGCTTAGCTGTCCGAATGTACGGATTTCCACGGGAATCTTAACTATCCCCTATGTAATACTAACAATCCGTGCCTTAAACCATGCGATGTACCGGACCGGTTGAAGGAACGTGCAGATCCTACAAAAAATAGACAATTGACCCGTGTTTGATACTTATGTCCGTACCTGCGCCCTGGTAATAAGACAAAATCCGTTAGGCCTCGAATCGAGGCGTCATGCCGAAACGAGGCTCCGGCCCACATAGTTCATAGGCACCATCTAACCGCCAACTATGTATCGAT**GTGATTTTTCTCTACAAGCGAA**GGATTGAATGCGACGGGTGGTTTCGTTGTTAGTGGATGCACTGTTAGACCGGAATCGAAACTTTGGGTGTTAGGACACAGGATGGTGACTAACTAGTCACGCGAGCCATCACCTCGAGCTAAAGAGAATCAGCTTCGTTTTCCTGCTGACAGGTTCGTTTTTTTAGGCTACTTCAATGGGATCTCGCCCGGGCTGCACACCATACGTCCTGTAATCATGAAGCAGCGGAGCGCTTCTGCGGCGCGTGACCTTCAAGTGCTGGAGTATGTAATAAGGCTACTGTATGAGCGCGAGGAGAGG**GTGATTTTTCTCTACAAGCGAA**GGCTGTGCCCACAAAGCTCAATTTCCTAGTAATCGTATGCAACCTCACGAGAATCGTGCTTTACTTGTCACAATCGTAGCCTCGCGGGGCGATAAGATGGTTCCATATCATTATCCGGAAACTTCTACGACCCAGGGTAATTACAAACAACATGCAAAAAGAGAGAGTACGAGCGGACGTACGAGACCGCACAACGGGGTTCCGACTGAGTGAGGGGGGCAACGTTACACATTTCCGGCCGAATAAGGTCCCAGAAGTTAGTGTATCGTAGTTGGTCGTACGGGTTGCGAGTATAATGGC**GTGATTTTTCTCTACAAGCGAA**TGCGTGGCCGCCCTAGGACCGCAGACGACGGAATGTCTTCTTGCGTCAACAAATCAACTTGACCTACGTTCGATCTGCTGCCCCTGCGAAGATTTCCTTGGACATGTGACTCCTCTAAAAGAATATACCCTCAAAAGATACGTGTGTCAGAGCGGCACCGTCATCTTCCGTAGAGTCGGGCCGATGCCAACTCGAGATCGGGAAATACTTGACCTTTACCCTGATTATCCGAACGGTCCCTCTGATCAGGGCAAAAGTCCAGAGACTTTATTTATTCAGAGGTTTCGGACCCTGCGGTAG

**MIGS_12X**

**GTGATTTTTCTCTACAAGCGAA**CGGCTGGGTCTGATTGTTACGAACTGATGCGCCGAGATGGAGTAGTCATCTTCTCTGATGTAGGGGTAGGTCGCCGGTTCTCGTGCTCCTCTACATGTCGACGATAGTGGTCGGAGTCAAGCATGAAGTCCCAGGGTGTAGCGCTGCCAAATCACCAATTCGGAAGCACACTATTCGCTTATTGGCGGGCCCCCTCATCCGAAGTTCCACTTCGTTAGTAGTAAATTACTCCGGGTCTACGCAGAAAGAGTCCACTGCCGGCACAAGCAAGAGCCAACGGTATACGTCAGTGATACTCGC**GTGATTTTTCTCTACAAGCGAA**ACTCACGATTATGACGAATTCCAGTTTGGTGTAATCACGGTGCAAACATAGAGAAACCTAACTCTCTTCCCCGGAAGGTGAGCGGCTAAGATGTCTCTAGAAAGTAACGGGAGTAGGCGAGTTATTCGAATTGTATCTAGTTTTCCACCGAGTGGCACGTTCGATACCTACTAACCTGTAGAATATTGCACCAACTGTATATACGGTTTGCAGTAGTGGATCTGTCCATTCTTCATGCCGTCGGTCCGTAGTTACGCTAAGCTAAAAATGGGCCGGGCTCATACACGCATGGAACAACTC**GTGATTTTTCTCTACAAGCGAA**TATGAGCTCCCGGGGTAACTCTGGTGCGTCGATGAATGACGGTCCTCTGGTAGAAGCGGGTGCTTCATGGGGTTCGTTTAAGGCTCCATGAATCTCTAAGTGGAGGGCTTGCCATGTGCTTCGTCGAGCTTAGGCTCTAATCGGAGTTGACAATCTCACCACCTAGGTCTACACTTTCTGTCGAAGATATTAGCGGAGTGGAAATGTCAGTATTCAGGCGACTGTCTAAGTGGCGCCGTTAACGGTTTTATAACGAGCAAATACGAGATCAACGAAAAGCCGTGCACCGGCCACGATAATCCTGCAGG**GTGATTTTTCTCTACAAGCGAA**CTCGGCCACTCTGAGGTGATTTCCGAACGGTCGAGGTCCCTATTGGTCGACACTACACCAGTCCATCATTACCGCACCTTCGGCGGGCGACAACATGGTTAGTTTCGGAATGTATCATGATAACTCACTGGCAGATAAGGGAGATGCGGTTTTAGCACGTCGTACGGTTCATTGCGCTATAAGAAAATAATGCATCGGTGAGCTTTGTTCAACAATAGAACGTCTTACGTCCATCCACTTCTAGCTCCGTTTAGCTAACATTGAGTAGGTGGAAATGAACATATCGCCTTTTTAGGGGTA**GTGATTTTTCTCTACAAGCGAA**ATCCGAGTGTAACTTTCACTACGCAGTTCCTCCAAAGAACTTTGACTGATGCAGAAAGGCAATACACTCATCTCTCTCCACTTCTGTTTCGTATGTGTATGTTTTGCGTATGTCTCGAGGCGTAATATTCGACGCATAATGATGAGGTACGTCGTGATGAGCGCCCAGCAAAGAAGGGATCAGCCGGCCGGCGGGATCGGGTCGACAGCGGCTACCACCTCTATTCGTCCTTACTGCTCTGACATAGGTAAAACCTGCAAAGTCCTGTAAGGGGCCCGGCAGTGTTCGTCAACTTTCTCT**GTGATTTTTCTCTACAAGCGAA**AGTGTCGGAGAATTGCCAGCCGGGGGCTTAGCTGTCCGAATGTACGGATTTCCACGGGAATCTTAACTATCCCCTATGTAATACTAACAATCCGTGCCTTAAACCATGCGATGTACCGGACCGGTTGAAGGAACGTGCAGATCCTACAAAAAATAGACAATTGACCCGTGTTTGATACTTATGTCCGTACCTGCGCCCTGGTAATAAGACAAAATCCGTTAGGCCTCGAATCGAGGCGTCATGCCGAAACGAGGCTCCGGCCCACATAGTTCATAGGCACCATCTAACCGCCAACTATGTATCGAT**GTGATTTTTCTCTACAAGCGAA**GGATTGAATGCGACGGGTGGTTTCGTTGTTAGTGGATGCACTGTTAGACCGGAATCGAAACTTTGGGTGTTAGGACACAGGATGGTGACTAACTAGTCACGCGAGCCATCACCTCGAGCTAAAGAGAATCAGCTTCGTTTTCCTGCTGACAGGTTCGTTTTTTTAGGCTACTTCAATGGGATCTCGCCCGGGCTGCACACCATACGTCCTGTAATCATGAAGCAGCGGAGCGCTTCTGCGGCGCGTGACCTTCAAGTGCTGGAGTATGTAATAAGGCTACTGTATGAGCGCGAGGAGAGG**GTGATTTTTCTCTACAAGCGAA**GGCTGTGCCCACAAAGCTCAATTTCCTAGTAATCGTATGCAACCTCACGAGAATCGTGCTTTACTTGTCACAATCGTAGCCTCGCGGGGCGATAAGATGGTTCCATATCATTATCCGGAAACTTCTACGACCCAGGGTAATTACAAACAACATGCAAAAAGAGAGAGTACGAGCGGACGTACGAGACCGCACAACGGGGTTCCGACTGAGTGAGGGGGGCAACGTTACACATTTCCGGCCGAATAAGGTCCCAGAAGTTAGTGTATCGTAGTTGGTCGTACGGGTTGCGAGTATAATGGC**GTGATTTTTCTCTACAAGCGAA**TGCGTGGCCGCCCTAGGACCGCAGACGACGGAATGTCTTCTTGCGTCAACAAATCAACTTGACCTACGTTCGATCTGCTGCCCCTGCGAAGATTTCCTTGGACATGTGACTCCTCTAAAAGAATATACCCTCAAAAGATACGTGTGTCAGAGCGGCACCGTCATCTTCCGTAGAGTCGGGCCGATGCCAACTCGAGATCGGGAAATACTTGACCTTTACCCTGATTATCCGAACGGTCCCTCTGATCAGGGCAAAAGTCCAGAGACTTTATTTATTCAGAGGTTTCGGACCCTGCGGTAGTTAATTAA**GTGATTTTTCTCTACAAGCGAA**GGTGATTTTTCGCATATTTGCGCCCACAGGAAAGTACCTGACTACGTTCTACGTACGTCCCCATTAACCATCACGGAAACTTCTAACAATGAAAGTCGATGATTGCGATGGTGATTGAGAGGGGCTATAAGATTCGGAAGTGCAGCATTTTGGGAGTGGCCCGAAGCCAGCCCGATTTTGTAAATGATGGATTTGGTCTATACCGTGCCCCGCACTATGAAGCACCGGTGCCGCAAAATTAGATTACTTTCGTCGTTCTGGCAGCGCCCCGTTCAAGAAATTGTCTAGTTAGAGGTTTCA**GTGATTTTTCTCTACAAGCGAA**AGGAGAGAATGCTTCGGGGTTCCGGGAGAGCCGAGTACGCGATAACCCTCGACCGGGTGTTTGTAGCGTCGCACTTCAACCTTAGTACATCGAGCGCGGACCGACCTGCCCCAAAAACCCACGGCGCGGGAGAACCCTACAGCCCCATCGTTTACAGTCGCGTACCCGGCTATTGCCTGTTGACTTTGGGGTCTAGGTGCGACAGATGGACGTACCTACTCAGGCCCGAGTACGGGATAATATAATGGTCCCGCATCCGTCGCTATAAAAAATGTCGCCTTCTGGGCCACCAAGGCAAGA**GTGATTTTTCTCTACAAGCGAA**CATCAATGGACAACCTTCCGTTATGTTTAGAGAAGGGGGGGGTTGCAATAGGCAGTTGTCAGTATAAGGTAAGGATCTAAAGCGATGCTGAGTCATCAGCTACCAAATACTCACCATGAAGCCGTCGCCTCTGAAGGTTTCGCTTTTTTATAGGCCGTGTCTGTGTCGGTATATGGCCAGTGCCAGATTACGCATCGCCCCCTAAGGGGGGGCTATTACACAACAGTCATTCGCATTTAGGCATAAAGGCTCACCGTACATAGGTGTCGCATCGCCGTCACGAGTGGACCGGGATGCG

**MIGS_15X**

**GTGATTTTTCTCTACAAGCGAA**CGGCTGGGTCTGATTGTTACGAACTGATGCGCCGAGATGGAGTAGTCATCTTCTCTGATGTAGGGGTAGGTCGCCGGTTCTCGTGCTCCTCTACATGTCGACGATAGTGGTCGGAGTCAAGCATGAAGTCCCAGGGTGTAGCGCTGCCAAATCACCAATTCGGAAGCACACTATTCGCTTATTGGCGGGCCCCCTCATCCGAAGTTCCACTTCGTTAGTAGTAAATTACTCCGGGTCTACGCAGAAAGAGTCCACTGCCGGCACAAGCAAGAGCCAACGGTATACGTCAGTGATACTCGC**GTGATTTTTCTCTACAAGCGAA**ACTCACGATTATGACGAATTCCAGTTTGGTGTAATCACGGTGCAAACATAGAGAAACCTAACTCTCTTCCCCGGAAGGTGAGCGGCTAAGATGTCTCTAGAAAGTAACGGGAGTAGGCGAGTTATTCGAATTGTATCTAGTTTTCCACCGAGTGGCACGTTCGATACCTACTAACCTGTAGAATATTGCACCAACTGTATATACGGTTTGCAGTAGTGGATCTGTCCATTCTTCATGCCGTCGGTCCGTAGTTACGCTAAGCTAAAAATGGGCCGGGCTCATACACGCATGGAACAACTC**GTGATTTTTCTCTACAAGCGAA**TATGAGCTCCCGGGGTAACTCTGGTGCGTCGATGAATGACGGTCCTCTGGTAGAAGCGGGTGCTTCATGGGGTTCGTTTAAGGCTCCATGAATCTCTAAGTGGAGGGCTTGCCATGTGCTTCGTCGAGCTTAGGCTCTAATCGGAGTTGACAATCTCACCACCTAGGTCTACACTTTCTGTCGAAGATATTAGCGGAGTGGAAATGTCAGTATTCAGGCGACTGTCTAAGTGGCGCCGTTAACGGTTTTATAACGAGCAAATACGAGATCAACGAAAAGCCGTGCACCGGCCACGATAATCCTGCAGG**GTGATTTTTCTCTACAAGCGAA**CTCGGCCACTCTGAGGTGATTTCCGAACGGTCGAGGTCCCTATTGGTCGACACTACACCAGTCCATCATTACCGCACCTTCGGCGGGCGACAACATGGTTAGTTTCGGAATGTATCATGATAACTCACTGGCAGATAAGGGAGATGCGGTTTTAGCACGTCGTACGGTTCATTGCGCTATAAGAAAATAATGCATCGGTGAGCTTTGTTCAACAATAGAACGTCTTACGTCCATCCACTTCTAGCTCCGTTTAGCTAACATTGAGTAGGTGGAAATGAACATATCGCCTTTTTAGGGGTA**GTGATTTTTCTCTACAAGCGAA**ATCCGAGTGTAACTTTCACTACGCAGTTCCTCCAAAGAACTTTGACTGATGCAGAAAGGCAATACACTCATCTCTCTCCACTTCTGTTTCGTATGTGTATGTTTTGCGTATGTCTCGAGGCGTAATATTCGACGCATAATGATGAGGTACGTCGTGATGAGCGCCCAGCAAAGAAGGGATCAGCCGGCCGGCGGGATCGGGTCGACAGCGGCTACCACCTCTATTCGTCCTTACTGCTCTGACATAGGTAAAACCTGCAAAGTCCTGTAAGGGGCCCGGCAGTGTTCGTCAACTTTCTCT**GTGATTTTTCTCTACAAGCGAA**AGTGTCGGAGAATTGCCAGCCGGGGGCTTAGCTGTCCGAATGTACGGATTTCCACGGGAATCTTAACTATCCCCTATGTAATACTAACAATCCGTGCCTTAAACCATGCGATGTACCGGACCGGTTGAAGGAACGTGCAGATCCTACAAAAAATAGACAATTGACCCGTGTTTGATACTTATGTCCGTACCTGCGCCCTGGTAATAAGACAAAATCCGTTAGGCCTCGAATCGAGGCGTCATGCCGAAACGAGGCTCCGGCCCACATAGTTCATAGGCACCATCTAACCGCCAACTATGTATCGAT**GTGATTTTTCTCTACAAGCGAA**GGATTGAATGCGACGGGTGGTTTCGTTGTTAGTGGATGCACTGTTAGACCGGAATCGAAACTTTGGGTGTTAGGACACAGGATGGTGACTAACTAGTCACGCGAGCCATCACCTCGAGCTAAAGAGAATCAGCTTCGTTTTCCTGCTGACAGGTTCGTTTTTTTAGGCTACTTCAATGGGATCTCGCCCGGGCTGCACACCATACGTCCTGTAATCATGAAGCAGCGGAGCGCTTCTGCGGCGCGTGACCTTCAAGTGCTGGAGTATGTAATAAGGCTACTGTATGAGCGCGAGGAGAGG**GTGATTTTTCTCTACAAGCGAA**GGCTGTGCCCACAAAGCTCAATTTCCTAGTAATCGTATGCAACCTCACGAGAATCGTGCTTTACTTGTCACAATCGTAGCCTCGCGGGGCGATAAGATGGTTCCATATCATTATCCGGAAACTTCTACGACCCAGGGTAATTACAAACAACATGCAAAAAGAGAGAGTACGAGCGGACGTACGAGACCGCACAACGGGGTTCCGACTGAGTGAGGGGGGCAACGTTACACATTTCCGGCCGAATAAGGTCCCAGAAGTTAGTGTATCGTAGTTGGTCGTACGGGTTGCGAGTATAATGGC**GTGATTTTTCTCTACAAGCGAA**TGCGTGGCCGCCCTAGGACCGCAGACGACGGAATGTCTTCTTGCGTCAACAAATCAACTTGACCTACGTTCGATCTGCTGCCCCTGCGAAGATTTCCTTGGACATGTGACTCCTCTAAAAGAATATACCCTCAAAAGATAGTGTGTCAGAGCGGCACCGTCATCTTCCGTAGAGTCGGGCCGATGCCAACTCGAGATCGGGAAATACTTGACCTTTACCCTGATTATCCGAACGGTCCCTCTGATCAGGGCAAAAGTCCAGAGACTTTATTTATTCAGAGGTTTCGGACCCTGCGGTAGTTAATTAA**GTGATTTTTCTCTACAAGCGAA**GGTGATTTTTCGCATATTTGCGCCCACAGGAAAGTACCTGACTACGTTCTACGTACGTCCCCATTAACCATCACGGAAACTTCTAACAATGAAAGTCGATGATTGCGATGGTGATTGAGAGGGGCTATAAGATTCGGAAGTGCAGCATTTTGGGAGTGGCCCGAAGCCAGCCCGATTTTGTAAATGATGGATTTGGTCTATACCGTGCCCCGCACTATGAAGCACCGGTGCCGCAAAATTAGATTACTTTCGTCGTTCTGGCAGCGCCCCGTTCAAGAAATTGTCTAGTTAGAGGTTTCA**GTGATTTTTCTCTACAAGCGAA**AGGAGAGAATGCTTCGGGGTTCCGGGAGAGCCGAGTACGCGATAACCCTCGACCGGGTGTTTGTAGCGTCGCACTTCAACCTTAGTACATCGAGCGCGGACCGACCTGCCCCAAAAACCCACGGCGCGGGAGAACCCTACAGCCCCATCGTTTACAGTCGCGTACCCGGCTATTGCCTGTTGACTTTGGGGTCTAGGTGCGACAGATGGACGTACCTACTCAGGCCCGAGTACGGGATAATATAATGGTCCCGCATCCGTCGCTATAAAAAATGTCGCCTTCTGGGCCACCAAGGCAAGA**GTGATTTTTCTCTACAAGCGAA**CATCAATGGACAACCTTCCGTTATGTTTAGAGAAGGGGGGGGTTGCAATAGGCAGTTGTCAGTATAAGGTAAGGATCTAAAGCGATGCTGAGTCATCAGCTACCAAATACTCACCATGAAGCCGTCGCCTCTGAAGGTTTCGCTTTTTTATAGGCCGTGTCTGTGTCGGTATATGGCCAGTGCCAGATTACGCATCGCCCCCTAAGGGGGGGCTATTACACAACAGTCATTCGCATTTAGGCATAAATTGGCTCACCGTACATAGGTGTCGCATCGCCGTCACGAGTGGACCGGGATGCGGGTACC**GTGATTTTTCTCTACAAGCGAA**ATTGTGGAAGGTGTAGCTACAGAACTCGATCACCAAGAGTGCACAACTGAATCCATCAGTCGTTGCGTTAGCTATTTCCAACCACGCGATTCGCGCGTAACACCGTTGTGAACGTACGGGGCATGACGTGAAGACCACACGGTGGATAATGAAAGGCATCACGTCCTCTCCGAATCTCGAAATTAACAAGTACCTCTTGAAAGCGATGCTCCCTCAGCGCTTGCGTTATCGCAACGGGAAGTGCCAGCGCTACGATCCGCCACTGTAAGTCCCCGCTCCGTGATATATGGAATTATCTTT**GTGATTTTTCTCTACAAGCGAA**CATCCTCTCCCACTACTGAGCAGCTCCTGCGAGCCCATTGTAGTCTAGGCTTTATGCGGGCTTGATGCGACCGTTTCAACCGGTTGGATACTTGTTAGGCCTGTCGTCTGCAACCCGCACGCGCGTGGGATGGGGGCAATGTCAGTTAGGGGGTCCTTCCTCGACCGACGGTCACGCTCTGTTATCCCTAATAACACAAGGAATTATGTGCGGCTTTTGCCGGGATGCTCTATTTACGTTATCGTTCCCACTAAGACGCTCATAGGGTCGCTACAATGGGGGAAGGGACTTATTTGCTTC**GTGATTTTTCTCTACAAGCGAA**GCGTCTTCTTGCCACGCGGATTCAATAGTTAGATGGTGGTCGTGCCCCCTTACAGCACAGCCCGTAAGATATAAACGAAGCCGGCGTCTCGAAGGATCCGTACTACCAATTGTGTTTACGATTTGGTACAGCAGCTAGTAGTTGGAGGGAGAGGGATGGACAAACGTTATATTACTTTCATCCGCTATATGTTGCATCTTGATGGCGTAAAATGAGCATACCTCAACCCGGAGCCGCCGCCAACTCTGTAAACCTTACGCTGAGTACCCCCGCGGCCCTCCACCTGTTCATAATCGCTCA

**MIGS_6X_TMV_A**

**GTGATTTTTCTCTACAAGCGAA**CGGCTGGGTCTGATTGTTACGAACTGATGCGCCGAGATGGAGTAGTCATCTTCTCTGATGTAGGGGTAGGTCGCCGGTTCTCGTGCTCCTCTACATGTCGACGATAGTGGTCGGAGTCAAGCATGAAGTCCCAGGGTGTAGCGCTGCCAAATCACCAATTCGGAAGCACACTATTCGCTTATTGGCGGGCCCCCTCATCCGAAGTTCCACTTCGTTAGTAGTAAATTACTCCGGGTCTACGCAGAAAGAGTCCACTGCCGGCACAAGCAAGAGCCAACGGTATACGTCAGTGATACTCGC**GTGATTTTTCTCTACAAGCGAA**ACTCACGATTATGACGAATTCCAGTTTGGTGTAATCACGGTGCAAACATAGAGAAACCTAACTCTCTTCCCCGGAAGGTGAGCGGCTAAGATGTCTCTAGAAAGTAACGGGAGTAGGCGAGTTATTCGAATTGTATCTAGTTTTCCACCGAGTGGCACGTTCGATACCTACTAACCTGTAGAATATTGCACCAACTGTATATACGGTTTGCAGTAGTGGATCTGTCCATTCTTCATGCCGTCGGTCCGTAGTTACGCTAAGCTAAAAATGGGCCGGGCTCATACACGCATGGAACAACTC**GTGATTTTTCTCTACAAGCGAA**TATGAGCTCCCGGGGTAACTCTGGTGCGTCGATGAATGACGGTCCTCTGGTAGAAGCGGGTGCTTCATGGGGTTCGTTTAAGGCTCCATGAATCTCTAAGTGGAGGGCTTGCCATGTGCTTCGTCGAGCTTAGGCTCTAATCGGAGTTGACAATCTCACCACCTAGGTCTACACTTTCTGTCGAAGATATTAGCGGAGTGGAAATGTCAGTATTCAGGCGACTGTCTAAGTGGCGCCGTTAACGGTTTTATAACGAGCAAATACGAGATCAACGAAAAGCCGTGCACCGGCCACGATAATCCTGCAGG**GTGATTTTTCTCTACAAGCGAA**CTCGGCCACTCTGAGGTGATTTCCGAACGGTCGAGGTCCCTATTGGTCGACACTACACCAGTCCATCATTACCGCACCTTCGGCGGGCGACAACATGGTTAGTTTCGGAATGTATCATGATAACTCACTGGCAGATAAGGGAGATGCGGTTTTAGCACGTCGTACGGTTCATTGCGCTATAAGAAAATAATGCATCGGTGAGCTTTGTTCAACAATAGAACGTCTTACGTCCATCCACTTCTAGCTCCGTTTAGCTAACATTGAGTAGGTGGAAATGAACATATCGCCTTTTTAGGGGTA**GTGATTTTTCTCTACAAGCGAA**ATCCGAGTGTAACTTTCACTACGCAGTTCCTCCAAAGAACTTTGACTGATGCAGAAAGGCAATACACTCATCTCTCTCCACTTCTGTTTCGTATGTGTATGTTTTGCGTATGTCTCGAGGCGTAATATTCGACGCATAATGATGAGGTACGTCGTGATGAGCGCCCAGCAAAGAAGGGATCAGCCGGCCGGCGGGATCGGGTCGACAGCGGCTACCACCTCTATTCGTCCTTACTGCTCTGACATAGGTAAAACCTGCAAAGTCCTGTAAGGGGCCCGGCAGTGTTCGTCAACTTTCTCT**GTGATTTTTCTCTACAAGCGAA**AGTGTCGGAGAATTGCCAGCCGGGGGCTTAGCTGTCCGAATGTACGGATTTCCACGGGAATCTTAACTATCCCCTATGTAATACTAACAATCCGTGCCTTAAACCATGCGATGTACCGGACCGGTTGAAGGAACGTGCAGATCCTACAAAAAATAGACAATTGACCCGTGTTTGATACTTATGTCCGTACCTGCGCCCTGGTAATAAGACAAAATCCGTTAGGCCTCGAATCGAGGCGTCATGCCGAAACGAGGCTCCGGCCCACATAGTTCATAGGCACCATCTAACCGCCAACTATGTATCGAT**GTGATTTTTCTCTACAAGCGAA**TGCCGTGCATTCGCTTGCAGGTGGATTGCGATCTTTAGAACTGGAATATCTGATGATGCAAATTCCCTACGGATCATTGACTTATGACATAGGCGGGAATTTTGCATCGCATCTGTTCAAGGGACGAGCATATGTACACTGCTGCATGCCCAACCTGGACGTTCGAGACATCATGCGGCACGAAGGCCAGAAAGACAGTATTGAACTATACCTTTCTAGGCTAGAGAGAGGGGGGAAAACAGTCCCCAACTTCCAAAAGGAAGCATTTGACAGATACGCAGAAATTCCTGAAGACGCTGTCTGTCACAATACTTTCCAGACATGCG

**MIGS_9X_TMV_A**

**GTGATTTTTCTCTACAAGCGAA**CGGCTGGGTCTGATTGTTACGAACTGATGCGCCGAGATGGAGTAGTCATCTTCTCTGATGTAGGGGTAGGTCGCCGGTTCTCGTGCTCCTCTACATGTCGACGATAGTGGTCGGAGTCAAGCATGAAGTCCCAGGGTGTAGCGCTGCCAAATCACCAATTCGGAAGCACACTATTCGCTTATTGGCGGGCCCCCTCATCCGAAGTTCCACTTCGTTAGTAGTAAATTACTCCGGGTCTACGCAGAAAGAGTCCACTGCCGGCACAAGCAAGAGCCAACGGTATACGTCAGTGATACTCGC**GTGATTTTTCTCTACAAGCGAA**ACTCACGATTATGACGAATTCCAGTTTGGTGTAATCACGGTGCAAACATAGAGAAACCTAACTCTCTTCCCCGGAAGGTGAGCGGCTAAGATGTCTCTAGAAAGTAACGGGAGTAGGCGAGTTATTCGAATTGTATCTAGTTTTCCACCGAGTGGCACGTTCGATACCTACTAACCTGTAGAATATTGCACCAACTGTATATACGGTTTGCAGTAGTGGATCTGTCCATTCTTCATGCCGTCGGTCCGTAGTTACGCTAAGCTAAAAATGGGCCGGGCTCATACACGCATGGAACAACTC**GTGATTTTTCTCTACAAGCGAA**TATGAGCTCCCGGGGTAACTCTGGTGCGTCGATGAATGACGGTCCTCTGGTAGAAGCGGGTGCTTCATGGGGTTCGTTTAAGGCTCCATGAATCTCTAAGTGGAGGGCTTGCCATGTGCTTCGTCGAGCTTAGGCTCTAATCGGAGTTGACAATCTCACCACCTAGGTCTACACTTTCTGTCGAAGATATTAGCGGAGTGGAAATGTCAGTATTCAGGCGACTGTCTAAGTGGCGCCGTTAACGGTTTTATAACGAGCAAATACGAGATCAACGAAAAGCCGTGCACCGGCCACGATAATCCTGCAGG**GTGATTTTTCTCTACAAGCGAA**CTCGGCCACTCTGAGGTGATTTCCGAACGGTCGAGGTCCCTATTGGTCGACACTACACCAGTCCATCATTACCGCACCTTCGGCGGGCGACAACATGGTTAGTTTCGGAATGTATCATGATAACTCACTGGCAGATAAGGGAGATGCGGTTTTAGCACGTCGTACGGTTCATTGCGCTATAAGAAAATAATGCATCGGTGAGCTTTGTTCAACAATAGAACGTCTTACGTCCATCCACTTCTAGCTCCGTTTAGCTAACATTGAGTAGGTGGAAATGAACATATCGCCTTTTTAGGGGTA**GTGATTTTTCTCTACAAGCGAA**ATCCGAGTGTAACTTTCACTACGCAGTTCCTCCAAAGAACTTTGACTGATGCAGAAAGGCAATACACTCATCTCTCTCCACTTCTGTTTCGTATGTGTATGTTTTGCGTATGTCTCGAGGCGTAATATTCGACGCATAATGATGAGGTACGTCGTGATGAGCGCCCAGCAAAGAAGGGATCAGCCGGCCGGCGGGATCGGGTCGACAGCGGCTACCACCTCTATTCGTCCTTACTGCTCTGACATAGGTAAAACCTGCAAAGTCCTGTAAGGGGCCCGGCAGTGTTCGTCAACTTTCTCT**GTGATTTTTCTCTACAAGCGAA**AGTGTCGGAGAATTGCCAGCCGGGGGCTTAGCTGTCCGAATGTACGGATTTCCACGGGAATCTTAACTATCCCCTATGTAATACTAACAATCCGTGCCTTAAACCATGCGATGTACCGGACCGGTTGAAGGAACGTGCAGATCCTACAAAAAATAGACAATTGACCCGTGTTTGATACTTATGTCCGTACCTGCGCCCTGGTAATAAGACAAAATCCGTTAGGCCTCGAATCGAGGCGTCATGCCGAAACGAGGCTCCGGCCCACATAGTTCATAGGCACCATCTAACCGCCAACTATGTATCGAT**GTGATTTTTCTCTACAAGCGAA**GGATTGAATGCGACGGGTGGTTTCGTTGTTAGTGGATGCACTGTTAGACCGGAATCGAAACTTTGGGTGTTAGGACACAGGATGGTGACTAACTAGTCACGCGAGCCATCACCTCGAGCTAAAGAGAATCAGCTTCGTTTTCCTGCTGACAGGTTCGTTTTTTTAGGCTACTTCAATGGGATCTCGCCCGGGCTGCACACCATACGTCCTGTAATCATGAAGCAGCGGAGCGCTTCTGCGGCGCGTGACCTTCAAGTGCTGGAGTATGTAATAAGGCTACTGTATGAGCGCGAGGAGAGG**GTGATTTTTCTCTACAAGCGAA**GGCTGTGCCCACAAAGCTCAATTTCCTAGTAATCGTATGCAACCTCACGAGAATCGTGCTTTACTTGTCACAATCGTAGCCTCGCGGGGCGATAAGATGGTTCCATATCATTATCCGGAAACTTCTACGACCCAGGGTAATTACAAACAACATGCAAAAAGAGAGAGTACGAGCGGACGTACGAGACCGCACAACGGGGTTCCGACTGAGTGAGGGGGGCAACGTTACACATTTCCGGCCGAATAAGGTCCCAGAAGTTAGTGTATCGTAGTTGGTCGTACGGGTTGCGAGTATAATGGC**GTGATTTTTCTCTACAAGCGAA**TGCGTGGCCGCCCTAGGACCGCAGACGACGGAATGTCTTCTTGCGTCAACAAATCAACTTGACCTACGTTCGATCTGCTGCCCCTGCGAAGATTTCCTTGGACATGTGACTCCTCTAAAAGAATATACCCTCAAAAGATACGTGTGTCAGAGCGGCACCGTCATCTTCCGTAGAGTCGGGCCGATGCCAACTCGAGATCGGGAAATACTTGACCTTTACCCTGATTATCCGAACGGTCCCTCTGATCAGGGCAAAAGTCCAGAGACTTTATTTATTCAGAGGTTTCGGACCCTGCGGTAGTTAATTAA**GTGATTTTTCTCTACAAGCGAA**TGCCGTGCATTCGCTTGCAGGTGGATTGCGATCTTTAGAACTGGAATATCTGATGATGCAAATTCCCTACGGATCATTGACTTATGACATAGGCGGGAATTTTGCATCGCATCTGTTCAAGGGACGAGCATATGTACACTGCTGCATGCCCAACCTGGACGTTCGAGACATCATGCGGCACGAAGGCCAGAAAGACAGTATTGAACTATACCTTTCTAGGCTAGAGAGAGGGGGGAAAACAGTCCCCAACTTCCAAAAGGAAGCATTTGACAGATACGCAGAAATTCCTGAAGACGCTGTCTGTCACAATACTTTCCAGACATGCG

**MIGS_12X_TMV_A**

**GTGATTTTTCTCTACAAGCGAA**CGGCTGGGTCTGATTGTTACGAACTGATGCGCCGAGATGGAGTAGTCATCTTCTCTGATGTAGGGGTAGGTCGCCGGTTCTCGTGCTCCTCTACATGTCGACGATAGTGGTCGGAGTCAAGCATGAAGTCCCAGGGTGTAGCGCTGCCAAATCACCAATTCGGAAGCACACTATTCGCTTATTGGCGGGCCCCCTCATCCGAAGTTCCACTTCGTTAGTAGTAAATTACTCCGGGTCTACGCAGAAAGAGTCCACTGCCGGCACAAGCAAGAGCCAACGGTATACGTCAGTGATACTCGC**GTGATTTTTCTCTACAAGCGAA**ACTCACGATTATGACGAATTCCAGTTTGGTGTAATCACGGTGCAAACATAGAGAAACCTAACTCTCTTCCCCGGAAGGTGAGCGGCTAAGATGTCTCTAGAAAGTAACGGGAGTAGGCGAGTTATTCGAATTGTATCTAGTTTTCCACCGAGTGGCACGTTCGATACCTACTAACCTGTAGAATATTGCACCAACTGTATATACGGTTTGCAGTAGTGGATCTGTCCATTCTTCATGCCGTCGGTCCGTAGTTACGCTAAGCTAAAAATGGGCCGGGCTCATACACGCATGGAACAACTC**GTGATTTTTCTCTACAAGCGAA**TATGAGCTCCCGGGGTAACTCTGGTGCGTCGATGAATGACGGTCCTCTGGTAGAAGCGGGTGCTTCATGGGGTTCGTTTAAGGCTCCATGAATCTCTAAGTGGAGGGCTTGCCATGTGCTTCGTCGAGCTTAGGCTCTAATCGGAGTTGACAATCTCACCACCTAGGTCTACACTTTCTGTCGAAGATATTAGCGGAGTGGAAATGTCAGTATTCAGGCGACTGTCTAAGTGGCGCCGTTAACGGTTTTATAACGAGCAAATACGAGATCAACGAAAAGCCGTGCACCGGCCACGATAATCCTGCAGG**GTGATTTTTCTCTACAAGCGAA**CTCGGCCACTCTGAGGTGATTTCCGAACGGTCGAGGTCCCTATTGGTCGACACTACACCAGTCCATCATTACCGCACCTTCGGCGGGCGACAACATGGTTAGTTTCGGAATGTATCATGATAACTCACTGGCAGATAAGGGAGATGCGGTTTTAGCACGTCGTACGGTTCATTGCGCTATAAGAAAATAATGCATCGGTGAGCTTTGTTCAACAATAGAACGTCTTACGTCCATCCACTTCTAGCTCCGTTTAGCTAACATTGAGTAGGTGGAAATGAACATATCGCCTTTTTAGGGGTA**GTGATTTTTCTCTACAAGCGAA**ATCCGAGTGTAACTTTCACTACGCAGTTCCTCCAAAGAACTTTGACTGATGCAGAAAGGCAATACACTCATCTCTCTCCACTTCTGTTTCGTATGTGTATGTTTTGCGTATGTCTCGAGGCGTAATATTCGACGCATAATGATGAGGTACGTCGTGATGAGCGCCCAGCAAAGAAGGGATCAGCCGGCCGGCGGGATCGGGTCGACAGCGGCTACCACCTCTATTCGTCCTTACTGCTCTGACATAGGTAAAACCTGCAAAGTCCTGTAAGGGGCCCGGCAGTGTTCGTCAACTTTCTCT**GTGATTTTTCTCTACAAGCGAA**AGTGTCGGAGAATTGCCAGCCGGGGGCTTAGCTGTCCGAATGTACGGATTTCCACGGGAATCTTAACTATCCCCTATGTAATACTAACAATCCGTGCCTTAAACCATGCGATGTACCGGACCGGTTGAAGGAACGTGCAGATCCTACAAAAAATAGACAATTGACCCGTGTTTGATACTTATGTCCGTACCTGCGCCCTGGTAATAAGACAAAATCCGTTAGGCCTCGAATCGAGGCGTCATGCCGAAACGAGGCTCCGGCCCACATAGTTCATAGGCACCATCTAACCGCCAACTATGTATCGAT**GTGATTTTTCTCTACAAGCGAA**GGATTGAATGCGACGGGTGGTTTCGTTGTTAGTGGATGCACTGTTAGACCGGAATCGAAACTTTGGGTGTTAGGACACAGGATGGTGACTAACTAGTCACGCGAGCCATCACCTCGAGCTAAAGAGAATCAGCTTCGTTTTCCTGCTGACAGGTTCGTTTTTTTAGGCTACTTCAATGGGATCTCGCCCGGGCTGCACACCATACGTCCTGTAATCATGAAGCAGCGGAGCGCTTCTGCGGCGCGTGACCTTCAAGTGCTGGAGTATGTAATAAGGCTACTGTATGAGCGCGAGGAGAGG**GTGATTTTTCTCTACAAGCGAA**GGCTGTGCCCACAAAGCTCAATTTCCTAGTAATCGTATGCAACCTCACGAGAATCGTGCTTTACTTGTCACAATCGTAGCCTCGCGGGGCGATAAGATGGTTCCATATCATTATCCGGAAACTTCTACGACCCAGGGTAATTACAAACAACATGCAAAAAGAGAGAGTACGAGCGGACGTACGAGACCGCACAACGGGGTTCCGACTGAGTGAGGGGGGCAACGTTACACATTTCCGGCCGAATAAGGTCCCAGAAGTTAGTGTATCGTAGTTGGTCGTACGGGTTGCGAGTATAATGGC**GTGATTTTTCTCTACAAGCGAA**TGCGTGGCCGCCCTAGGACCGCAGACGACGGAATGTCTTCTTGCGTCAACAAATCAACTTGACCTACGTTCGATCTGCTGCCCCTGCGAAGATTTCCTTGGACATGTGACTCCTCTAAAAGAATATACCCTCAAAAGATACGTGTGTCAGAGCGGCACCGTCATCTTCCGTAGAGTCGGGCCGATGCCAACTCGAGATCGGGAAATACTTGACCTTTACCCTGATTATCCGAACGGTCCCTCTGATCAGGGCAAAAGTCCAGAGACTTTATTTATTCAGAGGTTTCGGACCCTGCGGTAGTTAATTAA**GTGATTTTTCTCTACAAGCGAA**GGTGATTTTTCGCATATTTGCGCCCACAGGAAAGTACCTGACTACGTTCTACGTACGTCCCCATTAACCATCACGGAAACTTCTAACAATGAAAGTCGATGATTGCGATGGTGATTGAGAGGGGCTATAAGATTCGGAAGTGCAGCATTTTGGGAGTGGCCCGAAGCCAGCCCGATTTTGTAAATGATGGATTTGGTCTATACCGTGCCCCGCACTATGAAGCACCGGTGCCGCAAAATTAGATTACTTTCGTCGTTCTGGCAGCGCCCCGTTCAAGAAATTGTCTAGTTAGAGGTTTCA**GTGATTTTTCTCTACAAGCGAA**AGGAGAGAATGCTTCGGGGTTCCGGGAGAGCCGAGTACGCGATAACCCTCGACCGGGTGTTTGTAGCGTCGCACTTCAACCTTAGTACATCGAGCGCGGACCGACCTGCCCCAAAAACCCACGGCGCGGGAGAACCCTACAGCCCCATCGTTTACAGTCGCGTACCCGGCTATTGCCTGTTGACTTTGGGGTCTAGGTGCGACAGATGGACGTACCTACTCAGGCCCGAGTACGGGATAATATAATGGTCCCGCATCCGTCGCTATAAAAAATGTCGCCTTCTGGGCCACCAAGGCAAGA**GTGATTTTTCTCTACAAGCGAA**CATCAATGGACAACCTTCCGTTATGTTTAGAGAAGGGGGGGGTTGCAATAGGCAGTTGTCAGTATAAGGTAAGGATCTAAAGCGATGCTGAGTCATCAGCTACCAAATACTCACCATGAAGCCGTCGCCTCTGAAGGTTTCGCTTTTTTATAGGCCGTGTCTGTGTCGGTATATGGCCAGTGCCAGATTACGCATCGCCCCCTAAGGGGGGGCTATTACACAACAGTCATTCGCATTTAGGCATAAAGGCTCACCGTACATAGGTGTCGCATCGCCGTCACGAGTGGACCGGGATGCGGGTACC**GTGATTTTTCTCTACAAGCGAA**TGCCGTGCATTCGCTTGCAGGTGGATTGCGATCTTTAGAACTGGAATATCTGATGATGCAAATTCCCTACGGATCATTGACTTATGACATAGGCGGGAATTTTGCATCGCATCTGTTCAAGGGACGAGCATATGTACACTGCTGCATGCCCAACCTGGACGTTCGAGACATCATGCGGCACGAAGGCCAGAAAGACAGTATTGAACTATACCTTTCTAGGCTAGAGAGAGGGGGGAAAACAGTCCCCAACTTCCAAAAGGAAGCATTTGACAGATACGCAGAAATTCCTGAAGACGCTGTCTGTCACAATACTTTCCAGACATGCG

**MIGS_ELF3_322**

**GTGATTTTTCTCTACAAGCGAA**TCTGATGATTCGATGGTGGATTCTATATCCAGCATAGATGTCTCTCCCGATGATGTTGTGGGTATATTAGGTCAAAAACGTTTCTGGAGAGCAAGGAAAGCCATTGCCAATCAACAAAGAGTATTTGCTGTTCAACTATTTGAGTTGCACAGACTGATTAAGGTTCAAAAACTTATTGCTGCATCACCGGATCTCTTGCTCGATGAGATCAGTTTTCTTGGAAAAGTTTCTGCTAAAAGCTATCCAGTGAAGAAGCTCCTTCCATCAGAATTTCTGGTAAAGCCTCCTCTACCACATGTTGTCGTCAAACAAAGGGGTGACT

**MIGS_ELF3_263**

**GTGATTTTTCTCTACAAGCGAA**TCTGATGATTCGATGGTGGATTCTATATCCAGCATAGATGTCTCTCCCGATGATGTTGTGGGTATATTAGGTCAAAAACGTTTCTGGAGAGCAAGGAAAGCCATTGCCAATCAACAAAGAGTATTTGCTGTTCAACTATTTGAGTTGCACAGACTGATTAAGGTTCAAAAACTTATTGCTGCATCACCGGATCTCTTGCTCGATGAGATCAGTTTTCTTGGAAAAGTTTCTGCTAAAAGCTATCCAGTGAAGAAGCTCCTTCC

**MIGS_ELF3_200**

**GTGATTTTTCTCTACAAGCGAA**TCTGATGATTCGATGGTGGATTCTATATCCAGCATAGATGTCTCTCCCGATGATGTTGTGGGTATATTAGGTCAAAAACGTTTCTGGAGAGCAAGGAAAGCCATTGCCAATCAACAAAGAGTATTTGCTGTTCAACTATTTGAGTTGCACAGACTGATTAAGGTTCAAAAACTTATTGCTGCATCACCGGATCTCTTGCT

**MIGS_ELF3_158**

**GTGATTTTTCTCTACAAGCGAA**TCTGATGATTCGATGGTGGATTCTATATCCAGCATAGATGTCTCTCCCGATGATGTTGTGGGTATATTAGGTCAAAAACGTTTCTGGAGAGCAAGGAAAGCCATTGCCAATCAACAAAGAGTATTTGCTGTTCAACTATTTGAGTTGCACAGACTGAT

**MIGS_ELF3_95**

**GTGATTTTTCTCTACAAGCGAA**TCTGATGATTCGATGGTGGATTCTATATCCAGCATAGATGTCTCTCCCGATGATGTTGTGGGTATATTAGGTCAAAAACGTTTCTGGAGAGCAAG

**MIGS_ELF3_C-**

TCTGATGATTCGATGGTGGATTCTATATCCAGCATAGATGTCTCTCCCGATGATGTTGTGGGTATATTAGGTCAAAAACGTTTCTGGAGAGCAAGGAAAGCCATTGCCAATCAACAAAGAGTATTTGCTGTTCAACTATTTGAGTTGCACAGACTGATTAAGGTTCAAAAACTTATTGCTGCATCACCGGATCTCTTGCTCGATGAGATCAGTTTTCTTGGAAAAGTTTCTGCTAAAAGCTATCCAGTGAAGAAGCTCCTTCCATCAGAATTTCTGGTAAAGCCTCCTCTACCACATGTTGTCGTCAAACAAAGGGGTGACT

**MIGS_TMV_A (U)**

**GTGATTTTTCTCTACAAGCGAA**TGCCGTGCATTCGCTTGCAGGTGGATTGCAATCTTTAGAACTGGAATATCAGATGATGCAAATTCCCTACGAATCATTGACTTATGACATAGACGGGAATTTTGCATCGCATCAGTTCAAGGGACGAGCATATGAACACTGCTGCATGCCCAACCAGGACGTTCGAGACATCATGCAGCACGAAGGCCAGAAAGACAATATTGAACTATACCTTTCTAAGCTAGAGAGAGGGGGGAAAAAAGTCCCCAACTTCCAAAAGGAAGCATTTGACAGATACGCAGAAATTCCTGAAGACGCTGTCTATCACAATACTTTCCAGACATACG

**MIGS_TMV_A (G)**

**GTGATTTTTCTCTACAAGCGAA**TGCCGTGCATTCGCTTGCAGGTGGATTGCCATCTTTAGAACTGGAATATCCGATGATGCAAATTCCCTACGCATCATTGACTTATGACATAGCCGGGAATTTTGCATCGCATCCGTTCAAGGGACGAGCATATGCACACTGCTGCATGCCCAACCCGGACGTTCGAGACATCATGCCGCACGAAGGCCAGAAAGACACTATTGAACTATACCTTTCTACGCTAGAGAGAGGGGGGAAAACAGTCCCCAACTTCCAAAAGGCAGCATTTGACAGATACGCAGCAATTCCTGAAGACGCTGTCTCTCACAATACTTTCCAGACATCCG

**MIGS_TMV_A_7122**

**CGACCGCGGTTTCTCTGTATAA**TGCCGTGCATTCGCTTGCAGGTGGATTGCGATCTTTAGAACTGGAATATCTGATGATGCAAATTCCCTACGGATCATTGACTTATGACATAGGCGGGAATTTTGCATCGCATCTGTTCAAGGGACGAGCATATGTACACTGCTGCATGCCCAACCTGGACGTTCGAGACATCATGCGGCACGAAGGCCAGAAAGACAGTATTGAACTATACCTTTCTAGGCTAGAGAGAGGGGGGAAAACAGTCCCCAACTTCCAAAAGGAAGCATTTGACAGATACGCAGAAATTCCTGAAGACGCTGTCTGTCACAATACTTTCCAGACATGCG

**MIGS_TMV_A_8036**

**TGGGAGGCATCGGAAAGACAAA**TGCCGTGCATTCGCTTGCAGGTGGATTGCGATCTTTAGAACTGGAATATCTGATGATGCAAATTCCCTACGGATCATTGACTTATGACATAGGCGGGAATTTTGCATCGCATCTGTTCAAGGGACGAGCATATGTACACTGCTGCATGCCCAACCTGGACGTTCGAGACATCATGCGGCACGAAGGCCAGAAAGACAGTATTGAACTATACCTTTCTAGGCTAGAGAGAGGGGGGAAAACAGTCCCCAACTTCCAAAAGGAAGCATTTGACAGATACGCAGAAATTCCTGAAGACGCTGTCTGTCACAATACTTTCCAGACATGCG

**MIGS_AG_593**

**GTGATTTTTCTCTACAAGCGAA**TCTTCTCTAGCCGTGGTCGTCTCTATGAGTACTCTAACAACAGTGTAAAAGGGACTATTGAGAGGTACAAGAAGGCAATATCGGACAATTCTAACACCGGATCGGTGGCAGAAATTAATGCACAGTATTATCAACAAGAATCAGCCAAATTGCGTCAACAAATAATCAGCATACAAAACTCCAACAGGCAATTGATGGGTGAGACGATAGGGTCAATGTCTCCCAAAGAGCTCAGGAACTTGGAAGGCAGATTAGAGAGAAGTATTACCCGAATCCGATCCAAGAAGAATGAGCTCTTATTTTCTGAAATCGACTACATGCAGAAAAGAGAAGTTGATTTGCATAACGATAACCAGATTCTTCGTGCAAAGATAGCTGAAAATGAGAGGAACAATCCGAGTATAAGTCTAATGCCAGGAGGATCTAACTACGAGCAGCTTATGCCACCACCTCAAACGCAATCTCAACCGTTTGATTCACGGAATTATTTCCAAGTCGCGGCATTGCAACCTAACAATCACCATTACTCATCCGCGGGTCGCCAAGACCAAACCGCTCTCCAGTTAGTGTAATATAGGCTGAAGGAAATGGCC

**MIGS_AG_326**

**GTGATTTTTCTCTACAAGCGAA**TCTTCTCTAGCCGTGGTCGTCTCTATGAGTACTCTAACAACAGTGTAAAAGGGACTATTGAGAGGTACAAGAAGGCAATATCGGACAATTCTAACACCGGATCGGTGGCAGAAATTAATGCACAGTATTATCAACAAGAATCAGCCAAATTGCGTCAACAAATAATCAGCATACAAAACTCCAACAGGCAATTGATGGGTGAGACGATAGGGTCAATGTCTCCCAAAGAGCTCAGGAACTTGGAAGGCAGATTAGAGAGAAGTATTACCCGAATCCGATCCAAGAAGAATGAGCTCTTATTTTCTGAAATCGACTACATGCAGAAA

**MIGS_AG_263**

**GTGATTTTTCTCTACAAGCGAA**TCTTCTCTAGCCGTGGTCGTCTCTATGAGTACTCTAACAACAGTGTAAAAGGGACTATTGAGAGGTACAAGAAGGCAATATCGGACAATTCTAACACCGGATCGGTGGCAGAAATTAATGCACAGTATTATCAACAAGAATCAGCCAAATTGCGTCAACAAATAATCAGCATACAAAACTCCAACAGGCAATTGATGGGTGAGACGATAGGGTCAATGTCTCCCAAAGAGCTCAGGAACTTGGAAGGCAGATTAGAGAGAAGT

**MIGS_AG_200**

**GTGATTTTTCTCTACAAGCGAA**TCTTCTCTAGCCGTGGTCGTCTCTATGAGTACTCTAACAACAGTGTAAAAGGGACTATTGAGAGGTACAAGAAGGCAATATCGGACAATTCTAACACCGGATCGGTGGCAGAAATTAATGCACAGTATTATCAACAAGAATCAGCCAAATTGCGTCAACAAATAATCAGCATACAAAACTCCAACAGGCAATTGATGGGT

**MIGS_AG_158**

**GTGATTTTTCTCTACAAGCGAA**TCTTCTCTAGCCGTGGTCGTCTCTATGAGTACTCTAACAACAGTGTAAAAGGGACTATTGAGAGGTACAAGAAGGCAATATCGGACAATTCTAACACCGGATCGGTGGCAGAAATTAATGCACAGTATTATCAACAAGAATCAGCCAAATTGCGTCAA

**MIGS_AG_95**

**GTGATTTTTCTCTACAAGCGAA**TCTTCTCTAGCCGTGGTCGTCTCTATGAGTACTCTAACAACAGTGTAAAAGGGACTATTGAGAGGTACAAGAAGGCAATATCGGACAATTCTAAC

**MIGS_AG_C-**

TCTTCTCTAGCCGTGGTCGTCTCTATGAGTACTCTAACAACAGTGTAAAAGGGACTATTGAGAGGTACAAGAAGGCAATATCGGACAATTCTAACACCGGATCGGTGGCAGAAATTAATGCACAGTATTATCAACAAGAATCAGCCAAATTGCGTCAACAAATAATCAGCATACAAAACTCCAACAGGCAATTGATGGGTGAGACGATAGGGTCAATGTCTCCCAAAGAGCTCAGGAACTTGGAAGGCAGATTAGAGAGAAGTATTACCCGAATCCGATCCAAGAAGAATGAGCTCTTATTTTCTGAAATCGACTACATGCAGAAAAGAGAAGTTGATTTGCATAACGATAACCAGATTCTTCGTGCAAAGATAGCTGAAAATGAGAGGAACAATCCGAGTATAAGTCTAATGCCAGGAGGATCTAACTACGAGCAGCTTATGCCACCACCTCAAACGCAATCTCAACCGTTTGATTCACGGAATTATTTCCAAGTCGCGGCATTGCAACCTAACAATCACCATTACTCATCCGCGGGTCGCCAAGACCAAACCGCTCTCCAGTTAGTGTAATATAGGCTGAAGGAAATGGCC

2) Other constructs used in this work. miRNA and miRNA* sequence is shown in Blue and Green, respectively. PDK intron is underlined.

**pmiR173**

TTCTTCTCACAAATAAACCCAAATATATCAATCTACTGTGTTGGTGATTAAGTACTTTCGCTTGCAGAGAGAAATCACAGTGGTCAAAAAAGTTGTAGTTTTCTTAAAGTCTCTTTCCTCTGTGATTCTCTGTGTAAGCGAAAGAGCTTGCTCCCTAAACTTATCTCTCTGATGATTTAATGTTAGAGATCTTCGTAAATCTATGTGTTTGATAGATCTGATGCG

**pmiR7122**

TTCTGTTATACAGAGAAACCGCGGTCGAGCTAACTGAGAAATTTGGATTTTTTTATTCCTTTTCTTCTCATTCTCAACCGCGTTTCTCCGTATAACGAAATGGTTTTAGG

**pmiR8036**

GAGAAGTTATTTGAGTGGGAGGTGTCTAAAGATAAAGTTATATTTGTTTTTTTTTCAATTCAAAGTTGAAAAATAAAAATAATTTTGTCTTTCCGATGCCTCCCATTCAC

**hpRNAi_3X**

CGGCTGGGTCTGATTGTTACGAACTGATGCGCCGAGATGGAGTAGTCATCTTCTCTGATGTAGGGGTAGGTCGCCGGTTCTCGTGCTCCTCTACATGGCGACGATAGTGGTCGGAGTCAAGCATGAAGTCCCAGGGTGTAGCGCTGCCAAATCACCAATTCGGAAGCACACTATTCGCTTATTGGCGGGCCCCCTCATCCGAAGTTCCACTTCGTTAGTAGTAAATTACTCCGGGTCTACGCAGAAAGAGTCCACTGCCGGCACAAGCAAGAGCCAACGGTATACGTCAGTGATACTCGCACTCACGATTATGACGAAATCCAGTTTGGTGTAATCACGGTGCAAACATAGAGAAACCTAACTCTCTTCCCCGGAAGGTGAGCGGCTAAGATGTCTCCAGAAAGTAACGGGAGTAGGCGAGTTATTCGAATTGTATCTAGTTTTCCACCGAGTGGCACGTTCGATACCTACTAACCTGTAGAATATTGCACCAACTGTATATACGGTTTGCAGTAGTGGATCTGTCCATTCTTCATGCCGTCGGTCCGTAGTTACGCTAAGCTAAAAATGGGCCGGGCTCATACACGCATGGAACAACTCTATGCGCTCCAGGGGTAACTCTGGTGCGTCGATGAATGACGGTCCTCTGGTAGAAGCGGGTGCTTCATGGGGTTCGTTTAAGGCTCCATGAATCTCTAAGTGGAGGGCTTGCCATGTGCTTCGTCGAGCTTAGGCTCTAATCGGAGTTGACAATCTCACCACCTAGGTCTACACTTTCTGTCGAAGATATTAGCGGAGTGGAAATGTCAGTATTCAGGCGACTGTCTAAGTGGCGCCGTTAACGGTTTTATAACGAGCAAATACGAGATCAACGAAAAGCCGAGCACCGGCCACGATAATACGAGCCCTTGGTAAGGAAATAATTATTTTCTTTTTTCCTTTTAGTATAAAATAGTTAAGTGATGTTAATTAGTATGATTATAATAATATAGTTGTTATAATTGTGAAAAAATAATTTATAAATATATTGTTTACATAAACAACATAGTAATGTAAAAAAATATGACAAGTGATGTGTAAGACGAAGAAGATAAAAGTTGAGAGTAAGTATATTATTTTTAATGAATTTGATCGAACATGTAAGATGATATACGGCCGGTAAGAGGTTCCAACTTTCACCATAATGAAATAAGATCACTACCGGGCGTATTTTTTGAGTTATCGAGATTTTCAGGAGCTAAGGAAGCTAAAATGGAGAAAAAAATCACTGGATATACCACCGTTGATATATCCCAATGGCATCGTAAAGAACATTTTGAGGCATTTCAGTCAGTTGCTCAATGTACCTATAACCAGACCGTTCAGCTGGATATTACGGCCTTTTTAAAGACCGTAAAGAAAAATAAGCACAAGTTTTATCCGGCCTTTATTCACATTCTTGCCCGCCTGATGAATGCTCATCCGGAATTCCGTATGGCAATGAAAGACGGTGAGCTGGTGATATGGGATAGTGTTCACCCTTGTTACACCGTTTTCCATGAGCAAACTGAAACGTTTTCATCGCTCTGGAGTGAATACCACGACGATTTCCGGCAGTTTCTACACATATATTCGCAAGATGTGGCGTGTTACGGTGAAAACCTGGCCTATTTCCCTAAAGGGTTTATTGAGAATATGTTTTTCGTCTCAGCCAATCCCTGGGTGAGTTTCACCAGTTTTGATTTAAACGTGGCCAATATGGACAACTTCTTCGCCCCCGTTTTCACCATGGGCAAATATTATACGCAAGGCGACAAGGTGCTGATGCCGCTGGCGATTCAGGTTCATCATGCCGTCTGTGATGGCTTCCATGTCGGCAGAATGCTTAATGAATTACAACAGTACTGCGATGAGTGGCAGGGCGGGGCGTAATCGCGTGGATCCGGCTTACTAAAAGCCAGATAACAGTATGCGTATTTGCGCGCTGATTTTTGCGGTATAAGAATATATACTGATATGTCGGTCCCATAATAGTAATTCTAGCTGGTTTGATGAATTAAATATCAATGATAAAATACTATAGTAAAAATAAGAATAAATAAATTAAAATAATATTTTTTTATGATTAATAGTTTATTATATAATTAAATATCTATACCATTACTAAATATTTTAGTTTAAAAGTTAATAAATATTTTGTTAGAAATTCCAATCTGCTTGTAATTTATCAATAAACAAAATATTAAATAACAAGCTAAAGTAACAAATAATATCAAACTAATAGAAACAGTAATCTAATGTAACAAAACATAATCTAATGCTAATATAACAAAGCGCAAGATCTATCATTTTATATAGTATTATTTTCAATCAACATTCTTATTAATTTCTAAATAATACTTGTAGTTTTATTAACTTCTAAATGGATTGACTATTAATTAAATGAATTAGTCGAACATGAATAAACAAGGTAACATGATAGATCATGTCATTGTGTTATCATTGATCTTACATTTGGATTGATTACAGTTGGTCTAGAGATTTCGTCTAGATCGTATTATCGTGGCCGGTGCTCGGCTTTTCGTTGATCTCGTATTTGCTCGTTATAAAACCGTTAACGGCGCCACTTAGACAGTCGCCTGAATACTGACATTTCCACTCCGCTAATATCTTCGACAGAAAGTGTAGACCTAGGTGGTGAGATTGTCAACTCCGATTAGAGCCTAAGCTCGACGAAGCACATGGCAAGCCCTCCACTTAGAGATTCATGGAGCCTTAAACGAACCCCATGAAGCACCCGCTTCTACCAGAGGACCGTCATTCATCGACGCACCAGAGTTACCCCTGGAGCGCATAGAGTTGTTCCATGCGTGTATGAGCCCGGCCCATTTTTAGCTTAGCGTAACTACGGACCGACGGCATGAAGAATGGACAGATCCACTACTGCAAACCGTATATACAGTTGGTGCAATATTCTACAGGTTAGTAGGTATCGAACGTGCCACTCGGTGGAAAACTAGATACAATTCGAATAACTCGCCTACTCCCGTTACTTTCTGGAGACATCTTAGCCGCTCACCTTCCGGGGAAGAGAGTTAGGTTTCTCTATGTTTGCACCGTGATTACACCAAACTGGATTTCGTCATAATCGTGAGTGCGAGTATCACTGACGTATACCGTTGGCTCTTGCTTGTGCCGGCAGTGGACTCTTTCTGCGTAGACCCGGAGTAATTTACTACTAACGAAGTGGAACTTCGGATGAGGGGGCCCGCCAATAAGCGAATAGTGTGCTTCCGAATTGGTGATTTGGCAGCGCTACACCCTGGGACTTCATGCTTGACTCCGACCACTATCGTCGCCATGTAGAGGAGCACGAGAACCGGCGACCTACCCCTACATCAGAGAAGATGACTACTCCATCTCGGCGCATCAGTTCGTAACAATCAGACCCAGCCG

**hpRNAi_6X**

CGGCTGGGTCTGATTGTTACGAACTGATGCGCCGAGATGGAGTAGTCATCTTCTCTGATGTAGGGGTAGGTCGCCGGTTCTCGTGCTCCTCTACATGGCGACGATAGTGGTCGGAGTCAAGCATGAAGTCCCAGGGTGTAGCGCTGCCAAATCACCAATTCGGAAGCACACTATTCGCTTATTGGCGGGCCCCCTCATCCGAAGTTCCACTTCGTTAGTAGTAAATTACTCCGGGTCTACGCAGAAAGAGTCCACTGCCGGCACAAGCAAGAGCCAACGGTATACGTCAGTGATACTCGCACTCACGATTATGACGAAATCCAGTTTGGTGTAATCACGGTGCAAACATAGAGAAACCTAACTCTCTTCCCCGGAAGGTGAGCGGCTAAGATGTCTCCAGAAAGTAACGGGAGTAGGCGAGTTATTCGAATTGTATCTAGTTTTCCACCGAGTGGCACGTTCGATACCTACTAACCTGTAGAATATTGCACCAACTGTATATACGGTTTGCAGTAGTGGATCTGTCCATTCTTCATGCCGTCGGTCCGTAGTTACGCTAAGCTAAAAATGGGCCGGGCTCATACACGCATGGAACAACTCTATGCGCTCCAGGGGTAACTCTGGTGCGTCGATGAATGACGGTCCTCTGGTAGAAGCGGGTGCTTCATGGGGTTCGTTTAAGGCTCCATGAATCTCTAAGTGGAGGGCTTGCCATGTGCTTCGTCGAGCTTAGGCTCTAATCGGAGTTGACAATCTCACCACCTAGGTCTACACTTTCTGTCGAAGATATTAGCGGAGTGGAAATGTCAGTATTCAGGCGACTGTCTAAGTGGCGCCGTTAACGGTTTTATAACGAGCAAATACGAGATCAACGAAAAGCCGAGCACCGGCCACGATAATCTCGGCCACTCTGAGGTGATTTCCGAACGGTCGAGGTCCCTATTGGTCGACACTACACCAGTCCATCATTACCGCACCTTCGGCGGGCGACAACATGGTTAGTTTCGGAATGTATCATGATAACTCACTGGCAGATAAGGGAGATGCGGTTTTAGCACGTCGTACGGTTCATTGCGCTATAAGAAAATAATGCATCGGTGAGCTTTGTTCAACAATAGAACGTCTTACGTCCATCCACTTCTAGCTCCGTTTAGCTAACATTGAGTAGGTGGAAATGAACATATCGCCTTTTTAGGGGTAATCCGAGTGTAACTTTCACTACGCAGTTCCTCCAAAGAACTTTGACTGATGCAGAAAGGCAATACACTCATCTCTCTCCACTTCTGTTTCGTATGTGTATGTTTTGCGTATGTCTCGAGGCGTAATATTCGACGCATAATGATGAGGTACGTCGTGATGAGCGCCCAGCAAAGAAGGGATCAGCCGGCCGGCGGGATCGGGTCGACAGCGGCTACCACCTCTATTCGTCCTTACTGCTCTGACATAGGTAAAACCTGCAAAGTCCTGTAAGGGGCCCGGCAGTGTTCGTCAACTTTCTCTAGTGTCGGAGAATTGCCAGCCGGGGGCTTAGCTGTCCGAATGTACGGATTTCCACGGGAATCTTAACTATCCCCTATGTAATACTAACAATCCGTGCCTTAAACCATGCGATGTACCGGACCGGTTGAAGGAACGTGCAGATCCTACAAAAAATAGACAATTGACCCGTGTTTGATACTTATGTCCGTACCTGCGCCCTGGTAATAAGACAAAATCCGTTAGGCCTCGAATCGAGGCGTCATGCCGAAACGAGGCTCCGGCCCACATAGTTCATAGGCACCATCTAACCGCCAACTATGTACGAGCCCTTGGTAAGGAAATAATTATTTTCTTTTTTCCTTTTAGTATAAAATAGTTAAGTGATGTTAATTAGTATGATTATAATAATATAGTTGTTATAATTGTGAAAAAATAATTTATAAATATATTGTTTACATAAACAACATAGTAATGTAAAAAAATATGACAAGTGATGTGTAAGACGAAGAAGATAAAAGTTGAGAGTAAGTATATTATTTTTAATGAATTTGATCGAACATGTAAGATGATATACGGCCGGTAAGAGGTTCCAACTTTCACCATAATGAAATAAGATCACTACCGGGCGTATTTTTTGAGTTATCGAGATTTTCAGGAGCTAAGGAAGCTAAAATGGAGAAAAAAATCACTGGATATACCACCGTTGATATATCCCAATGGCATCGTAAAGAACATTTTGAGGCATTTCAGTCAGTTGCTCAATGTACCTATAACCAGACCGTTCAGCTGGATATTACGGCCTTTTTAAAGACCGTAAAGAAAAATAAGCACAAGTTTTATCCGGCCTTTATTCACATTCTTGCCCGCCTGATGAATGCTCATCCGGAATTCCGTATGGCAATGAAAGACGGTGAGCTGGTGATATGGGATAGTGTTCACCCTTGTTACACCGTTTTCCATGAGCAAACTGAAACGTTTTCATCGCTCTGGAGTGAATACCACGACGATTTCCGGCAGTTTCTACACATATATTCGCAAGATGTGGCGTGTTACGGTGAAAACCTGGCCTATTTCCCTAAAGGGTTTATTGAGAATATGTTTTTCGTCTCAGCCAATCCCTGGGTGAGTTTCACCAGTTTTGATTTAAACGTGGCCAATATGGACAACTTCTTCGCCCCCGTTTTCACCATGGGCAAATATTATACGCAAGGCGACAAGGTGCTGATGCCGCTGGCGATTCAGGTTCATCATGCCGTCTGTGATGGCTTCCATGTCGGCAGAATGCTTAATGAATTACAACAGTACTGCGATGAGTGGCAGGGCGGGGCGTAATCGCGTGGATCCGGCTTACTAAAAGCCAGATAACAGTATGCGTATTTGCGCGCTGATTTTTGCGGTATAAGAATATATACTGATATGTCGGTCCCATAATAGTAATTCTAGCTGGTTTGATGAATTAAATATCAATGATAAAATACTATAGTAAAAATAAGAATAAATAAATTAAAATAATATTTTTTTATGATTAATAGTTTATTATATAATTAAATATCTATACCATTACTAAATATTTTAGTTTAAAAGTTAATAAATATTTTGTTAGAAATTCCAATCTGCTTGTAATTTATCAATAAACAAAATATTAAATAACAAGCTAAAGTAACAAATAATATCAAACTAATAGAAACAGTAATCTAATGTAACAAAACATAATCTAATGCTAATATAACAAAGCGCAAGATCTATCATTTTATATAGTATTATTTTCAATCAACATTCTTATTAATTTCTAAATAATACTTGTAGTTTTATTAACTTCTAAATGGATTGACTATTAATTAAATGAATTAGTCGAACATGAATAAACAAGGTAACATGATAGATCATGTCATTGTGTTATCATTGATCTTACATTTGGATTGATTACAGTTGGTCTAGAGATTTCGTCTAGATCGTACATAGTTGGCGGTTAGATGGTGCCTATGAACTATGTGGGCCGGAGCCTCGTTTCGGCATGACGCCTCGATTCGAGGCCTAACGGATTTTGTCTTATTACCAGGGCGCAGGTACGGACATAAGTATCAAACACGGGTCAATTGTCTATTTTTTGTAGGATCTGCACGTTCCTTCAACCGGTCCGGTACATCGCATGGTTTAAGGCACGGATTGTTAGTATTACATAGGGGATAGTTAAGATTCCCGTGGAAATCCGTACATTCGGACAGCTAAGCCCCCGGCTGGCAATTCTCCGACACTAGAGAAAGTTGACGAACACTGCCGGGCCCCTTACAGGACTTTGCAGGTTTTACCTATGTCAGAGCAGTAAGGACGAATAGAGGTGGTAGCCGCTGTCGACCCGATCCCGCCGGCCGGCTGATCCCTTCTTTGCTGGGCGCTCATCACGACGTACCTCATCATTATGCGTCGAATATTACGCCTCGAGACATACGCAAAACATACACATACGAAACAGAAGTGGAGAGAGATGAGTGTATTGCCTTTCTGCATCAGTCAAAGTTCTTTGGAGGAACTGCGTAGTGAAAGTTACACTCGGATTACCCCTAAAAAGGCGATATGTTCATTTCCACCTACTCAATGTTAGCTAAACGGAGCTAGAAGTGGATGGACGTAAGACGTTCTATTGTTGAACAAAGCTCACCGATGCATTATTTTCTTATAGCGCAATGAACCGTACGACGTGCTAAAACCGCATCTCCCTTATCTGCCAGTGAGTTATCATGATACATTCCGAAACTAACCATGTTGTCGCCCGCCGAAGGTGCGGTAATGATGGACTGGTGTAGTGTCGACCAATAGGGACCTCGACCGTTCGGAAATCACCTCAGAGTGGCCGAGATTATCGTGGCCGGTGCTCGGCTTTTCGTTGATCTCGTATTTGCTCGTTATAAAACCGTTAACGGCGCCACTTAGACAGTCGCCTGAATACTGACATTTCCACTCCGCTAATATCTTCGACAGAAAGTGTAGACCTAGGTGGTGAGATTGTCAACTCCGATTAGAGCCTAAGCTCGACGAAGCACATGGCAAGCCCTCCACTTAGAGATTCATGGAGCCTTAAACGAACCCCATGAAGCACCCGCTTCTACCAGAGGACCGTCATTCATCGACGCACCAGAGTTACCCCTGGAGCGCATAGAGTTGTTCCATGCGTGTATGAGCCCGGCCCATTTTTAGCTTAGCGTAACTACGGACCGACGGCATGAAGAATGGACAGATCCACTACTGCAAACCGTATATACAGTTGGTGCAATATTCTACAGGTTAGTAGGTATCGAACGTGCCACTCGGTGGAAAACTAGATACAATTCGAATAACTCGCCTACTCCCGTTACTTTCTGGAGACATCTTAGCCGCTCACCTTCCGGGGAAGAGAGTTAGGTTTCTCTATGTTTGCACCGTGATTACACCAAACTGGATTTCGTCATAATCGTGAGTGCGAGTATCACTGACGTATACCGTTGGCTCTTGCTTGTGCCGGCAGTGGACTCTTTCTGCGTAGACCCGGAGTAATTTACTACTAACGAAGTGGAACTTCGGATGAGGGGGCCCGCCAATAAGCGAATAGTGTGCTTCCGAATTGGTGATTTGGCAGCGCTACACCCTGGGACTTCATGCTTGACTCCGACCACTATCGTCGCCATGTAGAGGAGCACGAGAACCGGCGACCTACCCCTACATCAGAGAAGATGACTACTCCATCTCGGCGCATCAGTTCGTAACAATCAGACCCAGCCG

**hpRNAi_9X**

CGGCTGGGTCTGATTGTTACGAACTGATGCGCCGAGATGGAGTAGTCATCTTCTCTGATGTAGGGGTAGGTCGCCGGTTCTCGTGCTCCTCTACATGGCGACGATAGTGGTCGGAGTCAAGCATGAAGTCCCAGGGTGTAGCGCTGCCAAATCACCAATTCGGAAGCACACTATTCGCTTATTGGCGGGCCCCCTCATCCGAAGTTCCACTTCGTTAGTAGTAAATTACTCCGGGTCTACGCAGAAAGAGTCCACTGCCGGCACAAGCAAGAGCCAACGGTATACGTCAGTGATACTCGCACTCACGATTATGACGAAATCCAGTTTGGTGTAATCACGGTGCAAACATAGAGAAACCTAACTCTCTTCCCCGGAAGGTGAGCGGCTAAGATGTCTCCAGAAAGTAACGGGAGTAGGCGAGTTATTCGAATTGTATCTAGTTTTCCACCGAGTGGCACGTTCGATACCTACTAACCTGTAGAATATTGCACCAACTGTATATACGGTTTGCAGTAGTGGATCTGTCCATTCTTCATGCCGTCGGTCCGTAGTTACGCTAAGCTAAAAATGGGCCGGGCTCATACACGCATGGAACAACTCTATGCGCTCCAGGGGTAACTCTGGTGCGTCGATGAATGACGGTCCTCTGGTAGAAGCGGGTGCTTCATGGGGTTCGTTTAAGGCTCCATGAATCTCTAAGTGGAGGGCTTGCCATGTGCTTCGTCGAGCTTAGGCTCTAATCGGAGTTGACAATCTCACCACCTAGGTCTACACTTTCTGTCGAAGATATTAGCGGAGTGGAAATGTCAGTATTCAGGCGACTGTCTAAGTGGCGCCGTTAACGGTTTTATAACGAGCAAATACGAGATCAACGAAAAGCCGAGCACCGGCCACGATAATCTCGGCCACTCTGAGGTGATTTCCGAACGGTCGAGGTCCCTATTGGTCGACACTACACCAGTCCATCATTACCGCACCTTCGGCGGGCGACAACATGGTTAGTTTCGGAATGTATCATGATAACTCACTGGCAGATAAGGGAGATGCGGTTTTAGCACGTCGTACGGTTCATTGCGCTATAAGAAAATAATGCATCGGTGAGCTTTGTTCAACAATAGAACGTCTTACGTCCATCCACTTCTAGCTCCGTTTAGCTAACATTGAGTAGGTGGAAATGAACATATCGCCTTTTTAGGGGTAATCCGAGTGTAACTTTCACTACGCAGTTCCTCCAAAGAACTTTGACTGATGCAGAAAGGCAATACACTCATCTCTCTCCACTTCTGTTTCGTATGTGTATGTTTTGCGTATGTCTCGAGGCGTAATATTCGACGCATAATGATGAGGTACGTCGTGATGAGCGCCCAGCAAAGAAGGGATCAGCCGGCCGGCGGGATCGGGTCGACAGCGGCTACCACCTCTATTCGTCCTTACTGCTCTGACATAGGTAAAACCTGCAAAGTCCTGTAAGGGGCCCGGCAGTGTTCGTCAACTTTCTCTAGTGTCGGAGAATTGCCAGCCGGGGGCTTAGCTGTCCGAATGTACGGATTTCCACGGGAATCTTAACTATCCCCTATGTAATACTAACAATCCGTGCCTTAAACCATGCGATGTACCGGACCGGTTGAAGGAACGTGCAGATCCTACAAAAAATAGACAATTGACCCGTGTTTGATACTTATGTCCGTACCTGCGCCCTGGTAATAAGACAAAATCCGTTAGGCCTCGAATCGAGGCGTCATGCCGAAACGAGGCTCCGGCCCACATAGTTCATAGGCACCATCTAACCGCCAACTATGTGGATTGAATGCGACGGGTGGTTTCGTTGTTAGTGGATGCACTGTTAGACCGGAATCGAAACTTTGGGTGTTAGGACACAGGATGGTGACTAACTAGTCACGCGAGCCATCACCTCGAGCTAAAGAGAATCAGCTTCGTTTTCCTGCTGACAGGTTCGTTTTTTTAGGCTACTTCAATGGGATCTCGCCCGGGCTGCACACCATACGTCCTGTAATCATGAAGCAGCGGAGCGCTTCTGCGGCGCGTGACCTTCAAGTGCTGGAGTATGTAATAAGGCTACTGTATGAGCGCGAGGAGAGGGGCTGTGCCCACAAAGCTCAATTTCCTAGTAATCGTATGCAACCTCACGAGAATCGTGCTTTACTTGTCACAATCGTAGCCTCGCGGGGCGATAAGATGGTTCCATATCATTATCCGGAAACTTCTACGACCCAGGGTAATTACAAACAACATGCAAAAAGAGAGAGTACGAGCGGACGTACGAGACCGCACAACGGGGTTCCGACTGAGTGAGGGGGGCAACGTTACACATTTCCGGCCGAATAAGGTCCCAGAAGTTAGTGTATCGTAGTTGGTCGTACGGGTTGCGAGTATAATGGCTGCGTGGCCGCCCTAGGACCGCAGACGACGGAATGTCTTCTTGCGTCAACAAATCAACTTGACCTACGTTCGATCTGCTGCCCCTGCGAAGATTTCCTTGGACATGTGACTCCTCTAAAAGAATATACCCTCAAAAGATAGTGTGTCAGAGCGGCACCGTCATCTTCCGTAGAGTCGGGCCGATGCCAACTCGAGATCGGGAAATACTTGACCTTTACCCTGATTATCCGAACGGTCCCTCTGATCAGGGCAAAAGTCCAGAGACTTTATTTATTCAGAGGTTTCGGACCCTGCGGTAGACGATCTAGACGAAATCTCTAGACCAACTGTAATCAATCCAAATGTAAGATCAATGATAACACAATGACATGATCTATCATGTTACCTTGTTTATTCATGTTCGACTAATTCATTTAATTAATAGTCAATCCATTTAGAAGTTAATAAAACTACAAGTATTATTTAGAAATTAATGAGAATGTTGATTGAAAATAATACTATATAAAATTGATAGATCTTGCGCTTTGTTATATTAGCATTAGATTATGTTTTGTTACATTAGATTACTGTTTCTATTAGTTTGATATTATTTGTTACTTTAGCTTGTTATTTAATATTTTGTTTATTGATAAATTACAAGCAGATTGGAATTTCTAACAAAATATTTATTAACTTTTAAACTAAAATATTTAGTAATGGTATAGATATTTAATTATATAATAAACTATTAATCATAAAAAAATATTATTTTAATTTATTTATTCTTATTTTTACTATAGTATTTTATCATTGATATTTAATTCATCAAACCAGCTAGAATTACTATTATGGGCCGACATATCAGTATATATTCTTATACCGCAAAAATCAGCGCGCAAATACGCATACTGTTATCTGGCTTTTAGTAAGCCGGATCCACGCGGCGTTTACGCCCCCCCTGCCACTCATCGCAGTACTGTTGTAATTCATTAAGCATTCTGCCGACATGGAAGCCATCACAAACGGCATGATGAACCTGAATCGCCAGCGGCATCAGCACCTTGTCGCCTTGCGTATAATATTTGCCCATGGTGAAAACGGGGGCGAAGAAGTTGTCCATATTGGCCACGTTTAAATCAAAACTGGTGAAACTCACCCAGGGATTGGCTGAGACGAAAAACATATTCTCAATAAACCCTTTAGGGAAATAGGCCAGGTTTTCACCGTAACACGCCACATCTTGCGAATATATGTGTAGAAACTGCCGGAAATCGTCGTGGTATTCACTCCAGAGCGATGAAAACGTTTCAGTTTGCTCATGGAAAACGGTGTAACAAGGGTGAACACTATCCCATATCACCAGCTCACCGTCTTTCATTGCCATACGGAATTCCGGATGAGCATTCATCAGGCGGGCAAGAATGTGAATAAAGGCCGGATAAAACTTGTGCTTATTTTTCTTTACGGTCTTTAAAAAGGCCGTAATATCCAGCTGAACGGTCTGGTTATAGGTACATTGAACAACTGACTGAAATGCCTCAAAATGTTCTTTACGATGCCATTGGGATATATCAACGGTGGTATATCCAGTGATTTTTTTCTCCATTTTAGCTTCCTTAGCTCCTGAAAATCTCGATAACTCAAAAAATACGCCCGGTAGTGATCTTATTTCATTATGGTGAAAGTTGGAACCTCTTCGGCCGTATATCATCTTACATGTTCGATCAAATTCATTAAAAATAATATACTTACTCTCAACTTTTATCTTCTTCGTCTTACACATCACTTGTCATATTTTTTTACATTACTATGTTGTTTATGTAAACAATATATTTATAAATTATTTTTTCACAATTATAACAACTATATTATTATAATCATACTAATTAACATCACTTAACTATTTTATACTAAAAGGAAAAAAGAAAATAATTATTTCCTTACCAAGGGCTCGTCTACCGCAGGGTCCGAAACCTCTGAATAAATAAAGTCTCTGGACTTTTGCCCTGATCAGAGGGACCGTTCGGATAATCAGGGTAAAGGTCAAGTATTTCCCGATCTCGAGTTGGCATCGGCCCGACTCTACGGAAGATGACGGTGCCGCTCTGACACACTATCTTTTGAGGGTATATTCTTTTAGAGGAGTCACATGTCCAAGGAAATCTTCGCAGGGGCAGCAGATCGAACGTAGGTCAAGTTGATTTGTTGACGCAAGAAGACATTCCGTCGTCTGCGGTCCTAGGGCGGCCACGCAGCCATTATACTCGCAACCCGTACGACCAACTACGATACACTAACTTCTGGGACCTTATTCGGCCGGAAATGTGTAACGTTGCCCCCCTCACTCAGTCGGAACCCCGTTGTGCGGTCTCGTACGTCCGCTCGTACTCTCTCTTTTTGCATGTTGTTTGTAATTACCCTGGGTCGTAGAAGTTTCCGGATAATGATATGGAACCATCTTATCGCCCCGCGAGGCTACGATTGTGACAAGTAAAGCACGATTCTCGTGAGGTTGCATACGATTACTAGGAAATTGAGCTTTGTGGGCACAGCCCCTCTCCTCGCGCTCATACAGTAGCCTTATTACATACTCCAGCACTTGAAGGTCACGCGCCGCAGAAGCGCTCCGCTGCTTCATGATTACAGGACGTATGGTGTGCAGCCCGGGCGAGATCCCATTGAAGTAGCCTAAAAAAACGAACCTGTCAGCAGGAAAACGAAGCTGATTCTCTTTAGCTCGAGGTGATGGCTCGCGTGACTAGTTAGTCACCATCCTGTGTCCTAACACCCAAAGTTTCGATTCCGGTCTAACAGTGCATCCACTAACAACGAAACCACCCGTCGCATTCAATCCACATAGTTGGCGGTTAGATGGTGCCTATGAACTATGTGGGCCGGAGCCTCGTTTCGGCATGACGCCTCGATTCGAGGCCTAACGGATTTTGTCTTATTACCAGGGCGCAGGTACGGACATAAGTATCAAACACGGGTCAATTGTCTATTTTTTGTAGGATCTGCACGTTCCTTCAACCGGTCCGGTACATCGCATGGTTTAAGGCACGGATTGTTAGTATTACATAGGGGATAGTTAAGATTCCCGTGGAAATCCGTACATTCGGACAGCTAAGCCCCCGGCTGGCAATTCTCCGACACTAGAGAAAGTTGACGAACACTGCCGGGCCCCTTACAGGACTTTGCAGGTTTTACCTATGTCAGAGCAGTAAGGACGAATAGAGGTGGTAGCCGCTGTCGACCCGATCCCGCCGGCCGGCTGATCCCTTCTTTGCTGGGCGCTCATCACGACGTACCTCATCATTATGCGTCGAATATTACGCCTCGAGACATACGCAAAACATACACATACGAAACAGAAGTGGAGAGAGATGAGTGTATTGCCTTTCTGCATCAGTCAAAGTTCTTTGGAGGAACTGCGTAGTGAAAGTTACACTCGGATTACCCCTAAAAAGGCGATATGTTCATTTCCACCTACTCAATGTTAGCTAAACGGAGCTAGAAGTGGATGGACGTAAGACGTTCTATTGTTGAACAAAGCTCACCGATGCATTATTTTCTTATAGCGCAATGAACCGTACGACGTGCTAAAACCGCATCTCCCTTATCTGCCAGTGAGTTATCATGATACATTCCGAAACTAACCATGTTGTCGCCCGCCGAAGGTGCGGTAATGATGGACTGGTGTAGTGTCGACCAATAGGGACCTCGACCGTTCGGAAATCACCTCAGAGTGGCCGAGATTATCGTGGCCGGTGCTCGGCTTTTCGTTGATCTCGTATTTGCTCGTTATAAAACCGTTAACGGCGCCACTTAGACAGTCGCCTGAATACTGACATTTCCACTCCGCTAATATCTTCGACAGAAAGTGTAGACCTAGGTGGTGAGATTGTCAACTCCGATTAGAGCCTAAGCTCGACGAAGCACATGGCAAGCCCTCCACTTAGAGATTCATGGAGCCTTAAACGAACCCCATGAAGCACCCGCTTCTACCAGAGGACCGTCATTCATCGACGCACCAGAGTTACCCCTGGAGCGCATAGAGTTGTTCCATGCGTGTATGAGCCCGGCCCATTTTTAGCTTAGCGTAACTACGGACCGACGGCATGAAGAATGGACAGATCCACTACTGCAAACCGTATATACAGTTGGTGCAATATTCTACAGGTTAGTAGGTATCGAACGTGCCACTCGGTGGAAAACTAGATACAATTCGAATAACTCGCCTACTCCCGTTACTTTCTGGAGACATCTTAGCCGCTCACCTTCCGGGGAAGAGAGTTAGGTTTCTCTATGTTTGCACCGTGATTACACCAAACTGGATTTCGTCATAATCGTGAGTGCGAGTATCACTGACGTATACCGTTGGCTCTTGCTTGTGCCGGCAGTGGACTCTTTCTGCGTAGACCCGGAGTAATTTACTACTAACGAAGTGGAACTTCGGATGAGGGGGCCCGCCAATAAGCGAATAGTGTGCTTCCGAATTGGTGATTTGGCAGCGCTACACCCTGGGACTTCATGCTTGACTCCGACCACTATCGTCGCCATGTAGAGGAGCACGAGAACCGGCGACCTACCCCTACATCAGAGAAGATGACTACTCCATCTCGGCGCATCAGTTCGTAACAATCAGACCCAGCCG

**hpRNAi_12X**

CGGCTGGGTCTGATTGTTACGAACTGATGCGCCGAGATGGAGTAGTCATCTTCTCTGATGTAGGGGTAGGTCGCCGGTTCTCGTGCTCCTCTACATGGCGACGATAGTGGTCGGAGTCAAGCATGAAGTCCCAGGGTGTAGCGCTGCCAAATCACCAATTCGGAAGCACACTATTCGCTTATTGGCGGGCCCCCTCATCCGAAGTTCCACTTCGTTAGTAGTAAATTACTCCGGGTCTACGCAGAAAGAGTCCACTGCCGGCACAAGCAAGAGCCAACGGTATACGTCAGTGATACTCGCACTCACGATTATGACGAAATCCAGTTTGGTGTAATCACGGTGCAAACATAGAGAAACCTAACTCTCTTCCCCGGAAGGTGAGCGGCTAAGATGTCTCCAGAAAGTAACGGGAGTAGGCGAGTTATTCGAATTGTATCTAGTTTTCCACCGAGTGGCACGTTCGATACCTACTAACCTGTAGAATATTGCACCAACTGTATATACGGTTTGCAGTAGTGGATCTGTCCATTCTTCATGCCGTCGGTCCGTAGTTACGCTAAGCTAAAAATGGGCCGGGCTCATACACGCATGGAACAACTCTATGCGCTCCAGGGGTAACTCTGGTGCGTCGATGAATGACGGTCCTCTGGTAGAAGCGGGTGCTTCATGGGGTTCGTTTAAGGCTCCATGAATCTCTAAGTGGAGGGCTTGCCATGTGCTTCGTCGAGCTTAGGCTCTAATCGGAGTTGACAATCTCACCACCTAGGTCTACACTTTCTGTCGAAGATATTAGCGGAGTGGAAATGTCAGTATTCAGGCGACTGTCTAAGTGGCGCCGTTAACGGTTTTATAACGAGCAAATACGAGATCAACGAAAAGCCGAGCACCGGCCACGATAATCTCGGCCACTCTGAGGTGATTTCCGAACGGTCGAGGTCCCTATTGGTCGACACTACACCAGTCCATCATTACCGCACCTTCGGCGGGCGACAACATGGTTAGTTTCGGAATGTATCATGATAACTCACTGGCAGATAAGGGAGATGCGGTTTTAGCACGTCGTACGGTTCATTGCGCTATAAGAAAATAATGCATCGGTGAGCTTTGTTCAACAATAGAACGTCTTACGTCCATCCACTTCTAGCTCCGTTTAGCTAACATTGAGTAGGTGGAAATGAACATATCGCCTTTTTAGGGGTAATCCGAGTGTAACTTTCACTACGCAGTTCCTCCAAAGAACTTTGACTGATGCAGAAAGGCAATACACTCATCTCTCTCCACTTCTGTTTCGTATGTGTATGTTTTGCGTATGTCTCGAGGCGTAATATTCGACGCATAATGATGAGGTACGTCGTGATGAGCGCCCAGCAAAGAAGGGATCAGCCGGCCGGCGGGATCGGGTCGACAGCGGCTACCACCTCTATTCGTCCTTACTGCTCTGACATAGGTAAAACCTGCAAAGTCCTGTAAGGGGCCCGGCAGTGTTCGTCAACTTTCTCTAGTGTCGGAGAATTGCCAGCCGGGGGCTTAGCTGTCCGAATGTACGGATTTCCACGGGAATCTTAACTATCCCCTATGTAATACTAACAATCCGTGCCTTAAACCATGCGATGTACCGGACCGGTTGAAGGAACGTGCAGATCCTACAAAAAATAGACAATTGACCCGTGTTTGATACTTATGTCCGTACCTGCGCCCTGGTAATAAGACAAAATCCGTTAGGCCTCGAATCGAGGCGTCATGCCGAAACGAGGCTCCGGCCCACATAGTTCATAGGCACCATCTAACCGCCAACTATGTGGATTGAATGCGACGGGTGGTTTCGTTGTTAGTGGATGCACTGTTAGACCGGAATCGAAACTTTGGGTGTTAGGACACAGGATGGTGACTAACTAGTCACGCGAGCCATCACCTCGAGCTAAAGAGAATCAGCTTCGTTTTCCTGCTGACAGGTTCGTTTTTTTAGGCTACTTCAATGGGATCTCGCCCGGGCTGCACACCATACGTCCTGTAATCATGAAGCAGCGGAGCGCTTCTGCGGCGCGTGACCTTCAAGTGCTGGAGTATGTAATAAGGCTACTGTATGAGCGCGAGGAGAGGGGCTGTGCCCACAAAGCTCAATTTCCTAGTAATCGTATGCAACCTCACGAGAATCGTGCTTTACTTGTCACAATCGTAGCCTCGCGGGGCGATAAGATGGTTCCATATCATTATCCGGAAACTTCTACGACCCAGGGTAATTACAAACAACATGCAAAAAGAGAGAGTACGAGCGGACGTACGAGACCGCACAACGGGGTTCCGACTGAGTGAGGGGGGCAACGTTACACATTTCCGGCCGAATAAGGTCCCAGAAGTTAGTGTATCGTAGTTGGTCGTACGGGTTGCGAGTATAATGGCTGCGTGGCCGCCCTAGGACCGCAGACGACGGAATGTCTTCTTGCGTCAACAAATCAACTTGACCTACGTTCGATCTGCTGCCCCTGCGAAGATTTCCTTGGACATGTGACTCCTCTAAAAGAATATACCCTCAAAAGATAGTGTGTCAGAGCGGCACCGTCATCTTCCGTAGAGTCGGGCCGATGCCAACTCGAGATCGGGAAATACTTGACCTTTACCCTGATTATCCGAACGGTCCCTCTGATCAGGGCAAAAGTCCAGAGACTTTATTTATTCAGAGGTTTCGGACCCTGCGGTAGGGTGATTTTTCGCATATTTGCGCCCACAGGAAAGTACCTGACTACGTTCTACGTACGTCCCCATTAACCATCACGGAAACTTCTAACAATGAAAGTCGATGATTGCGATGGTGATTGAGAGGGGCTATAAGATTCGGAAGTGCAGCATTTTGGGAGTGGCCCGAAGCCAGCCCGATTTTGTAAATGATGGATTTGGTCTATACCGTGCCCCGCACTATGAAGCACCGGTGCCGCAAAATTAGATTACTTTCGTCGTTCTGGCAGCGCCCCGTTCAAGAAATTGTCTAGTTAGAGGTTTCAAGGAGAGAATGCTTCGGGGTTCCGGGAGAGCCGAGTACGCGATAACCCTCGACCGGGTGTTTGTAGCGTCGCACTTCAACCTTAGTACATCGAGCGCGGACCGACCTGCCCCAAAAACCCACGGCGCGGGAGAACCCTACAGCCCCATCGTTTACAGTCGCGTACCCGGCTATTGCCTGTTGACTTTGGGGTCTAGGTGCGACAGATGGACGTACCTACTCAGGCCCGAGTACGGGATAATATAATGGTCCCGCATCCGTCGCTATAAAAAATGTCGCCTTCTGGGCCACCAAGGCAAGACATCAATGGACAACCTTCCGTTATGTTTAGAGAAGGGGGGGTTGCAATAGGCAGTTGTCAGTATAAGGTAAGGATCTAAAGCGATGCTGAGTCATCAGCTACCAAATACTCACCATGAAGCCGTCGCCTCTGAAGGTTTCGCTTTTTTATAGGCCGTGTCTGTGTCGGTATATGGCCAGTGCCAGATTACGCATCGCCCCCTAAGGGGGGGCTATTACACAACAGTCATTCGCATTTAGGCATAAATTGGCTCACCGTACATAGGTGTCGCATCGCCGTCACGAGTGGACCGGGATGCGACGAGCCCTTGGTAAGGAAATAATTATTTTCTTTTTTCCTTTTAGTATAAAATAGTTAAGTGATGTTAATTAGTATGATTATAATAATATAGTTGTTATAATTGTGAAAAAATAATTTATAAATATATTGTTTACATAAACAACATAGTAATGTAAAAAAATATGACAAGTGATGTGTAAGACGAAGAAGATAAAAGTTGAGAGTAAGTATATTATTTTTAATGAATTTGATCGAACATGTAAGATGATATACGGCCGAAGAGGTTCCAACTTTCACCATAATGAAATAAGATCACTACCGGGCGTATTTTTTGAGTTATCGAGATTTTCAGGAGCTAAGGAAGCTAAAATGGAGAAAAAAATCACTGGATATACCACCGTTGATATATCCCAATGGCATCGTAAAGAACATTTTGAGGCATTTCAGTCAGTTGTTCAATGTACCTATAACCAGACCGTTCAGCTGGATATTACGGCCTTTTTAAAGACCGTAAAGAAAAATAAGCACAAGTTTTATCCGGCCTTTATTCACATTCTTGCCCGCCTGATGAATGCTCATCCGGAATTCCGTATGGCAATGAAAGACGGTGAGCTGGTGATATGGGATAGTGTTCACCCTTGTTACACCGTTTTCCATGAGCAAACTGAAACGTTTTCATCGCTCTGGAGTGAATACCACGACGATTTCCGGCAGTTTCTACACATATATTCGCAAGATGTGGCGTGTTACGGTGAAAACCTGGCCTATTTCCCTAAAGGGTTTATTGAGAATATGTTTTTCGTCTCAGCCAATCCCTGGGTGAGTTTCACCAGTTTTGATTTAAACGTGGCCAATATGGACAACTTCTTCGCCCCCGTTTTCACCATGGGCAAATATTATACGCAAGGCGACAAGGTGCTGATGCCGCTGGCGATTCAGGTTCATCATGCCGTTTGTGATGGCTTCCATGTCGGCAGAATGCTTAATGAATTACAACAGTACTGCGATGAGTGGCAGGGGGGGCGTAAACGCCGCGTGGATCCGGCTTACTAAAAGCCAGATAACAGTATGCGTATTTGCGCGCTGATTTTTGCGGTATAAGAATATATACTGATATGTCGGCCCATAATAGTAATTCTAGCTGGTTTGATGAATTAAATATCAATGATAAAATACTATAGTAAAAATAAGAATAAATAAATTAAAATAATATTTTTTTATGATTAATAGTTTATTATATAATTAAATATCTATACCATTACTAAATATTTTAGTTTAAAAGTTAATAAATATTTTGTTAGAAATTCCAATCTGCTTGTAATTTATCAATAAACAAAATATTAAATAACAAGCTAAAGTAACAAATAATATCAAACTAATAGAAACAGTAATCTAATGTAACAAAACATAATCTAATGCTAATATAACAAAGCGCAAGATCTATCAATTTTATATAGTATTATTTTCAATCAACATTCTCATTAATTTCTAAATAATACTTGTAGTTTTATTAACTTCTAAATGGATTGACTATTAATTAAATGAATTAGTCGAACATGAATAAACAAGGTAACATGATAGATCATGTCATTGTGTTATCATTGATCTTACATTTGGATTGATTACAGTTGGTCTAGAGATTTCGTCTAGATCGTCGCATCCCGGTCCACTCGTGACGGCGATGCGACACCTATGTACGGTGAGCCAATTTATGCCTAAATGCGAATGACTGTTGTGTAATAGCCCCCCCTTAGGGGGCGATGCGTAATCTGGCACTGGCCATATACCGACACAGACACGGCCTATAAAAAAGCGAAACCTTCAGAGGCGACGGCTTCATGGTGAGTATTTGGTAGCTGATGACTCAGCATCGCTTTAGATCCTTACCTTATACTGACAACTGCCTATTGCAACCCCCCCTTCTCTAAACATAACGGAAGGTTGTCCATTGATGTCTTGCCTTGGTGGCCCAGAAGGCGACATTTTTTATAGCGACGGATGCGGGACCATTATATTATCCCGTACTCGGGCCTGAGTAGGTACGTCCATCTGTCGCACCTAGACCCCAAAGTCAACAGGCAATAGCCGGGTACGCGACTGTAAACGATGGGGCTGTAGGGTTCTCCCGCGCCGTGGGTTTTTGGGGCAGGTCGGTCCGCGCTCGATGTACTAAGGTTGAAGTGCGACGCTACAAACACCCGGTCGAGGGTTATCGCGTACTCGGCTCTCCCGGAACCCCGAAGCATTCTCTCCTTGAAACCTCTAACTAGACAATTTCTTGAACGGGGCGCTGCCAGAACGACGAAAGTAATCTAATTTTGCGGCACCGGTGCTTCATAGTGCGGGGCACGGTATAGACCAAATCCATCATTTACAAAATCGGGCTGGCTTCGGGCCACTCCCAAAATGCTGCACTTCCGAATCTTATAGCCCCTCTCAATCACCATCGCAATCATCGACTTTCATTGTTAGAAGTTTCCGTGATGGTTAATGGGGACGTACGTAGAACGTAGTCAGGTACTTTCCTGTGGGCGCAAATATGCGAAAAATCACCCTACCGCAGGGTCCGAAACCTCTGAATAAATAAAGTCTCTGGACTTTTGCCCTGATCAGAGGGACCGTTCGGATAATCAGGGTAAAGGTCAAGTATTTCCCGATCTCGAGTTGGCATCGGCCCGACTCTACGGAAGATGACGGTGCCGCTCTGACACACTATCTTTTGAGGGTATATTCTTTTAGAGGAGTCACATGTCCAAGGAAATCTTCGCAGGGGCAGCAGATCGAACGTAGGTCAAGTTGATTTGTTGACGCAAGAAGACATTCCGTCGTCTGCGGTCCTAGGGAGGCCACGCAGCCATTATACTCGCAACCCGTACGACCAACTACGATACACTAACTTCTGGGACCTTATTCGGCCGGAAATGTGTAACGTTGCCCCCCTCACTCAGTCGGAACCCCGTTGTGCGGTCTCGTACGTCCGCTCGTACTCTCTCTTTTTGCATGTTGTTTGTAATTACCCTGGGTCGTAGAAGTTTCCGGATAATGATATGGAACCATCTTATCGCCCCGCGAGGCTACGATTGTGACAAGTAAAGCACGATTCTCGTGAGGTTGCATACGATTACTAGGAAATTGAGCTTTGTGGGCACAGCCCCTCTCCTCGCGCTCATACAGTAGCCTTATTACATACTCCAGCACTTGAAGGTCACGCGCCGCAGAAGCGCTCCGCTGCTTCATGATTACAGGACGTATGGTGTGCAGCCCGGGCGAGATCCCATTGAAGTAGCCTAAAAAAACGAACCTGTCAGCAGGAAAACGAAGCTGATTCTCTTTAGCTCGAGGTGATGGCTCGCGTGACTAGTTAGTCACCATCCTGTGTCCTAACACCCAAAGTTTCGATTCCGGTCTAACAGTGCATCCACTAACAACGAAACCACCCGTCGCATTCAATCCACATAGTTGGCGGTTAGATGGTGCCTATGAACTATGTGGGCCGGAGCCTCGTTTCGGCATGACGCCTCGATTCGAGGCCTAACGGATTTTGTCTTATTACCAGGGCGCAGGTACGGACATAAGTATCAAACACGGGTCAATTGTCTATTTTTTGTAGGATCTGCACGTTCCTTCAACCGGTCCGGTACATCGCATGGTTTAAGGCACGGATTGTTAGTATTACATAGGGGATAGTTAAGATTCCCGTGGAAATCCGTACATTCGGACAGCTAAGCCCCCGGCTGGCAATTCTCCGACACTAGAGAAAGTTGACGAACACTGCCGGGCCCCTTACAGGACTTTGCAGGTTTTACCTATGTCAGAGCAGTAAGGACGAATAGAGGTGGTAGCCGCTGTCGACCCGATCCCGCCGGCCGGCTGATCCCTTCTTTGCTGGGCGCTCATCACGACGTACCTCATCATTATGCGTCGAATATTACGCCTCGAGACATACGCAAAACATACACATACGAAACAGAAGTGGAGAGAGATGAGTGTATTGCCTTTCTGCATCAGTCAAAGTTCTTTGGAGGAACTGCGTAGTGAAAGTTACACTCGGATTACCCCTAAAAAGGCGATATGTTCATTTCCACCTACTCAATGTTAGCTAAACGGAGCTAGAAGTGGATGGACGTAAGACGTTCTATTGTTGAACAAAGCTCACCGATGCATTATTTTCTTATAGCGCAATGAACCGTACGACGTGCTAAAACCGCATCTCCCTTATCTGCCAGTGAGTTATCATGATACATTCCGAAACTAACCATGTTGTCGCCCGCCGAAGGTGCGGTAATGATGGACTGGTTTAGTGTCGACCAATAGGGACCTCGACCGTTCGGAAATCACCTCAGAGTGGCCGAGATTATCGTGGCCGGTGCTCGGCTTTTCGTTGATCTCGTATTTGCTCGTTATAAAACCGTTAACGGCGCCACTTAGACAGTCGCCTGAATACTGACATTTCCACTCCGCTAATATCTTCGACAGAAAGTGTAGACCTAGGTGGTGAGATTGTCAACTCCGATTAGAGCCTAAGCTCGACGAAGCACATGGCAAGCCCTCCACTTAGAGATTCATGGAGCCTTAAACGAACCCCATGAAGCACCCGCTTCTACCAGAGGACCGTCATTCATCGACGCACCAGAGTTACCCCTGGAGCGCATAGAGTTGTTCCATGCGTGTATGAGCCCGGCCCATTTTTAGCTTAGCGTAACTACGGACCGACGGCATGAAGAATGGACAGATCCACTACTGCAAACCGTATATACAGTTGGTGCAATATTCTACAGGTTAGTAGGTATCGAACGTGCCACTCGGTGGAAAACTAGATACAATTCGAATAACTCGCCTACTCCCGTTACTTTCTGGAGACATCTTAGCCGCTCACCTTCCGGGGAAGAGAGTTAGGTTTCTCTATGTTTGCACCGTGATTACACCAAACTGGATTTCGTCATAATCGTGAGTGCGAGTATCACTGACGTATACCGTTGGCTCTTGCTTGTGCCGGCAGTGGACTCTTTCTGCGTAGACCCGGAGTAATTTACTACTAACGAAGTGGAACTTCGGATGAGGGGGCCCGCCAATAAGCGAATAGTGTGCTTCCGAATTGGTGATTTGGCAGCGCTACACCCTGGGACTTCATGCTTGACTCCGACCACTATCGTCGCCATGTAGAGGAGCACGAGAACCGGCGACCTACCCCTACATCAGAGAAGATGACTACTCCATCTCGGCGCATCAGTTCGTAACAATCAGACCCAGCCG

**hpRNAi_15X**

CGGCTGGGTCTGATTGTTACGAACTGATGCGCCGAGATGGAGTAGTCATCTTCTCTGATGTAGGGGTAGGTCGCCGGTTCTCGTGCTCCTCTACATGGCGACGATAGTGGTCGGAGTCAAGCATGAAGTCCCAGGGTGTAGCGCTGCCAAATCACCAATTCGGAAGCACACTATTCGCTTATTGGCGGGCCCCCTCATCCGAAGTTCCACTTCGTTAGTAGTAAATTACTCCGGGTCTACGCAGAAAGAGTCCACTGCCGGCACAAGCAAGAGCCAACGGTATACGTCAGTGATACTCGCACTCACGATTATGACGAAATCCAGTTTGGTGTAATCACGGTGCAAACATAGAGAAACCTAACTCTCTTCCCCGGAAGGTGAGCGGCTAAGATGTCTCCAGAAAGTAACGGGAGTAGGCGAGTTATTCGAATTGTATCTAGTTTTCCACCGAGTGGCACGTTCGATACCTACTAACCTGTAGAATATTGCACCAACTGTATATACGGTTTGCAGTAGTGGATCTGTCCATTCTTCATGCCGTCGGTCCGTAGTTACGCTAAGCTAAAAATGGGCCGGGCTCATACACGCATGGAACAACTCTATGCGCTCCAGGGGTAACTCTGGTGCGTCGATGAATGACGGTCCTCTGGTAGAAGCGGGTGCTTCATGGGGTTCGTTTAAGGCTCCATGAATCTCTAAGTGGAGGGCTTGCCATGTGCTTCGTCGAGCTTAGGCTCTAATCGGAGTTGACAATCTCACCACCTAGGTCTACACTTTCTGTCGAAGATATTAGCGGAGTGGAAATGTCAGTATTCAGGCGACTGTCTAAGTGGCGCCGTTAACGGTTTTATAACGAGCAAATACGAGATCAACGAAAAGCCGAGCACCGGCCACGATAATCTCGGCCACTCTGAGGTGATTTCCGAACGGTCGAGGTCCCTATTGGTCGACACTACACCAGTCCATCATTACCGCACCTTCGGCGGGCGACAACATGGTTAGTTTCGGAATGTATCATGATAACTCACTGGCAGATAAGGGAGATGCGGTTTTAGCACGTCGTACGGTTCATTGCGCTATAAGAAAATAATGCATCGGTGAGCTTTGTTCAACAATAGAACGTCTTACGTCCATCCACTTCTAGCTCCGTTTAGCTAACATTGAGTAGGTGGAAATGAACATATCGCCTTTTTAGGGGTAATCCGAGTGTAACTTTCACTACGCAGTTCCTCCAAAGAACTTTGACTGATGCAGAAAGGCAATACACTCATCTCTCTCCACTTCTGTTTCGTATGTGTATGTTTTGCGTATGTCTCGAGGCGTAATATTCGACGCATAATGATGAGGTACGTCGTGATGAGCGCCCAGCAAAGAAGGGATCAGCCGGCCGGCGGGATCGGGTCGACAGCGGCTACCACCTCTATTCGTCCTTACTGCTCTGACATAGGTAAAACCTGCAAAGTCCTGTAAGGGGCCCGGCAGTGTTCGTCAACTTTCTCTAGTGTCGGAGAATTGCCAGCCGGGGGCTTAGCTGTCCGAATGTACGGATTTCCACGGGAATCTTAACTATCCCCTATGTAATACTAACAATCCGTGCCTTAAACCATGCGATGTACCGGACCGGTTGAAGGAACGTGCAGATCCTACAAAAAATAGACAATTGACCCGTGTTTGATACTTATGTCCGTACCTGCGCCCTGGTAATAAGACAAAATCCGTTAGGCCTCGAATCGAGGCGTCATGCCGAAACGAGGCTCCGGCCCACATAGTTCATAGGCACCATCTAACCGCCAACTATGTGGATTGAATGCGACGGGTGGTTTCGTTGTTAGTGGATGCACTGTTAGACCGGAATCGAAACTTTGGGTGTTAGGACACAGGATGGTGACTAACTAGTCACGCGAGCCATCACCTCGAGCTAAAGAGAATCAGCTTCGTTTTCCTGCTGACAGGTTCGTTTTTTTAGGCTACTTCAATGGGATCTCGCCCGGGCTGCACACCATACGTCCTGTAATCATGAAGCAGCGGAGCGCTTCTGCGGCGCGTGACCTTCAAGTGCTGGAGTATGTAATAAGGCTACTGTATGAGCGCGAGGAGAGGGGCTGTGCCCACAAAGCTCAATTTCCTAGTAATCGTATGCAACCTCACGAGAATCGTGCTTTACTTGTCACAATCGTAGCCTCGCGGGGCGATAAGATGGTTCCATATCATTATCCGGAAACTTCTACGACCCAGGGTAATTACAAACAACATGCAAAAAGAGAGAGTACGAGCGGACGTACGAGACCGCACAACGGGGTTCCGACTGAGTGAGGGGGGCAACGTTACACATTTCCGGCCGAATAAGGTCCCAGAAGTTAGTGTATCGTAGTTGGTCGTACGGGTTGCGAGTATAATGGCTGCGTGGCCGCCCTAGGACCGCAGACGACGGAATGTCTTCTTGCGTCAACAAATCAACTTGACCTACGTTCGATCTGCTGCCCCTGCGAAGATTTCCTTGGACATGTGACTCCTCTAAAAGAATATACCCTCAAAAGATAGTGTGTCAGAGCGGCACCGTCATCTTCCGTAGAGTCGGGCCGATGCCAACTCGAGATCGGGAAATACTTGACCTTTACCCTGATTATCCGAACGGTCCCTCTGATCAGGGCAAAAGTCCAGAGACTTTATTTATTCAGAGGTTTCGGACCCTGCGGTAGGGTGATTTTTCGCATATTTGCGCCCACAGGAAAGTACCTGACTACGTTCTACGTACGTCCCCATTAACCATCACGGAAACTTCTAACAATGAAAGTCGATGATTGCGATGGTGATTGAGAGGGGCTATAAGATTCGGAAGTGCAGCATTTTGGGAGTGGCCCGAAGCCAGCCCGATTTTGTAAATGATGGATTTGGTCTATACCGTGCCCCGCACTATGAAGCACCGGTGCCGCAAAATTAGATTACTTTCGTCGTTCTGGCAGCGCCCCGTTCAAGAAATTGTCTAGTTAGAGGTTTCAAGGAGAGAATGCTTCGGGGTTCCGGGAGAGCCGAGTACGCGATAACCCTCGACCGGGTGTTTGTAGCGTCGCACTTCAACCTTAGTACATCGAGCGCGGACCGACCTGCCCCAAAAACCCACGGCGCGGGAGAACCCTACAGCCCCATCGTTTACAGTCGCGTACCCGGCTATTGCCTGTTGACTTTGGGGTCTAGGTGCGACAGATGGACGTACCTACTCAGGCCCGAGTACGGGATAATATAATGGTCCCGCATCCGTCGCTATAAAAAATGTCGCCTTCTGGGCCACCAAGGCAAGACATCAATGGACAACCTTCCGTTATGTTTAGAGAAGGGGGGGTTGCAATAGGCAGTTGTCAGTATAAGGTAAGGATCTAAAGCGATGCTGAGTCATCAGCTACCAAATACTCACCATGAAGCCGTCGCCTCTGAAGGTTTCGCTTTTTTATAGGCCGTGTCTGTGTCGGTATATGGCCAGTGCCAGATTACGCATCGCCCCCTAAGGGGGGGCTATTACACAACAGTCATTCGCATTTAGGCATAAATTGGCTCACCGTACATAGGTGTCGCATCGCCGTCACGAGTGGACCGGGATGCGATTGTGGAAGGTGTAGCTACAGAACTCGATCACCAAGAGTGCACAACTGAATCCATCAGTCGTTGCGTTAGCTATTTCCAACCACGCGATTCGCGCGTAACACCGTTGTGAACGTACGGGGCATGACGTGAAGACCACACGGTGGATAATGAAAGGCATCACGTCCTCTCCGAATCTCGAAATTAACAAGTACCTCTTGAAAGCGATGCTCCCTCAGCGCTTGCGTTATCGCAACGGGAAGTGCCAGCGCTACGATCCGCCACTGTAAGTCCCCGCTCCGTGATATATGGAATTATCTTTCATCCTCTCCCACTACTGAGCAGCTCCTGCGAGCCCATTGTAGTCTAGGCTTTATGCGGGCTTGATGCGACCGTTTCAACCGGTTGGATACTTGTTAGGCCTGTCGTCTGCAACCCGCACGCGCGTGGGATGGGGGCAATGTCAGTTAGGGGGTCCTTCCTCGACCGACGGTCACGCTCTGTTATCCCTAATAACACAAGGAATTATGTGCGGCTTTTGCCGGGATGCTCTATTTACGTTATCGTTCCCACTAAGACGCTCATAGGGTCGCTACAATGGGGGAAGGGACTTATTTGCTTCGCGTCTTCTTGCCACGCGGATTCAATAGTTAGATGGTGGTCGTGCCCCCTTACAGCACAGCCCGTAAGATATAAACGAAGCCGGCGTCTCGAAGGATCCGTACTACCAATTGTGTTTACGATTTGGTACAGCAGCTAGTAGTTGGAGGGAGAGGGATGGACAAACGTTATATTACTTTCATCCGCTATATGTTGCATCTTGATGGCGTAAAATGAGCATACCTCAACCCGGAGCCGCCGCCAACTCTGTAAACCTTACGCTGAGTACCCCCGCGGCCCTCCACCTGTTCATAATCGCTCAACGATCTAGACGAAATCTCTAGACCAACTGTAATCAATCCAAATGTAAGATCAATGATAACACAATGACATGATCTATCATGTTACCTTGTTTATTCATGTTCGACTAATTCATTTAATTAATAGTCAATCCATTTAGAAGTTAATAAAACTACAAGTATTATTTAGAAATTAATGAGAATGTTGATTGAAAATAATACTATATAAAATTGATAGATCTTGCGCTTTGTTATATTAGCATTAGATTATGTTTTGTTACATTAGATTACTGTTTCTATTAGTTTGATATTATTTGTTACTTTAGCTTGTTATTTAATATTTTGTTTATTGATAAATTACAAGCAGATTGGAATTTCTAACAAAATATTTATTAACTTTTAAACTAAAATATTTAGTAATGGTATAGATATTTAATTATATAATAAACTATTAATCATAAAAAAATATTATTTTAATTTATTTATTCTTATTTTTACTATAGTATTTTATCATTGATATTTAATTCATCAAACCAGCTAGAATTACTATTATGGGCCGACATATCAGTATATATTCTTATACCGCAAAAATCAGCGCGCAAATACGCATACTGTTATCTGGCTTTTAGTAAGCCGGATCCACGCGGCGTTTACGCCCCCCCTGCCACTCATCGCAGTACTGTTGTAATTCATTAAGCATTCTGCCGACATGGAAGCCATCACAAACGGCATGATGAACCTGAATCGCCAGCGGCATCAGCACCTTGTCGCCTTGCGTATAATATTTGCCCATGGTGAAAACGGGGGCGAAGAAGTTGTCCATATTGGCCACGTTTAAATCAAAACTGGTGAAACTCACCCAGGGATTGGCTGAGACGAAAAACATATTCTCAATAAACCCTTTAGGGAAATAGGCCAGGTTTTCACCGTAACACGCCACATCTTGCGAATATATGTGTAGAAACTGCCGGAAATCGTCGTGGTATTCACTCCAGAGCGATGAAAACGTTTCAGTTTGCTCATGGAAAACGGTGTAACAAGGGTGAACACTATCCCATATCACCAGCTCACCGTCTTTCATTGCCATACGGAATTCCGGATGAGCATTCATCAGGCGGGCAAGAATGTGAATAAAGGCCGGATAAAACTTGTGCTTATTTTTCTTTACGGTCTTTAAAAAGGCCGTAATATCCAGCTGAACGGTCTGGTTATAGGTACATTGAACAACTGACTGAAATGCCTCAAAATGTTCTTTACGATGCCATTGGGATATATCAACGGTGGTATATCCAGTGATTTTTTTCTCCATTTTAGCTTCCTTAGCTCCTGAAAATCTCGATAACTCAAAAAATACGCCCGGTAGTGATCTTATTTCATTATGGTGAAAGTTGGAACCTCTTCGGCCGTATATCATCTTACATGTTCGATCAAATTCATTAAAAATAATATACTTACTCTCAACTTTTATCTTCTTCGTCTTACACATCACTTGTCATATTTTTTTACATTACTATGTTGTTTATGTAAACAATATATTTATAAATTATTTTTTCACAATTATAACAACTATATTATTATAATCATACTAATTAACATCACTTAACTATTTTATACTAAAAGGAAAAAAGAAAATAATTATTTCCTTACCAAGGGCTCGTTGAGCGATTATGAACAGGTGGAGGGCCGCGGGGGTACTCAGCGTAAGGTTTACAGAGTTGGCGGCGGCTCCGGGTTGAGGTATGCTCATTTTACGCCATCAAGATGCAACATATAGCGGATGAAAGTAATATAACGTTTGTCCATCCCTCTCCCTCCAACTACTAGCTGCTGTACCAAATCGTAAACACAATTGGTAGTACGGATCCTTCGAGACGCCGGCTTCGTTTATATCTTACGGGCTGTGCTGTAAGGGGGCACGACCACCATCTAACTATTGAATCCGCGTGGCAAGAAGACGCGAAGCAAATAAGTCCCTTCCCCCATTGTAGCGACCCTATGAGCGTCTTAGTGGGAACGATAACGTAAATAGAGCATCCCGGCAAAAGCCGCACATAATTCCTTGTGTTATTAGGGATAACAGAGCGTGACCGTCGGTCGAGGAAGGACCCCCTAACTGACATTGCCCCCATCCCACGCGCGTGCGGGTTGCAGACGACAGGCCTAACAAGTATCCAACCGGTTGAAACGGTCGCATCAAGCCCGCATAAAGCCTAGACTACAATGGGCTCGCAGGAGCTGCTCAGTAGTGGGAGAGGATGAAAGATAATTCCATATATCACGGAGCGGGGACTTACAGTGGCGGATCGTAGCGCTGGCACTTCCCGTTGCGATAACGCAAGCGCTGAGGGAGCATCGCTTTCAAGAGGTACTTGTTAATTTCGAGATTCGGAGAGGACGTGATGCCTTTCATTATCCACCGTGTGGTCTTCACGTCATGCCCCGTACGTTCACAACGGTGTTACGCGCGAATCGCGTGGTTGGAAATAGCTAACGCAACGACTGATGGATTCAGTTGTGCACTCTTGGTGATCGAGTTCTGTAGCTACACCTTCCACAATCGCATCCCGGTCCACTCGTGACGGCGATGCGACACCTATGTACGGTGAGCCAATTTATGCCTAAATGCGAATGACTGTTGTGTAATAGCCCCCCCTTAGGGGGCGATGCGTAATCTGGCACTGGCCATATACCGACACAGACACGGCCTATAAAAAAGCGAAACCTTCAGAGGCGACGGCTTCATGGTGAGTATTTGGTAGCTGATGACTCAGCATCGCTTTAGATCCTTACCTTATACTGACAACTGCCTATTGCAACCCCCCCTTCTCTAAACATAACGGAAGGTTGTCCATTGATGTCTTGCCTTGGTGGCCCAGAAGGCGACATTTTTTATAGCGACGGATGCGGGACCATTATATTATCCCGTACTCGGGCCTGAGTAGGTACGTCCATCTGTCGCACCTAGACCCCAAAGTCAACAGGCAATAGCCGGGTACGCGACTGTAAACGATGGGGCTGTAGGGTTCTCCCGCGCCGTGGGTTTTTGGGGCAGGTCGGTCCGCGCTCGATGTACTAAGGTTGAAGTGCGACGCTACAAACACCCGGTCGAGGGTTATCGCGTACTCGGCTCTCCCGGAACCCCGAAGCATTCTCTCCTTGAAACCTCTAACTAGACAATTTCTTGAACGGGGCGCTGCCAGAACGACGAAAGTAATCTAATTTTGCGGCACCGGTGCTTCATAGTGCGGGGCACGGTATAGACCAAATCCATCATTTACAAAATCGGGCTGGCTTCGGGCCACTCCCAAAATGCTGCACTTCCGAATCTTATAGCCCCTCTCAATCACCATCGCAATCATCGACTTTCATTGTTAGAAGTTTCCGTGATGGTTAATGGGGACGTACGTAGAACGTAGTCAGGTACTTTCCTGTGGGCGCAAATATGCGAAAAATCACCCTACCGCAGGGTCCGAAACCTCTGAATAAATAAAGTCTCTGGACTTTTGCCCTGATCAGAGGGACCGTTCGGATAATCAGGGTAAAGGTCAAGTATTTCCCGATCTCGAGTTGGCATCGGCCCGACTCTACGGAAGATGACGGTGCCGCTCTGACACACTATCTTTTGAGGGTATATTCTTTTAGAGGAGTCACATGTCCAAGGAAATCTTCGCAGGGGCAGCAGATCGAACGTAGGTCAAGTTGATTTGTTGACGCAAGAAGACATTCCGTCGTCTGCGGTCCTAGGGCGGCCACGCAGCCATTATACTCGCAACCCGTACGACCAACTACGATACACTAACTTCTGGGACCTTATTCGGCCGGAAATGTGTAACGTTGCCCCCCTCACTCAGTCGGAACCCCGTTGTGCGGTCTCGTACGTCCGCTCGTACTCTCTCTTTTTGCATGTTGTTTGTAATTACCCTGGGTCGTAGAAGTTTCCGGATAATGATATGGAACCATCTTATCGCCCCGCGAGGCTACGATTGTGACAAGTAAAGCACGATTCTCGTGAGGTTGCATACGATTACTAGGAAATTGAGCTTTGTGGGCACAGCCCCTCTCCTCGCGCTCATACAGTAGCCTTATTACATACTCCAGCACTTGAAGGTCACGCGCCGCAGAAGCGCTCCGCTGCTTCATGATTACAGGACGTATGGTGTGCAGCCCGGGCGAGATCCCATTGAAGTAGCCTAAAAAAACGAACCTGTCAGCAGGAAAACGAAGCTGATTCTCTTTAGCTCGAGGTGATGGCTCGCGTGACTAGTTAGTCACCATCCTGTGTCCTAACACCCAAAGTTTCGATTCCGGTCTAACAGTGCATCCACTAACAACGAAACCACCCGTCGCATTCAATCCACATAGTTGGCGGTTAGATGGTGCCTATGAACTATGTGGGCCGGAGCCTCGTTTCGGCATGACGCCTCGATTCGAGGCCTAACGGATTTTGTCTTATTACCAGGGCGCAGGTACGGACATAAGTATCAAACACGGGTCAATTGTCTATTTTTTGTAGGATCTGCACGTTCCTTCAACCGGTCCGGTACATCGCATGGTTTAAGGCACGGATTGTTAGTATTACATAGGGGATAGTTAAGATTCCCGTGGAAATCCGTACATTCGGACAGCTAAGCCCCCGGCTGGCAATTCTCCGACACTAGAGAAAGTTGACGAACACTGCCGGGCCCCTTACAGGACTTTGCAGGTTTTACCTATGTCAGAGCAGTAAGGACGAATAGAGGTGGTAGCCGCTGTCGACCCGATCCCGCCGGCCGGCTGATCCCTTCTTTGCTGGGCGCTCATCACGACGTACCTCATCATTATGCGTCGAATATTACGCCTCGAGACATACGCAAAACATACACATACGAAACAGAAGTGGAGAGAGATGAGTGTATTGCCTTTCTGCATCAGTCAAAGTTCTTTGGAGGAACTGCGTAGTGAAAGTTACACTCGGATTACCCCTAAAAAGGCGATATGTTCATTTCCACCTACTCAATGTTAGCTAAACGGAGCTAGAAGTGGATGGACGTAAGACGTTCTATTGTTGAACAAAGCTCACCGATGCATTATTTTCTTATAGCGCAATGAACCGTACGACGTGCTAAAACCGCATCTCCCTTATCTGCCAGTGAGTTATCATGATACATTCCAAAACTAACCATGTTGTCGCCCGCCGAAGGTGCGGTAATGATGGACTGGTGTAGTGTCGACCAATAGGGACCTCGACCGTTCGGAAATCACCTCAGAGTGGCCGAGATTATCGTGGCCGGTGCTCGGCTTTTCGTTGATCTCGTATTTGCTCGTTATAAAACCGTTAACGGCGCCACTTAGACAGTCGCCTGAATACTGACATTTCCACTCCGCTAATATCTTCGACAGAAAGTGTAGACCTAGGTGGTGAGATTGTCAACTCCGATTAGAGCCTAAGCTCGACGAAGCACATGGCAAGCCCTCCACTTAGAGATTCATGGAGCCTTAAACGAACCCCATGAAGCACCCGCTTCTACCAGAGGACCGTCATTCATCGACGCACCAGAGTTACCCCTGGAGCGCATAGAGTTGTTCCATGCGTGTATGAGCCCGGCCCATTTTTAGCTTAGCGTAACTACGGACCGACGGCATGAAGAATGGACAGATCCACTACTGCAAACCGTATATACAGTTGGTGCAATATTCTACAGGTTAGTAGGTATCGAACGTGCCACTCGGTGGAAAACTAGATACAATTCGAATAACTCGCCTACTCCCGTTACTTTCTGGAGACATCTTAGCCGCTCACCTTCCGGGGAAGAGAGTTAGGTTTCTCTATGTTTGCACCGTGATTACACCAAACTGGATTTCGTCATAATCGTGAGTGCGAGTATCACTGACGTATACCGTTGGCTCTTGCTTGTGCCGGCAGTGGACTCTTTCTGCGTAGACCCGGAGTAATTTACTACTAACGAAGTGGAACTTCGGATGAGGGGGCCCGCCAATAAGCGAATAGTGTGCTTCCGAATTGGTGATTTGGCAGCGCTACACCCTGGGACTTCATGCTTGACTCCGACCACTATCGTCGCCATGTAGAGGAGCACGAGAACCGGCGACCTACCCCTACATCAGAGAAGATGACTACTCCATCTCGGCGCATCAGTTCGTAACAATCAGACCCAGCCG

3) Supplementary material and methods

***Generation of hpRNAi vectors:*** the random sequences used to create the modules in pMIGS_3X to pMIGS_15X were amplified and fused in a single chimeric sequence (CS) using overlapping PCR. The resulting CS was cloned into pJB-GG Big using Golden Gate cloning. BsaI sites for Golden Gate were added by PCR. Primers used are given below.

List of primers used to create the hpRNAi_3X to hpRNAi_15X. The BsaI site is shown in **bold underlined.** Whenever applicable, both of the primers used (for fusing two sequences or to add the BsaI site) are given.

| **MIGS template** | **Sequence** | |
| --- | --- | --- |
|  | **Fwd** | **Rev** |
| Module 1 | ACCA**GGTCTC**AGGAGCGGCTGGGTCTGATTGTTACG | AATCGTGAGTGCGAGTATCACTGACG |
| Module 2 | TGATACTCGCACTCACGATTATGACG | GGAGCGCATAGAGTTGTTCCATGCGTG |
| Module 3 | GGAACAACTCTATGCGCTCCAGGGGTAAC | AGTGGCCGAGATTATCGTGGCCGGTGC |
| Module 3 |  | ACCA**GGTCTC**ATCGTATTATCGTGGCCGGTGCTCG |
| Module 4 | CCACGATAATCTCGGCCACTCTGAGGTG | ACACTCGGATTACCCCTAAAAAGGCGATATG |
| Module 5 | TTTAGGGGTAATCCGAGTGTAACTTTC | CTCCGACACTAGAGAAAGTTGACGAAC |
| Module 6 | AACTTTCTCTAGTGTCGGAGAATTGCC | CATTCAATCCACATAGTTGGCGGTTAG |
| Module 6 |  | ACCA**GGTCTC**ATCGTACATAGTTGGCGGTTAGATGG |
| Module 7 | CCAACTATGTGGATTGAATGCGACGGGTG | GGGCACAGCCCCTCTCCTCGCGCTCATAC |
| Module 8 | CGAGGAGAGGGGCTGTGCCCACAAAGC | CGGCCACGCAGCCATTATACTCGCAACC |
| Module 9 | GTATAATGGCTGCGTGGCCGCCCTAGG | AAAAATCACCCTACCGCAGGGTCCGAAACC |
| Module 9 |  | ACCA**GGTCTC**ATCGTCTACCGCAGGGTCCGAAACC |
| Module 10 | CCTGCGGTAGGGTGATTTTTCGCATATTTGC | ATTCTCTCCTTGAAACCTCTAACTAGAC |
| Module 11 | AGAGGTTTCAAGGAGAGAATGCTTCGG | TCCATTGATGTCTTGCCTTGGTGGCCCAG |
| Module 12 | CAAGGCAAGACATCAATGGACAACCTTCC | CTTCCACAATCGCATCCCGGTCCACTC |
| Module 12 |  | ACCA **GGTCTC** ATCGT CGCATCCCGGTCCACTCGTG |
| Module 13 | CCGGGATGCGATTGTGGAAGGTGTAGC | GGAGAGGATGAAAGATAATTCCATATATCACG |
| Module 14 | AATTATCTTTCATCCTCTCCCACTACTG | AAGAAGACGCGAAGCAAATAAGTCCC |
| Module 15 | TATTTGCTTCGCGTCTTCTTGCCACGC | ACCA**GGTCTC**ATCGTTGAGCGATTATGAACAGGTGG |
|  |  |  |

***Quantification of gel bands using ImageJ (FIJI):*** Using the rectangular selection tool, lanes encompassing the bands were created. The plot lanes function was then used to create a profile plot showing the band intensity as peaks. After creating a baseline using the line tool, we measured the area of each peak using the wand tool.

***Small RNA size distribution and phasing analyses:*** Libraries for pMIGS_6X and pMIGS_9X (3 replicates each) were prepared using the QIASeq miRNA library kit (Qiagen, Hilden, Germany) and sequenced on a NovaSeq X Plus using a 10B flow cell. Library preparation and sequencing conditions for pMIGS_15X, pMIGS_TMV_A, pMIGS_TMV_A_(U) and pMIGS_TMV_A_(G) are given in the main text (materials and methods section). Since the samples on the NovaSeq for pMIGS_6X and pMIGS_9X were run using a dark cycle recipe, where the first sequencing cycle is not recorded, a single unknown base (N) was added at the 5′ end of the reads after the quality filtering to account for this and accurately reflect the true read length in downstream analyses. A custom python script (Python version 3.7.12) was used to generate grouped bar charts showing the distribution of read sizes for genomic regions of interest, to verify that the data exhibited the expected small RNA size profile, with characteristic enrichment at 21, 22, and 24 nucleotides. After converting BAM files to bed files using bedtools v2.30.0, the percentage of mapped reads was plotted as relative distance from the centre. The radial plot provides a merge phasing profile for the reads mapping on both the positive and negative strand, by accounting for how phasing is offset by 2 positions upstream on the negative strand.
