# Supplementary material for: MicroRNA‐Induced Gene Silencing (MIGS): A Tool for Multi‐Gene Silencing and Targeting Viruses in Plants
Source: Plant Biotechnol J. 2025 Oct 6;24(3):973–87. doi: 10.1111/pbi.70401 (PMC12946496; doi:10.1111/pbi.70401)
Supplement: Supplementary file 3 — Table S1: Mean read count of 21 and 22 nt sRNA reads against the pMIGS_15X construct. Mean read count for interval regions of interest, calculated using GATK. [file PBI-24-973-s001.docx]

**Table S1.** Mean read count of 21 and 22 nt sRNA reads against the pMIGS_15X construct. Mean read count for interval regions of interest, calculated using GATK.

| **MIGS_15x construct** | **miR173 +** | | **miR173 -** | | **Fold difference (21 + 22 nt)** |
| --- | --- | --- | --- | --- | --- |
|  | **21 nt** | **22 nt** | **21 nt** | **22 nt** |  |
| MIGS_15x module 1 | 27341.66 | 8223.15 | 381.53 | 210.98 | 60.0 |
| MIGS_15x module 2 | 39984.80 | 6451.63 | 795.64 | 375.80 | 39.6 |
| MIGS_15x module 3 | 18020.62 | 6080.70 | 1125.02 | 589.68 | 14.1 |
| MIGS_15x module 4 | 11081.25 | 3655.30 | 1162.98 | 614.69 | 8.3 |
| MIGS_15x module 5 | 4230.04 | 1413.04 | 797.91 | 443.89 | 4.5 |
| MIGS_15x module 6 | 2655.44 | 1167.87 | 310.62 | 193.07 | 7.6 |
| MIGS_15x module 7 | 5040.05 | 822.86 | 336.37 | 225.16 | 10.4 |
| MIGS_15x module 8 | 1850.18 | 512.58 | 281.90 | 203.11 | 4.9 |
| MIGS_15x module 9 | 1321.56 | 458.34 | 177.48 | 128.04 | 5.8 |
| MIGS_15x module 10 | 1142.13 | 318.90 | 186.68 | 162.68 | 4.2 |
| MIGS_15x module 11 | 848.65 | 352.85 | 83.95 | 64.14 | 8.1 |
| MIGS_15x module 12 | 505.91 | 257.42 | 120.56 | 90.25 | 3.6 |
| MIGS_15x module 13 | 1047.53 | 174.12 | 132.97 | 92.54 | 5.4 |
| MIGS_15x module 14 | 331.64 | 122.32 | 75.79 | 58.43 | 3.4 |
| MIGS_15x module 15 | 110.18 | 62.73 | 60.58 | 54.26 | 1.5 |
