# Supplementary material for: MicroRNA‐Induced Gene Silencing (MIGS): A Tool for Multi‐Gene Silencing and Targeting Viruses in Plants
Source: Plant Biotechnol J. 2025 Oct 6;24(3):973–87. doi: 10.1111/pbi.70401 (PMC12946496; doi:10.1111/pbi.70401)
Supplement: Supplementary file 4 — Table S2: List of primers and probes used in this work. Restriction enzyme sites are shown underlined and the target sequence for the MIGS initiator is in bold (miR173 unless otherwise indicated). [file PBI-24-973-s002.docx]

**Table S2.** List of primers and probes used in this work. Restriction enzyme sites are shown underlined and the target sequence for the MIGS initiator is in **bold** (miR173 unless otherwise indicated).

| Primer name | sequence | target | | Observation | | |  |
| --- | --- | --- | --- | --- | --- | --- | --- |
| *pMIGS_ELF3 series* | | | | | |  |  |
| FQ-367 | AAACCCGGG**GTGATTTTTCTCTACAAGCGAA**TCTGATGATTCGATGGTGG | ELF3 | | XmaI site | | |  |
| FQ-368 | TTTGCGGCCGCAGTCACCCCTTTGTTTGAC | ELF3 | | NotI site | | |  |
| FQ-369 | TTTGCGGCCGCGGAAGGAGCTTCTTCACTGG | ELF3 | | NotI site | | |  |
| FQ-370 | TTTGCGGCCGCAGCAAGAGATCCGGTGATGC | ELF3 | | NotI site | | |  |
| FQ-371 | TTTGCGGCCGCATCAGTCTGTGCAACTCAAA | ELF3 | | NotI site | | |  |
| FQ-372 | TTTGCGGCCGCCTTGCTCTCCAGAAACGT | ELF3 | | NotI site | | |  |
| FQ-373 | AAACCCGGGTCTGATGATTCGATGGTGG | ELF3 | | XmaI site | | |  |
| *pMIGS_AG series* | | | | | |  |  |
| FQ-360 | AAACCCGGG**GTGATTTTTCTCTACAAGCGAA**TCTTCTCTAGCCGTGGTCG | AG | | XmaI site | |  |  |
| FQ-361 | TTTGCGGCCGCGGCCATTTCCTTCAGCCTA | AG | | NotI site | |  |  |
| FQ-362 | TTTGCGGCCGCTTTCTGCATGTAGTCGATTT | AG | | NotI site | |  |  |
| FQ-363 | TTTGCGGCCGCACTTCTCTCTAATCTGCCTTC | AG | | NotI site | |  |  |
| FQ-364 | TTTGCGGCCGCACCCATCAATTGCCTGTTG | AG | | NotI site | |  |  |
| FQ-365 | TTTGCGGCCGCTTGACGCAATTTGGCTGATT | AG | | NotI site | |  |  |
| FQ-366 | TTTGCGGCCGCGTTAGAATTGTCCGATATTG | AG | | NotI site | |  |  |
| FQ-374 | AAACCCGGGTCTTCTCTAGCCGTGGTCG | AG | | XmaI site | |  |  |
| *pMIGS_TMV series* |  |  | |  | |  |  |
| FQ-410 | AAACCCGGG**GTGATTTTTCTCTACAAGCGAA**TGCCGTGCATTCGCTTGCAG | TMV_A (Rep) | | XmaI site | |  |  |
| FQ-411 | TTTGCGGCCGCCGCATGTCTGGAAAGTATTG | TMV_A (Rep) | | NotI site | |  |  |
| FQ-412 | AAACCCGGG**GTGATTTTTCTCTACAAGCGAA**CAGTTTTACTATGATAAGTG | TMV_B (RdRP) | | XmaI site | |  |  |
| FQ-413 | TTTGCGGCCGCAGCAAATAACTATCAAAAAAC | TMV_B (RdRP) | | NotI site | |  |  |
| FQ-414 | AAACCCGGG**GTGATTTTTCTCTACAAGCGAA**GCTCTAGTTGTTAAAGGAA | TMV_C (MP) | | XmaI site | |  |  |
| FQ-415 | TTTGCGGCCGCGTGTAGTAAGATCCGAGAGTG | TMV_C (MP) | | NotI site | |  |  |
| FQ-416 | CGCATGTCTGGAAAGTATTG | TMV_A (Rep) | |  | |  |  |
| FQ-417 | CAATACTTTCCAGACATGCG**GTGATTTTTCTCTACAAGCGAA**CAGTTTTACTATGATAAGTG | TMV_B (RdRP) | | To fuse TMV_A and TMV_B | |  |  |
| FQ-418 | CAATACTTTCCAGACATGCG**GTGATTTTTCTCTACAAGCGAA**GCTCTAGTTGTTAAAGGAAA | TMV_C (MP) | | To fuse TMV_A and TMV_C | |  |  |
| FQ-419 | AGCAAATAACTATCAAAAAAC | TMV_B (RdRP) | |  | |  |  |
| FQ-420 | GTTTTTTGATAGTTATTTGCT**GTGATTTTTCTCTACAAGCGAA**GCTCTAGTTGTTAAAGGAAAAG | TMV_C (MP) | | To fuse TMV_B and TMV_C | |  |  |
| FQ-493 | TTTCTCGAGCGCATGTCTGGAAAGTATTG | TMV_A (Rep) | | XhoI site (to build TMV_AAA) | |  |  |
| FQ-494 | AAACTCGAG**GTGATTTTTCTCTACAAGCGAA**TGCCGTGCATTCGCTTGC | TMV_A (Rep) | | XhoI site (to build TMV_AAA) | |  |  |
| FQ-495 | TTTCCATGGCGCATGTCTGGAAAGTATTG | TMV_A (Rep) | | NcoI site (to build TMV_AAA) | |  |  |
| FQ-496 | AAACCATGG**GTGATTTTTCTCTACAAGCGAA**TGCCGTGCATTCGCTTGC | TMV_A (Rep) | | NcoI site (to build TMV_AAA) | |  |  |
| FQ-497 | TTTGCGGCCGCCGCATGTCTGGAAAGTATTG | TMV_A (Rep) | | NotI site (to build TMV_AAA) | |  |  |
| FQ-542 | AAACCCGGG**CGACCGCGGTTTCTCTGTATAA**TGCCGTGCATTCGCTTGCAG | TMV_A (Rep) | | XmaI site; miR7122 target site | |  |  |
| FQ-543 | AAACCCGGG**TGGGAGGCATCGGAAAGACAAA**TGCCGTGCATTCGCTTGCAG | TMV_A (Rep) | | XmaI site; miR8036 target site | |  |  |
| FQ-407 | CCAATTGGGGCCCAACGTTCCTTATCTTTAATCATATTCC | tHSP | | To build TMV_A + miR173 | |  |  |
| FQ-409 | CCAAGGCCTTCTAGAAAAACCTCGACGAATTAATTCCAAT | P35S | | To build TMV_A + miR173 | |  |  |
| *pMIGS_TMV/PVX* | | | | | |  |  |
| FQ-421 | GTGTAGTAAGATCCGAGAGTG | TMV_C (MP) | | To amplify TMV_BC | | |  |
| FQ-422 | CACTCTCGGATCTTACTACAC**GTGATTTTTCTCTACAAGCGAA**GCCAAGGTGCGCGAGGTTTA | PVX_D (RdRP) | | To fuse TMV_BC with PVX_D | | |  |
| FQ-423 | ATCCGCGGGTTTCTTCTCATG | PVX_D (RdRP) | |  | | |  |
| FQ-424 | CATGAGAAGAAACCCGCGGAT**GTGATTTTTCTCTACAAGCGAA**CCAGCTAGCACAACACAGCC | PVX_E (CP) | | To fuse PVX_D with PVX_E | | |  |
| FQ-425 | TTTGCGGCCGCCTAGCTCTGCTGATGCCGTTG | PVX_E (CP) | | NotI site | | |  |
| *pMIGS_3X to pMIGS_15X* | | | | | |  |  |
| FQ-430 | GGG**GTGATTTTTCTCTACAAGCGAA**CGGCTGGGTCTG | Module 1 | |  | | |  |
| FQ-431 | TTTGCGGCCGCCCTGCAGGATTATCGTGGCCGGTG | Module 3 | | NotI and SbfI sites | | |  |
| FQ-432 | AAACCTGCAGG**GTGATTTTTCTCTACAAGCGAA**CTCGGCCACTCTG | Module 4 | | SbfI site | | |  |
| FQ-433 | TTTGCGGCCGCATCGATACATAGTTGGCGGTTAG | Module 6 | | NotI and ClaI sites | | |  |
| FQ-434 | AAAATCGAT**GTGATTTTTCTCTACAAGCGAA**GGATTGAATGCGAC | Module 7 | | ClaI site | | |  |
| FQ-435 | TTTGCGGCCGCTTAATTAACTACCGCAGGGTCCG | Module 9 | | NotI and PacI sites | | |  |
| FQ-436 | AAATTAATTAA**GTGATTTTTCTCTACAAGCGAA**GGTGATTTTTCGC | Module 10 | | PacI site | | |  |
| FQ-437 | TTTGCGGCCGCGGTACCCGCATCCCGGTCCAC | Module 12 | | NotI and KpnI sites | | |  |
| FQ-438 | AAAGGTACC**GTGATTTTTCTCTACAAGCGAA**ATTGTGGAAGGTG | Module 13 | | KpnI site | | |  |
| FQ-439 | TTTGCGGCCGCTGAGCGATTATGAAC | Module 15 | | NotI site | | |  |
| FQ-661 | AGAAATCGAT**GTGATTTTTCTCTACAAGCGAA**TGCC | TMV_A (Rep) | | ClaI site | | |  |
| FQ-662 | AGAATTAATTAA**GTGATTTTTCTCTACAAGCGAA**TGCC | TMV_A (Rep) | | PacI site | | |  |
| FQ-663 | AGAAGGTACC**GTGATTTTTCTCTACAAGCGAA**TGCC | TMV_A (Rep) | | KpnI site | | |  |
| *miRNA precursors* | | |  | |  | | |
| FQ-400 | CCCGGGTTCTTCTCACAAATAAACCC | miR173 | | XmaI site | | |  |
| FQ-401 | GCGGCCGCCGCATCAGATCTATCAAAC | miR173 | | NotI site | | |  |
| FQ-544 | AAACCCGGGGCTCCATAGTGGCCCTTACC | miR7122 | | XmaI site | | |  |
| FQ-545 | TTTGCGGCCGCTCGTAACAGAGAAGGCAGGAA | miR7122 | | NotI site | | |  |
| FQ-546 | AAACCCGGGTGAGCATTGATCCGTCAAAA | miR8036 | | XmaI site | | |  |
| FQ-547 | TTTGCGGCCGCCAAACGGATGGCAACATAAG | miR8036 | | NotI site | | |  |
| *RT-qPCR* | | | | | |  |  |
| Ben881 | AGCAAAGACCCCAACGAGAA | GFP | | Amplify the GFP-tagged TMV | | |  |
| FQ-004 | TTGTACAGCTCGTCCATGCC | GFP | | Amplify the GFP-tagged TMV | | |  |
| Ben0010F | CACTACCAACTGCCTTGCAC | GAPDH | | RT-qPCR housekeeping gene | | |  |
| Ben0011R | ATGAAGCAGCTCTTCCACCT | GAPDH | | RT-qPCR housekeeping gene | | |  |
| *sRNA blot probes* |  |  | |  | | |  |
| FQ-107 | AGGGGCCATGCTAATCTTCTC | U6 | |  | | |  |
| FQ-515 | TGCCGTGCATTCGCTTGCAG | TMV_A (Rep) | | For TMV_A probe | | |  |
| FQ-411 | TTTGCGGCCGCCGCATGTCTGGAAAGTATTG | TMV_A (Rep) | | For TMV_A probe | | |  |
| FQ-516 | CAGTTTTACTATGATAAGTG | TMV_B (RdRP) | | For TMV_B probe | | |  |
| FQ-413 | TTTGCGGCCGCAGCAAATAACTATCAAAAAAC | TMV_B (RdRP) | | For TMV_B probe | | |  |
| FQ-517 | GCTCTAGTTGTTAAAGGAA | TMV_C (MP) | | For TMV_C probe | | |  |
| FQ-415 | TTTGCGGCCGCGTGTAGTAAGATCCGAGAGTG | TMV_C (MP) | | For TMV_C probe | | |  |
| FQ-518 | GCCAAGGTGCGCGAGGTTTA | PVX_D (RdRP) | | For PVX_D probe | | |  |
| FQ-519 | ATCCGCGGGTTTCTTCTCATG | PVX_D (RdRP) | | For PVX_D probe | | |  |
| FQ-520 | CCAGCTAGCACAACACAGCC | PVX_E (CP) | | For PVX_E probe | | |  |
| FQ-425 | TTTGCGGCCGCCTAGCTCTGCTGATGCCGTTG | PVX_E (CP) | | For PVX_E probe | | |  |
| FQ-521 | TCTTCTCTAGCCGTGGTCG | AG | | For AG probe | | |  |
| FQ-361 | TTTGCGGCCGCGGCCATTTCCTTCAGCCTA | AG | | For AG probe | | |  |
| FQ-522 | TCTGATGATTCGATGGTGG | ELF3 | | For ELF3 probe | | |  |
| FQ-368 | TTTGCGGCCGCAGTCACCCCTTTGTTTGAC | ELF3 | | For ELF3 probe | | |  |
| FQ-571 | CGGCTGGGTCTGATTGTTAC | Modules 1-3 | | For Module 1 to 3 probe | | |  |
| FQ-386 | ATTATCGTGGCCGGTGCACG | Modules 1-3 | | For Module 1 to 3 probe | | |  |
| FQ-572 | CTCGGCCACTCTGAGGTG | Modules 4-6 | | For Module 4 to 6 probe | | |  |
| FQ-389 | ACATAGTTGGCGGTTAGATG | Modules 4-6 | | For Module 4 to 6 probe | | |  |
| FQ-573 | GGATTGAATGCGACGGGTGG | Modules 7-9 | | For Module 7 to 9 probe | | |  |
| FQ-392 | CTACCGCAGGGTCCGAAACC | Modules 7-9 | | For Module 7 to 9 probe | | |  |
| FQ-574 | GGTGATTTTTCGCATATTTG | Modules 10-12 | | For Module 10 to 12 probe | | |  |
| FQ-395 | CGCATCCCGGTCCACTCGTG | Modules 10-12 | | For Module 10 to 12 probe | | |  |
| FQ-575 | ATTGTGGAAGGTGTAGCTAC | Modules 13-15 | | For Module 13 to 15 probe | | |  |
| FQ-576 | TGAGCGATTATGAACAGGTG | Modules 13-15 | | For Module 13 to 15 probe | | |  |
| FQ-592 | GTGATTTCTCTCTGCAAGCGAA | miR173 | |  | | |  |
| FQ-593 | CGACCGCGGTTTCTCTGTATAA | miR7122a | |  | | |  |
| FQ-594 | TGGGAGGCATCGGAAAGACAAA | miR8036 | |  | | |  |
